# Characterization of Full-Length Transcriptome Sequences and Splice Variants of *Lateolabrax maculatus* by Single-Molecule Long-Read Sequencing and Their Involvement in Salinity Regulation

**DOI:** 10.3389/fgene.2019.01126

**Published:** 2019-11-15

**Authors:** Yuan Tian, Haishen Wen, Xin Qi, Xiaoyan Zhang, Shikai Liu, Bingyu Li, Yalong Sun, Jifang Li, Feng He, Wenzhao Yang, Yun Li

**Affiliations:** Key Laboratory of Mariculture, Ministry of Education, Ocean University of China, Qingdao, China

**Keywords:** *Lateolabrax maculatus*, Iso-Seq, full-length transcripts, alternative splicing, isoform, salinity regulation

## Abstract

Transcriptome complexity plays crucial roles in regulating the biological functions of eukaryotes. Except for functional genes, alternative splicing and fusion transcripts produce a vast expansion of transcriptome diversity. In this study, we applied PacBio single-molecule long-read sequencing technology to unveil the whole transcriptome landscape of *Lateolabrax maculatus*. We obtained 28,809 high-quality non-redundant transcripts, including 18,280 novel isoforms covering 8,961 annotated gene loci within the current reference genome and 3,172 novel isoforms. A total of 10,249 AS events were detected, and intron retention was the predominant AS event. In addition, 1,359 alternative polyadenylation events, 3,112 lncRNAs, 29,609 SSRs, 365 fusion transcripts, and 1,194 transcription factors were identified in this study. Furthermore, we performed RNA-Seq analysis combined with Iso-Seq results to investigate salinity regulation mechanism at the transcripts level. A total of 518 transcripts were differentially expressed, which were further divided into 8 functional groups. Notably, transcripts from the same genes exhibited similar or opposite expression patterns. Our study provides a comprehensive view of the transcriptome complexity in *L. maculatus*, which significantly improves current gene models. Moreover, the diversity of the expression patterns of transcripts may enhance the understanding of salinity regulatory mechanism in *L. maculatus* and other euryhaline teleosts.

## Introduction

With the development of high-throughput sequencing of the transcriptome, biologists have begun to pay more attention to multiple post-transcriptional processes of precursor-messenger RNA (pre-mRNA). Alternative splicing (AS), a key post-transcriptional processing of pre-mRNA, is prevalent in most eukaryotic organisms (Barbazuk et al., 2008; Pan et al., 2008; Kornblihtt et al., 2013; Yi et al., 2018; Zhang et al., 2019a), which makes an important contribution to the enhancement of the functional complexity of the transcriptome (Graveley, 2001; Reddy et al., 2013; Abdel-Ghany et al., 2016). Transcriptome complexity plays an important role in increasing the coding capacity of genes, generating proteome diversity, regulating gene expression, cellular physiological and developmental processes (Lareau et al., 2004; Abdel-Ghany et al., 2016; Wang et al., 2016). It has been shown that over 90% of multi-exonic genes in human (*Homo sapiens*) (Pan et al., 2008), 46% in fruit fly (*Drosophila melanogaster*) (Hansen et al., 2009), and 61% in the model plant thale cress (*Arabidopsis thaliana*) are alternatively spiced (Marquez et al., 2012). Although the functional significance of most spliced isoforms has yet to be fully elucidated, several studies suggest that AS is a profound regulatory process involved in organismal function. For instance, in thale cress, zinc-induced facilitator-like 1 can produce two spliced isoforms, one that regulates stomatal movement and another that influences cellular auxin transport (Reddy et al., 2013). Coincidentally, the *Bcl-x* gene in fruit fly yields two different isoforms, one of which inhibits apoptosis, while the other activates apoptosis (Chang et al., 2004). In addition, alternative polyadenylation (APA), another post-transcriptional regulatory events in which RNA molecules with different 3’ ends originate from distinct polyadenylation sites of a single gene, is emerging as a mechanism widely used to regulate gene expression (Chen et al., 2017b). APA events may alter sequence elements and/or the coding capacity of transcripts, and could be considered as a mechanism that adds another layer to regulation of transcriptome diversity (Shen et al., 2011; Abdel-Ghany et al., 2016; Ha et al., 2018).

However, due to technical limitations, most information on these post-transcriptional regulatory events remains limited. Although data from short-read sequencing have accumulated over recent years, it remains an immense challenge to obtain full-length (FL) sequences for each RNA because of difficulties in the short read-based assembly, which limits the identification and prediction of post-transcriptional events (Wang et al., 2016; Chen et al., 2017a). In the last few years, Pacific BioSciences (PacBio) single-molecule real-time sequencing has been introduced (Rhoads and Au, 2015). The PacBio isoform sequencing (Iso-Seq) platform can directly produce FL transcripts without an assembly process, providing superior evidence for comprehensive analysis of splice isoforms of each gene and improving the annotation of existing gene models (Tilgner et al., 2014; Gordon et al., 2015; Wang et al., 2016). Recently, Iso-Seq has led to the discovery of thousands of novel genes and alternatively spliced isoforms in human (Au et al., 2013), mouse (*Mus musculus*) (Karlsson and Linnarsson, 2017), rabbit (*Oryctolagus cuniculus*) (Chen et al., 2017a), sorghum (*Sorghum bicolor*) (Abdel-Ghany et al., 2016), and maize (*Zea mays*) (Wang et al., 2016). This finding indicates that Iso-Seq is sensitive to detecting FL transcripts and serves as a valuable resource for transcriptome complexity research. In addition, the studies in aspen (*Populus tremuloides*) (Chao et al., 2019), strawberry (*Fragaria vesca*) (Li et al., 2017), and pig (*Sus scrofa*) (Li et al., 2018) also provide strong evidence that Iso-Seq could complement short-read sequencing with cataloguing and quantifying eukaryotic transcripts.

Spotted sea bass (*Lateolabrax maculatus*) is a euryhaline teleost fish naturally distributed in the northwestern Pacific Ocean, especially along the Chinese coast, reaching south to the borders of Vietnam and north to Korea and Japan (Zhang et al., 2001; Tseng and Hwang, 2008; Seo et al., 2016). It is considered as one of the most popular economic fishes because of its high nutritive value and pleasant taste. Since the release of the draft reference genome of *L. maculatus* (Shao et al., 2018; Chen et al., 2019), more functional genes have been discovered. However, most of the existing gene models are derived from *in silico* prediction with a lack of reliable annotation of alternative isoforms and untranslated regions, which would prevent accurate evaluation of transcriptome complexity (Chen et al., 2017a). Hence, our study is crucial for facilitating the biological research of *L. maculatus*.

Salinity represents a major abiotic stress and critical environmental factor that directly affects the survival, growth, development, reproduction, and physiological functions of all aquatic organisms (Kultz et al., 2013; Kultz, 2015). *L. maculatus*, a typical euryhaline fish, is capable of inhabiting freshwater, brackish water, seawater, and hypersaline water (Kim et al., 1998). It has been documented that *L. maculatus* can tolerate a considerable range of external salt concentrations (0–45 ppt) and maintain constant internal osmotic homeostasis (Zhang et al., 2019b). Hence, it provides an excellent model with which to identify and characterize osmoregulatory mechanisms. In spotted sea bass, RNA-Seq analysis has been performed to identify hundreds of genes involved in salinity adaptation and osmoregulation (Zhang et al., 2017). In addition, previous RNA-Seq studies in other aquaculture fish species, including Asian seabass (*Lates calcarifer*), striped catfish (*Pangasianodon hypophthalmus*), Mozambique tilapia (*Oreochromis mossambicus*), and Nile tilapia (*Oreochromis niloticus*), have identified several differentially expressed genes in response to distinct salinity concentrations (Xia et al., 2013; Thanh et al., 2014; Ronkin et al., 2015), which were considered as candidate osmoregulatory genes. However, due to limitations of technology, RNA-Seq lacks the ability to accurately quantify the transcripts or isoforms (Steijger et al., 2013). In this study, we applied Iso-Seq to uncover post-transcriptional regulatory events in *L. maculatus* and combined with gill RNA-Seq to investigate salinity regulation at the transcript level. It was the first time that Iso-Seq was applied in aquaculture teleosts, providing the first comprehensive view of transcriptome complexity in *L. maculatus* and characterizing differentially expressed transcripts (DETs) involved in osmoregulatory mechanisms, which refines the annotation of the reference genome and serves as a valuable reference for future Iso-Seq studies.

## Materials and Methods

### Ethics Statement

All experiments involving animals were conducted according to the guidelines and approved by the respective Animal Research and Ethics Committees of Ocean University of China (Permit Number: 20141201). The field studies did not involve any endangered or protected species.

### Fish Sample Collection for Iso-Seq

Three *L. maculatus* adults (body length: 44.92 ± 4.63 cm, body weight: 551.23 ± 7 9.84 g) were obtained from Kiaochow Bay of the Yellow Sea, China. The fish individuals were anesthetized with MS-222 and rapidly dissected for 13 tissues including brain, hypophysis, gill, heart, liver, stomach, intestine, kidney, spleen, gonad, muscle, fin, and skin. Then, these tissues were immediately frozen in liquid nitrogen and transferred to −80°C refrigerator until the extraction of RNA.

### RNA Extraction

Total RNA was extracted using TRIzol reagent (Invitrogen, CA, USA) according to the manufacturer’s instructions and digested with RNase-free DNase I (Takara, Shiga, Japan) to remove genomic DNA contamination. The reagents and instruments involved in this experiment were treated with 0.1% (vol/vol) diethylpyrocarbonate (DEPC) to maintain RNase-free conditions. The concentration and integrity of RNA was monitored using NanoDrop ND-1000 (NanoDrop Technologies, DE, USA) and 1% agarose gel electrophoresis, respectively. Agilent Bioanalyzer 2100 system (Agilent Technologies, CA, USA) was used to assess the quality of extracted RNA. All RNA samples from three *L. maculatus* were equally pooled together for following PacBio Iso-Seq.

### Iso-Seq Library Construction and Sequencing

According to the Iso-Seq protocol, 1 µg total RNA was transcribed to generate full-length cDNA using the SMARTer PCR cDNA Synthesis Kit (Clontech, CA, USA). Then, the cDNA was amplified using the advantage 2 PCR kit (Clontech, CA, USA), and PCR products were purified with AMpure PB beads (Beckman Coulter, CA, UAS). Purification was followed by size selection using the BluePippinTM Size Selection System (Sage Science, MA, USA) of the following bins: 1-2, 2-3 and 3-6 kb. The three libraries were then constructed using SMRTbell Template Prep kit (Pacific Biosciences, CA, USA). Before sequencing, the quality of the libraries was assessed by Agilent Bioanalyzer 2100 system (Agilent Technologies, CA, USA) and Qubit fuorometer 2.0 (Life Technologies, CA, USA). Libraries were prepared for sequencing by annealing a sequencing primer and adding polymerase to the primer annealed template. The polymerase-bound template was bound to MagBeads and a total of 6 SMRT cells were sequencing on PacBio RS II platform using P6-C4 chemistry (2 cell each library).

### PacBio Long-Read Processing

PacBio polymerase reads were processed into error corrected reads of insert (ROIs) using SMART Analysis v2.3 (https://www.pacb.com/products-and-services/analytical-software) with min-Full Pass > = 0 and min-Predicted Accuracy >75%. After ROIs <50 bp were discarded, they were classified into full-length non-chimeric (FLNC) and non-full-length (NFL) reads based on the presence of the poly(A) tail signal and the 5’ and 3’ cDNA primers. FLNC reads were clustered into consensus sequences using the Iterative Clustering for Error Correction (ICE) algorithm (https://www.pacb.com/products-and-services/analytical-software). Combined with NFL reads, consensus sequences were then polished in clusters using Quiver (Chin et al., 2013). Based on the criteria of post-correction accuracy >99%, consensus sequences were divided into high-quality and low-quality sequences. To improve the accuracy of consensus sequences, low-quality sequences were corrected by the above Illumina clean reads using Proovread v2.13.13 with default parameters (Thomas et al., 2014). Consensus sequences were mapped to the reference genome of *L. maculatus* (NCBI BioProject ID: PRJNA407434) using Genomic Mapping and Alignment Program (GMAP) (Wu and Watanabe, 2005). Mapped sequences were further collapsed using the pbtranscript-ToFU package (http://github.com/PacificBiosciences/cDNA_primer/) with min-coverage = 85% and min-identity = 90% to generate non-redundant transcripts.

### Alternative Splicing (AS) Analysis

AStalavista v3.2 software with default parameters was employed to determine the AS events in above obtained non-redundant transcripts (Foissac and Sammeth, 2007). The non-redundant transcripts were further classified into five major types of AS events following the rules in previous publication (Wang et al., 2016), namely Intron retention, Exon skipping, Alternative 3’ splice site, Mutually exclusive exon, and Alternative 5’ splice site.

### Alternative Polyadenylation (APA) Identification

In our study, FLNC reads were selected to identify APA sites using Transcriptome Analysis Pipeline for Isoform Sequencing (TAPIS pipeline v1.2.1, default parameters) (Abdel-Ghany et al., 2016). The qualified APA for a gene must be supported by at least two FLNC reads aligned to the gene loci.

### Long Non-Coding RNA (LncRNA) and Simple Sequence Repeat (SSR) Analysis

Four computational approaches, including Coding-Non-Coding-Index (CNCI, v2), Coding Potential Calculator (CPC, v1), Coding Potential Assessment Tool (CPAT, v1.2), and Pfam (v1.5), were combined to identify non-protein coding RNA candidates from the non-redundant transcripts. Transcripts with lengths more than 200 bp and more than two exons were selected as lncRNAs candidates and further screened using CPC, CNCI, CPAT, and Pfam that have the power to distinguish the protein-coding genes from the non-coding genes. The relationship between lncRNAs and target genes were predicted based on their position (<100 kb upstream or downstream) and base complementary using lncTar target gene prediction tool (v1.0) with default parameters (Li et al., 2015).

The simple sequence repeats (SSR) were identified using Microsatellite identification tool (MISA, v1.0) with default parameters (Beier et al., 2017). Only non-redundant transcripts that were > = 500 bp in size were selected for SSR detection. A total of seven SSR types were identified, namely, Mono-nucleotide, Di-, Tri-, Tetra-, Penta-, Hexa-, and compound SSR, respectively.

### Fusion Transcripts and Transcription Factors (TFs) Detection

Consensus sequences from PacBio Iso-Seq were selected for fusion transcripts identification. A fusion transcript is a chimeric RNA encoded by a single fusion gene or by two different genes that are subsequently joined by trans-splicing. The criteria used to identify candidate fusion transcripts as follows A) map to two or more loci; B) minimum coverage for each locus is 5% and minimum coverage in bp is > = 1bp; C) total coverage is > = 95%; D) distance between the loci is at least 10 kb (Wang et al., 2016; Li et al., 2018).

Animal TFDB 3.0 (http://bioinfo.life.hust.edu.cn/AnimalTFDB/) was set as the reference transcription factor database. The algorithm HMMER 3.0 software was used to identify TFs and assign transcripts to different families (Eddy, 2009).

### Functional Annotation

The non-redundant transcripts were aligned against several protein and nucleotide databases, including Clusters of Gene Ontology (GO), Kyoto Encyclopedia of Genes and Genomes (KEGG), conserved Protein families or domains (Pfam), Swiss-prot, NCBI non-redundant proteins (NR), and non-redundant nucleotide (NT) databases, using BLASTX (v2.2.26) with cutoff E-value < = 1e^-5^ (Camacho et al., 2009).

### Fish and Experimental Treatments for RNA-Seq

*L. maculatus* adults (body length: 21.92 ± 3.17 cm, body weight: 158.23 ± 18.77 g) were acquired from Shuangying Aquaculture Company (Dongying, Shandong Province, China) and acclimated for a week. Water temperature (13.5–14.5°C), pH (7.8–8.15), salinity (30 ppt), and DO (6.7–7.5 mg/L) were stabilized during the acclimation. After acclimation, 90 individuals were randomly divided into two groups: freshwater (FW, 0 ppt) and seawater group (SW, 30 ppt) in triplicate tanks at the density of 15 individuals per tank. After 30 days of breeding, three individuals per tank were anesthetized with MS-222 and rapidly sampled for gill tissues, which were frozen in liquid nitrogen and transferred to −80°C refrigerator until the extraction of RNA.

### RNA-Seq Library Construction and Sequencing

Total RNA was extracted using TRIzol method mentioned above (the section *RNA Extraction*). Equal amounts of RNA from the gill tissues of three individuals (500 ng per individual) from the same tank were pooled as one sample to minimize the variation among individuals. A total of 6 libraries (3 replicated samples × 2 treatment groups) were constructed using the TruSeq™ RNA Sample Prep Kit (Illumina, CA, USA). The libraries were sequenced on Illumina HiSeq 4000 platform and 150 bp paired-end raw reads were generated. Then, the raw reads were processed using Trimmomatic software (Bolger et al., 2014) and clean reads were obtained for the following analysis.

### Differentially Expressed Transcript (DETs) Analysis

To estimate the expression level of transcripts, the Iso-Seq database was added to the genome database to construct a new database with a modified general feature format (GFF) file. Then, the clean reads from the above RNA-Seq were mapped to the new database using STAR software (v2.5.3) (Dobin et al., 2013). The Cuffquant and Cuffnorm modules of the Cufflinks program (v2.2.1) were used to quantify transcript abundance based on the mapped results (Trapnell et al., 2010). When all the splicing junctions of transcripts were supported by clean reads, it was defined as an expressed transcript. For the reads mapped to multiple isoforms derived from the same gene, they were distributed according to uniquely mapped reads using Cuffquant and Cuffnorm modules. The mapped reads were counted and subsequently normalized to fragments per kilobase of transcript per million fragments mapped (FPKM) as the expression value. Differential expression analysis in the FW and SW environments was performed using the DEseq R package (1.10.1). The *P*-values were adjusted using Benjamini and Hochberg’s approach for controlling the false discovery rate (FDR). The FDR <0.05 and |fold change| > = 2 were set as the threshold for significantly differentially expressed transcripts (DETs).

### Validation Experiments

For RT-PCR validation of AS events, fusion transcripts, and novel transcripts, total RNA was reverse-transcribed to cDNA using PrimeScript RT reagent kit (Takara, Shiga, Japan) following the manufacturer’s instructions. 10×diluted cDNA was served as the template and Fastpfu reagent kit (TransGen, Beijing, China) was used for RT-PCR amplification. Transcript-specific primers were designed to span the predicted splicing events using Primer 5 software (**Supplementary Table 1**). PCR conditions were 5 min at 94°C followed by 35 cycles of 94°C for 30 s, Tm for 30 s, 72°C for a time period that depends on the product sizes, and 72°C for 10 min. PCR products were monitored on 1% agarose gel stained by GelStain (TransGen, Beijing, China). For the APA validation, 1 µg RNA was used to synthesize first-strand cDNA using the SMART™ RACE cDNA Amplification Kit (Clontech, California, USA). Gene-specific primers (**Supplementary Table 1**) were designed for the 3’ rapid amplification of cDNA ends (3’ RACE). PCRs were performed using Taq DNA Polymerase (Clontech, California, USA) following touchdown PCR cycling conditions: denaturation step at 94°C for 3 min, followed by 20 cycles of 94°C for 15 s and at a range of annealing temperature from 60 to 50°C, decreasing 0.5°C each cycle and 72°C for 40 s, and finally ended with 10 min at 72°C for extension. PCR products were also monitored on 1% agarose gel stained by GelStain (TransGen, Beijing, China). Finally, the products were purified, subcloned into T1 vector, propagated in *Escherichia coli* DH5a, and sequenced by the Sanger method.

Quantitative real-time PCR (qPCR) analysis was employed to verify differentially expressed transcripts. Total RNA was isolated from the gill tissues of fish exposed to freshwater and seawater in previous salinity challenge experiment. cDNA was synthesized using the PrimeScript RT reagent kit (TaKaRa, Shiga, Japan). All transcripts-specific primers for qPCR were designed using Primer 5 software and listed in **Supplementary Table 2**. SYBR Premix Ex Taq kit was used for qPCR (Takara, Shiga, Japan). Each PCR reaction consisted of 2 µl cDNA, 10 µl SYBR premix Ex Taq, 0.4 µl of each forward and reverse primers, 0.4 µl ROX Reference Dye, 6. 8µl ddH2O to a final volume of 20 µl. qPCR was performed on the Applied Biosystems 7300 machines (Applied Biosystems, CA, USA) under the following conditions: 95°C for 30 s and 40 cycles of 95°C for 5 s, 55°C for 30 s, and 72°C for 30 s. The relative expression levels of transcripts were normalized by 18S ribosomal RNA. 2^-ΔΔCT^ method was used for subsequent analysis. The correlation coefficient between differential expressed transcripts and qPCR were determined by SPSS 17.0 software (Bryman and Cramer, 2011). One-way ANOVA was conducted followed by Duncan’s multiple tests to identify significance differences when *P* < 0.05.

## Results

### PacBio Iso-Seq and Bioinformatic Analysis

**Figure 1:**
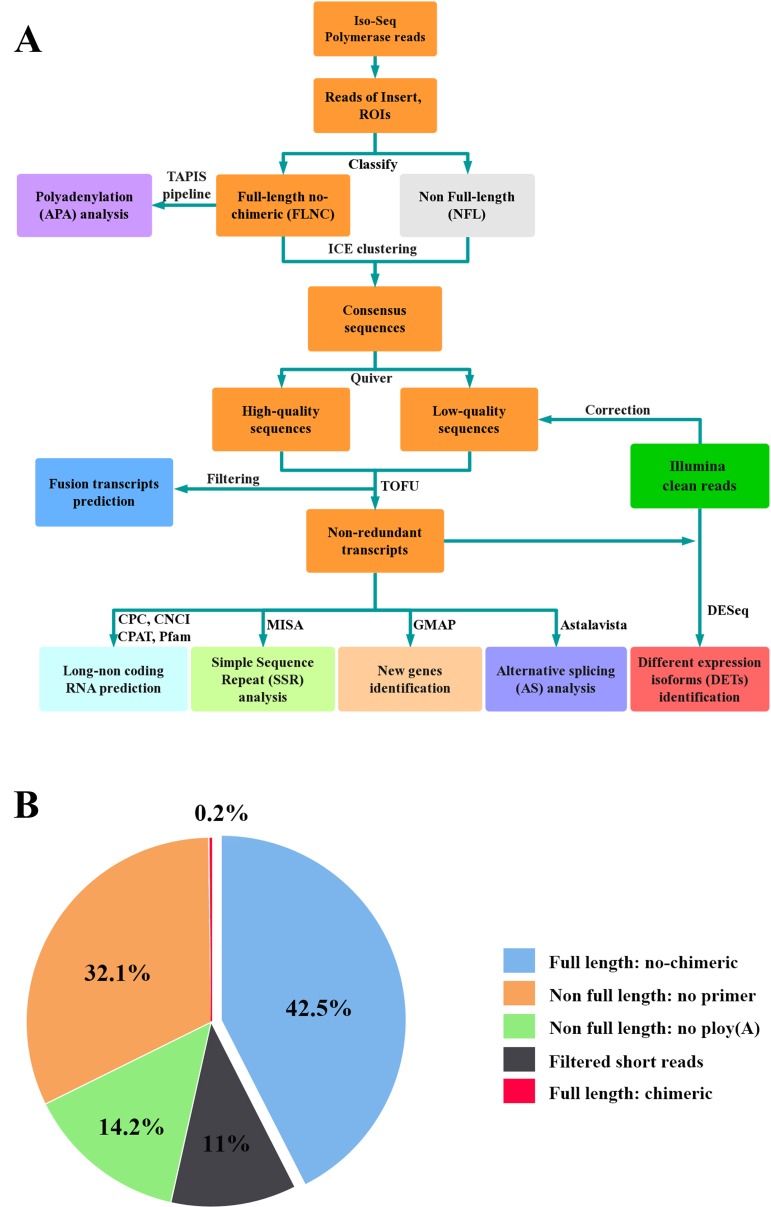
Iso-Seq analysis pipeline and ROIs classification. **(A)** Pipeline of bioinformatic analysis in Iso-Seq. **(B)** Pie chart of ROIs classification for Iso-Seq. Full-length reads: containing 5’ primer, 3’ primer and the poly **(A)** tails. Filtered short reads: length 300 bp; Chimeric reads: containing artificial concatemers.

**Figure 2:**
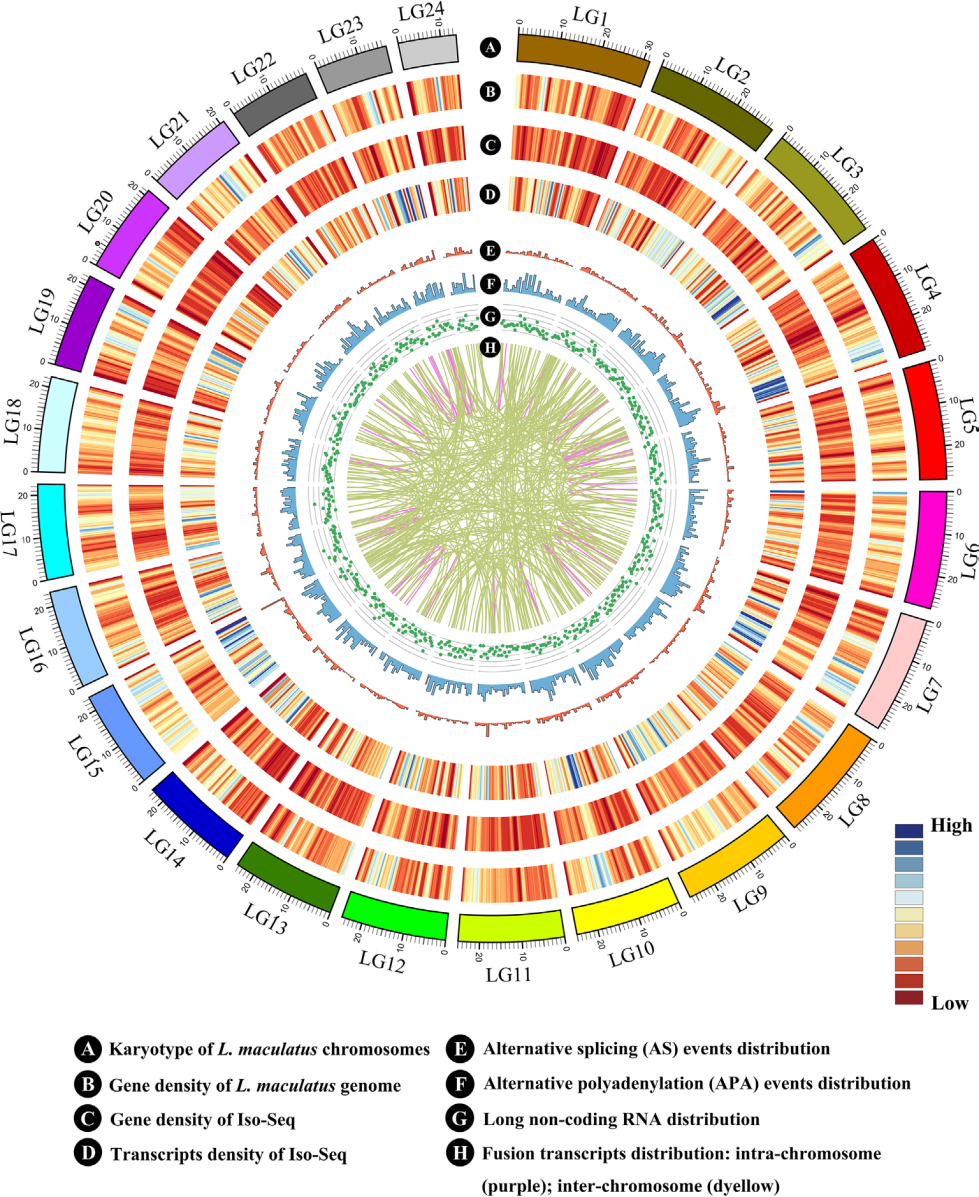
Circos visualization of *L. maculatus* genome and Iso-Seq results. **(A)** Karyotype of the *L. maculatus* chromosome; **(B)** Gene density of the *L. maculatus* genome; **(C)** Gene density of Iso-Seq; **(D)** Transcript density of Iso-Seq; **(E)** Alternative splicing (AS) events distribution; **(F)** Alternative polyadenylation (APA) events distribution; **(G)** Long non-coding RNA distribution; and **(H)** Fusion transcripts distribution: intra-chromosome (purple); inter-chromosome (deyellow). The distribution was calculated in a 1-Mb sliding window at 20 kb intervals.

In total, six SMRT cells, including three size-fractionated libraries (1–2, 2–3, and 3–6 kb), were used for Iso-Seq, yielding 13.42 Gb of clean data. A specific bioinformatic analysis pipeline for our Iso-Seq data is outlined in **Figure 1A**. In detail, 363,371 ROIs were retained after filtering, and the mean length was 3,120 bp (**Table 1**). The density plot of the length of the ROIs showed three obvious peaks, which was consistent with the size of the three libraries (**Supplementary Figure 1**). ROIs were further classified into FLNC and NFL reads based on the presence of 5’ primer, 3’ primer, and poly(A) tails. A total of 39.79%, 44.88%, and 44.77% of ROIs were qualified as FLNC reads in the 1–2, 2–3, and 3–6 kb libraries (**Table 1**), respectively, with an average FLNC ratio of 42.5% (**Figure 1B**). ICE was applied for sequence clustering, yielding 60,573 consensus sequences (**Table 1**). Combined with NFL reads, these consensus sequences were corrected by Quiver. A total of 68.92% (41,744) of the sequences were defined as high-quality sequences. The remaining 18,829 consensus sequences were defined as low-quality sequences, which were subsequently corrected by Illumina clean reads. Finally, these consensus sequences were collapsed by the TOFU process, yielding 28,809 non-redundant transcripts retained for the following study (**Table 1**). The reference genome and Iso-Seq data information was shown in **Figure 2**.

**Table 1:** Statistics of Iso-Seq data in *L. maculatus.*

Library	1-2kb	2-3kb	3-6kb	Total
SMRT cell	2	2	2	6
Number of subreads	2,933,418	1,466,540	869,243	5,269,201
Mean length of subreads	1,835	3,178	3,877	2,546
Number of ROIs	156,048	122,203	85,120	363,371
Mean length of ROIs	2,540	3,482	3,663	3,120
Mean quality of ROIs	0.92	0.90	0.87	0.90
Mean Number of Passes	12	7	5	8.7
Number of FL reads	62,090	54,840	38,112	154,969
Number of FLNC reads	61,392	54,818	38,093	154,249
Mean length of FLNC reads	1,258	2,159	3,337	2,091
FL percentage	39.79%	44.88%	44.77%	42.50%
Number of consensus sequences	24,188	17,561	18,824	60,573
Mean length of consensus sequences	1,321	2,360	3,712	2,365
High-quality sequences	21,390	12,434	7,920	41,744
Low-quality sequences	2,798	5,127	10,904	18,829
Non-redundant transcripts	–	–	–	28,809

### Transcripts and Alternative Splicing (AS) Events

**Figure 3:**
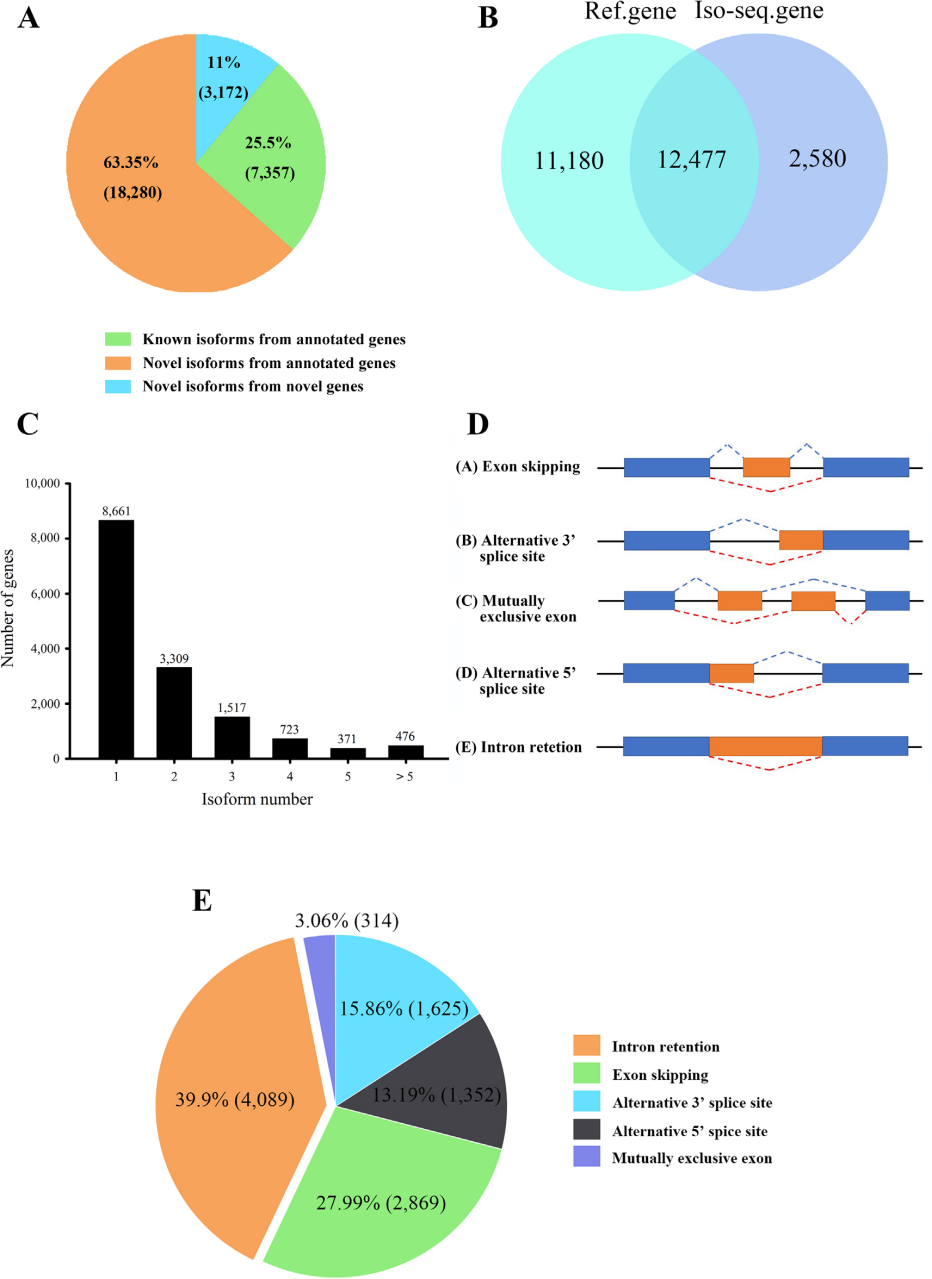
Characteristics of transcripts and alternative splicing (AS) events of *L. maculatus*. **(A)** Numbers of three types of transcripts. **(B)** Venn diagram showing the common and unique genes from the *L. maculatus* genome and Iso-Seq. **(C)** Distribution of the number of isoforms per gene. **(D)** Schematic diagram of five types of AS events. **(E)** Pie chart showing the numbers of five AS events in Iso-Seq.

A total of 28,809 non-redundant transcripts were compared against *L. maculatus* reference genome. In total, 88.9% of non-redundant transcripts (25,637) were aligned to 12,477 annotated gene loci (**Figure 3A**), covering 52.7% of the *L. maculatus* genome loci (23,657). Based on the splice sites of the genome and structures of transcripts, a total of 25,637 transcripts annotated in the genome were further classified into two groups as follows (**Figure 3A**): 1) known isoforms (7,357, 25.5%) sharing the same splice sites with the existing *L. maculatus* gene models; and 2) novel isoforms (18,280, 63.5%) that share at least one splice site with existing *L. maculatus* gene models but differ in other splice sites. Typical examples were shown in **Supplementary Figure 2A**. The remaining 3,172 transcripts (11.1%) were absent from any annotated gene loci in the *L. maculatus* genome and were identified as novel isoforms from novel genes. The 3,172 novel isoforms were clustered into 2,580 gene loci defined as novel genes (**Figure 3B**, **Supplementary Figure 2B**). To further investigate the homology and annotation, these novel gene loci were aligned against the Swiss-Prot and NR databases. A total of 24.96% of novel gene loci (644) were annotated in the Swiss-Prot protein database, and 43.64% of novel loci (1,126) were in NR database, which exhibited their homology to other species. The remaining genes absent in the databases were likely species-specific genes in *L. maculatus*. Four novel isoforms from novel genes were randomly selected for validation by RT-PCR (**Supplementary Figure 3A**).

A total of 15,057 gene loci (12,477 existing gene loci and 2580 novel gene loci) were identified in our Iso-Seq data, of which 6,396 (42.5%) were found to generate at least two different isoforms (**Figure 3C**). Notably, 476 out of 15,057 (3.2%) genes produced more than 5 isoforms for each gene loci, generating a total of 6,087 unique isoforms that accounted for 21% of the total Iso-Seq transcripts. To investigate the potential function of the genes with numerous isoforms, KEGG pathway enrichment analysis was performed for 847 genes harboring more than 4 isoforms. The results revealed that the most enriched pathways were related to the phagosome, apoptosis, and the AGE-RAGE signaling pathway (**Supplementary Figure 4**).

During AS events, splice sites are used with greater or lesser frequency to produce messages that differ in their exon content and structure (Liu et al., 2017). Although this happens frequently, only a few of the AS events have been reported in aquaculture species. In this study, a total of 10,249 AS events were detected from the Iso-Seq database and further classified into five main types (**Figures 2E** and **3D, E**, **Supplementary Figures 5A–E**). Strikingly, intron retention (39.9%, 4,089) was the most enriched type of AS event, and exon skipping (27.99%, 2,869) was the second most prevalent AS event. The number of the two AS types accounted for more than half (67.86%) of the total AS events in *L. maculatus*.

**Figure 4:**
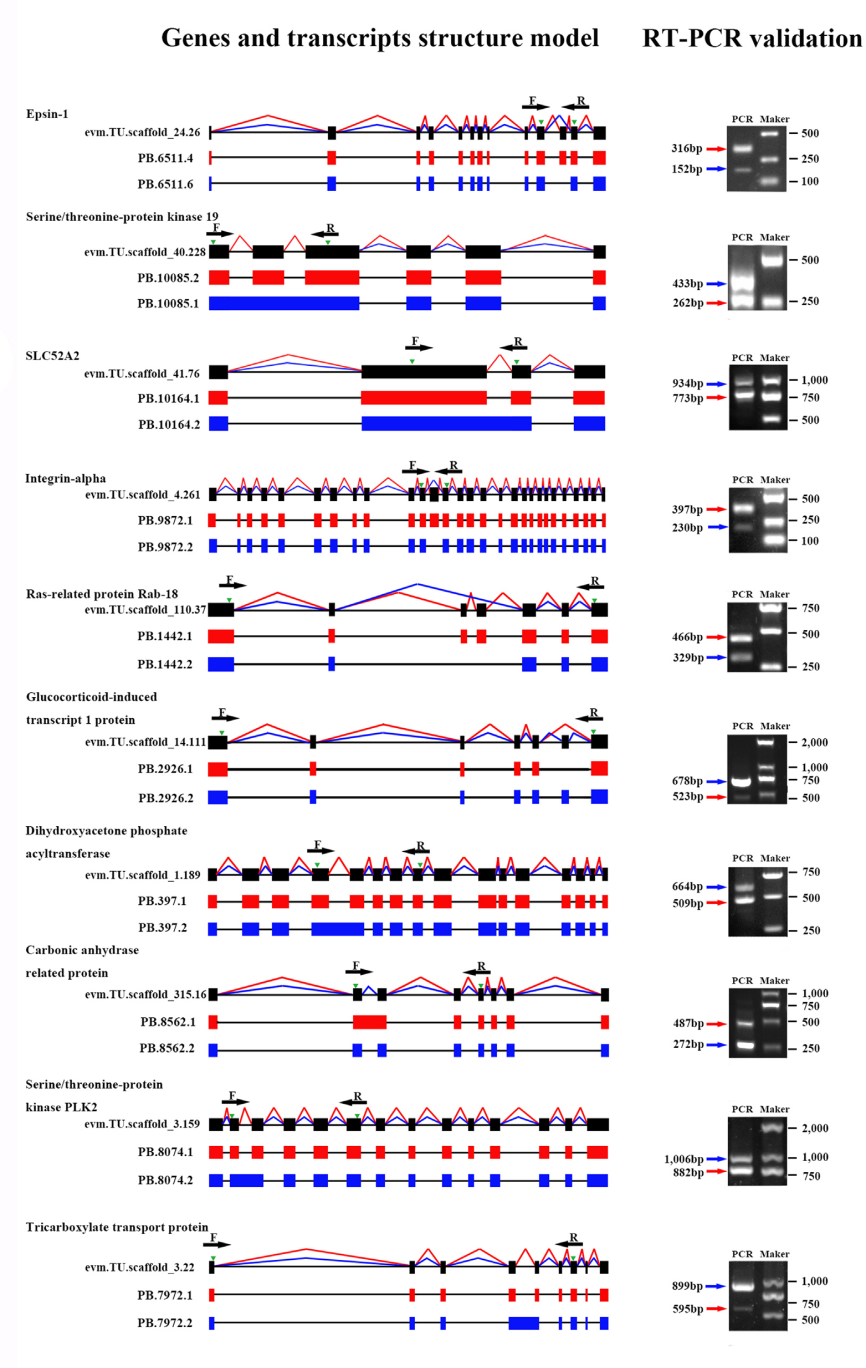
Validation of alternative splicing (AS) events by RT-PCR. Ten AS events were randomly selected for RT-PCR validation. Gene name, gene ID, and transcript ID were shown in order to the left of the gene model. Splicing events of different transcripts were shown by red or blue lines connecting exons, and green arrowheads indicated the position of PCR primers (F, forward and R, reverse). Introns are represented by black lines. In the agarose gel images, the red or blue arrowheads indicated the PCR products generated from the corresponding transcripts.

To verify the accuracy of isoforms identified by Iso-Seq, 10 genes with predicted AS events were randomly selected, and the existence and size of isoforms were validated by RT-PCR. Primers were designed in the overlapping regions of various transcripts derived from the same gene. The experimental results demonstrated that the amplified product sizes were consistent with predicted target fragments by Iso-Seq, confirming the credibility of our Iso-Seq data (**Figure 4**).

### Alternative Polyadenylation (APA) Events

**Figure 5:**
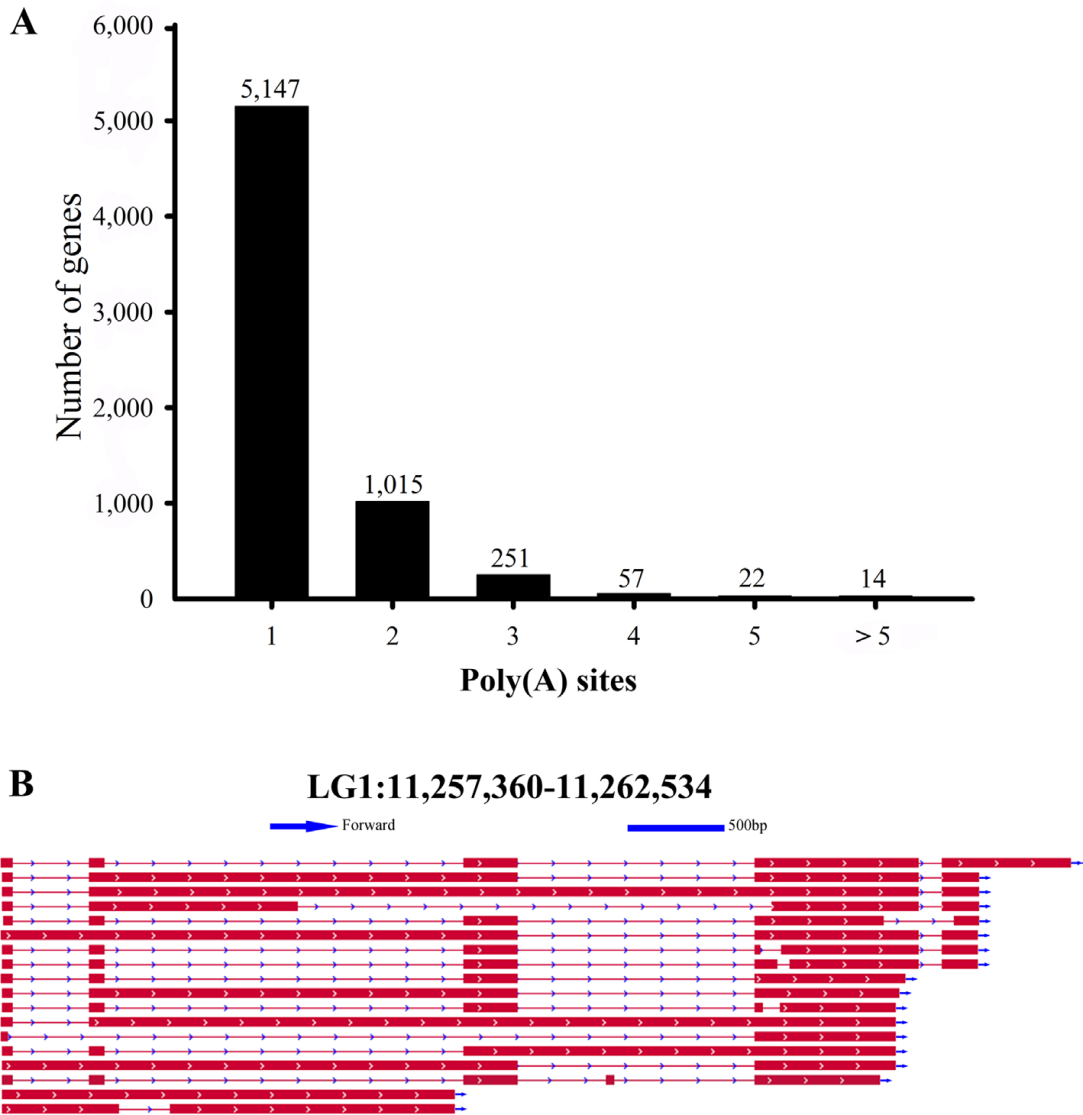
Statistics of identified Alternative polyadenylation (APA) events. **(A)** Distribution of the number of poly(A) sites per gene. **(B)** IGV view of *haptoglobin* gene with APA events.

In our Iso-Seq data, the TAPIS pipeline was used to detect APA events in *L. maculatus*. The qualified gene loci for APA must be supported by at least two aligned FLNC reads. Of the 6,506 detected genes with evidence of a poly(A) site, 5,147 genes (79.11%) were found to contain a single poly(A) site (**Figures 2F** and **5A**). The remaining 1,359 (20.89%) genes contained two or more detected poly(A) sites, and 14 genes were predicted to generate more than 5 poly(A) sites. An example, the transcripts structure of *haptoglobin* gene, which contained several distinct poly(A) sites, was illustrated in **Figure 5B**. Additionally, a gene with APA events was randomly selected for the validation experiment using 3’RACE and Sanger sequencing (**Supplementary Figure 3B**).

### Long Non-Coding RNA (LncRNA)

**Figure 6:**
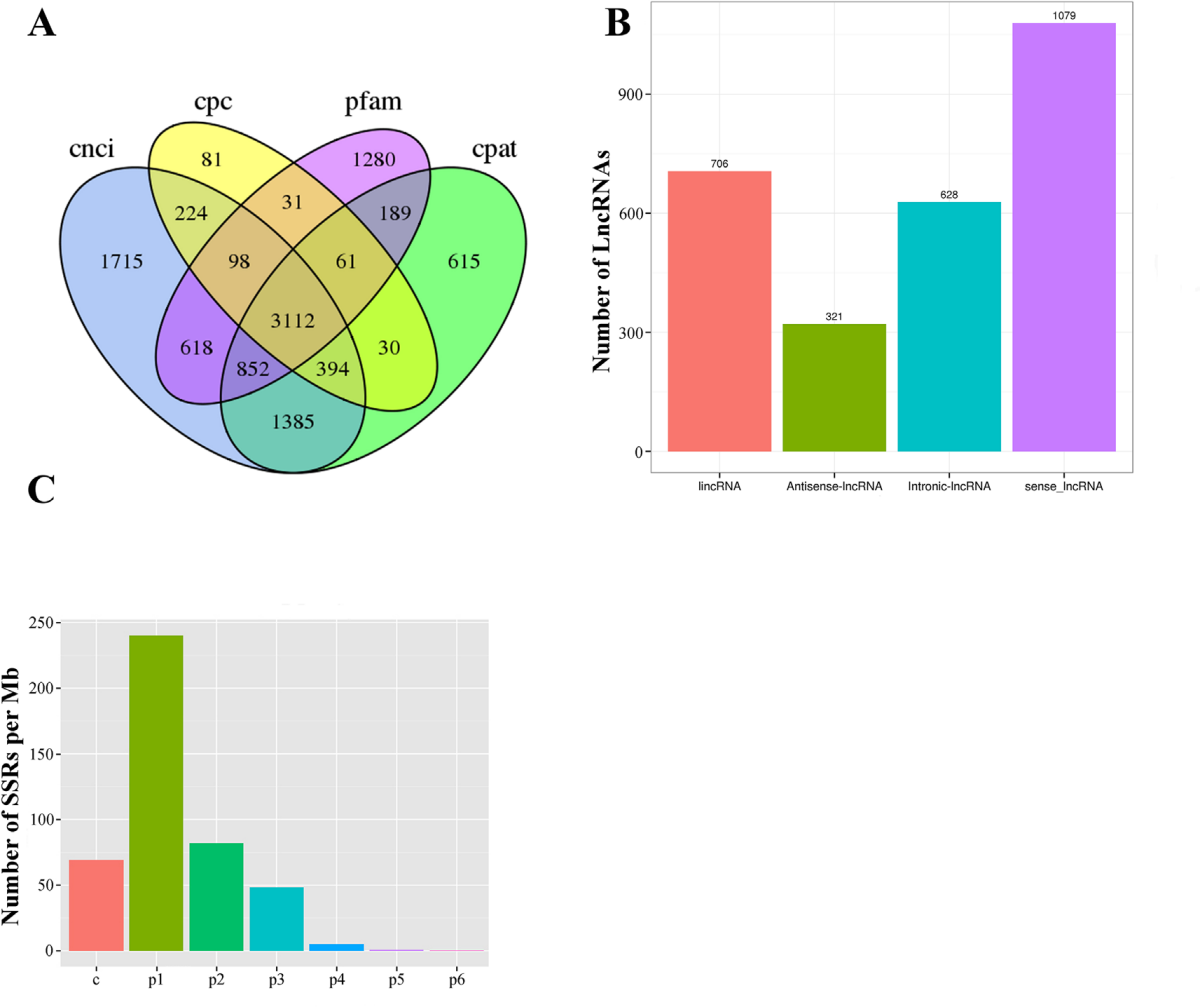
Statistics of long non-coding RNA and SSR in *L. maculatus*. **(A)** Venn diagram showing the number of lncRNAs annotated by four computational approaches including CPC, CNCI, CPAT, and Pfam. **(B)** The number of four types of lncRNA based on biogenesis positions relative to protein-coding genes. **(C)** Density of SSR motifs. c, compound SSR; p1: Mono-nucleotide SSR; p2, Di-nucleotide SSR; p3, Tri-nucleotide SSR; p4, Tetra-nucleotide SSR; p5, Penta-nucleotide SSR; p6, Hexa-nucleotide SSR.

In our Iso-Seq database, a total of 3,112 lncRNAs were ultimately identified by intersection analysis of four computational approaches, including CNCI, CPC, CPAT, and Pfam (**Figures 2G** and **6A**). Based on biogenesis positions relative to protein-coding genes of the genome of *L. maculatus*, 2,734 (87.85%) lncRNAs were further divided into four categories: 22.69% (706) were generated from intergenic regions (lincRNA), 20.18% (628) from intronic regions (intronic-lncRNA), 10.32% (321) from the antisense strand (antisense-lncRNA), and 34.67% (1,079) from the sense strand (sense-lncRNA) (**Figure 6B**). The relationship between lncRNAs and target genes was predicted based on their position (< 100 kb upstream or downstream) and base complementary. A total of 13,566 protein-coding gene loci were screened in the 100 kb upstream or downstream of 3,007 lncRNAs. In total, 909 lncRNAs were found to have a base-pairing interaction with 14,080 mRNAs.

### Simple Sequence Repeat (SSR)

Of 21,432 selected novel transcripts (novel isoforms from known gene loci and novel transcripts from novel gene loci), a total of 13,450 transcripts were found to contain 29,690 SSR motifs (**Supplementary Table 3**). Additionally, more than half of the SSR transcripts (7,401, 55.03%) contained at least 2 SSR motifs, and 5,019 SSR motifs (16.95%) were classified as compound repeats. Of the detected SSR motifs, the mono-nucleotide motif (240.30/Mb) was the most abundant in density, followed by di- (82.00/Mb), compound (69.10/Mb), tri- (48.66/Mb), tetra- (5.40/Mb), penta- (0.87/Mb), and hexa- (0.45/Mb) nucleotide motifs (**Figure 6C**).

### Fusion Transcripts and Transcription Factors (TFs)

Fusion transcripts, such as chimeric mRNA transcripts, result from either trans-splicing of distinct genes or aberrant chromosomal translocations (Wang et al., 2016). In our Iso-Seq dataset, a total of 365 fusion transcripts were identified, and their chromosome distribution was shown in **Figure 2H**. Among them, 43 fusion transcripts were observed in the intra-chromosomic region, while the others (322) were in the inter-chromosomic region. The results of GO enrichment analysis showed that the fusion transcripts were primarily (top six) associated with cell (GO:0005623), cell part (GO: 0044464), catalytic activity (GO:0003824), binding (GO: 0005488), cellular process (GO: 0009987), and single-organism process (GO: 0044699) (**Supplementary Figure 6**). Two fusion transcripts (PB.19, PB.130) were randomly selected and verified by RT-PCR (**Supplementary Figure 3C**).

In our Iso-Seq, a total of 1,194 TFs transcripts generated from 723 TFs genes were identified and their detailed information was shown in **Supplementary Table 4**. Based on the Animal TFDB 3.0 database classification, these TFs belong to more than 52 families. It is the first time to extensively identified TFs using transcriptome dataset in *L. maculatus*, which provided a useful foundation for TFs studies in the future.

### Differentially Expressed Transcripts (DETs) in Response to FW and SW Environment

To capture transcript-level expression differences in response to different salinity environment, the Illumina RNA-Seq data of gill tissue was aligned to the refined genome combined with both the reference genome and the Iso-Seq database for quantification. In total, 265.90 million clean reads were mapped to the new database. Using these criteria, a total of 518 DETs covering 497 gene loci were identified, of which 264 transcripts were up-regulated and 254 transcripts were down-regulated in the SW relative to the FW group (**Supplementary Table 5**). The distribution of DETs was illustrated in **Supplementary Figure 7**.

These candidate DETs were classified into eight functional groups, including energy metabolism, immune response, molecule and ion transport and metabolism, protein biosynthesis, protein degradation, RNA processing and modification, signal transduction, and structure reorganization based on the combination of GO and KEGG annotation, enrichment analysis, and published literature (**Supplementary Table 6**). These results indicated that transcripts showed different expression patterns in response to FW and SW environment. As shown in **Supplementary Figure 8**, among these DETs, exon skipping events were the most frequent AS type, accounting for 35.37% (208), followed by intron retention (32.48%, 191), which was different from their percentages in the Iso-Seq results.

**Figure 7:**
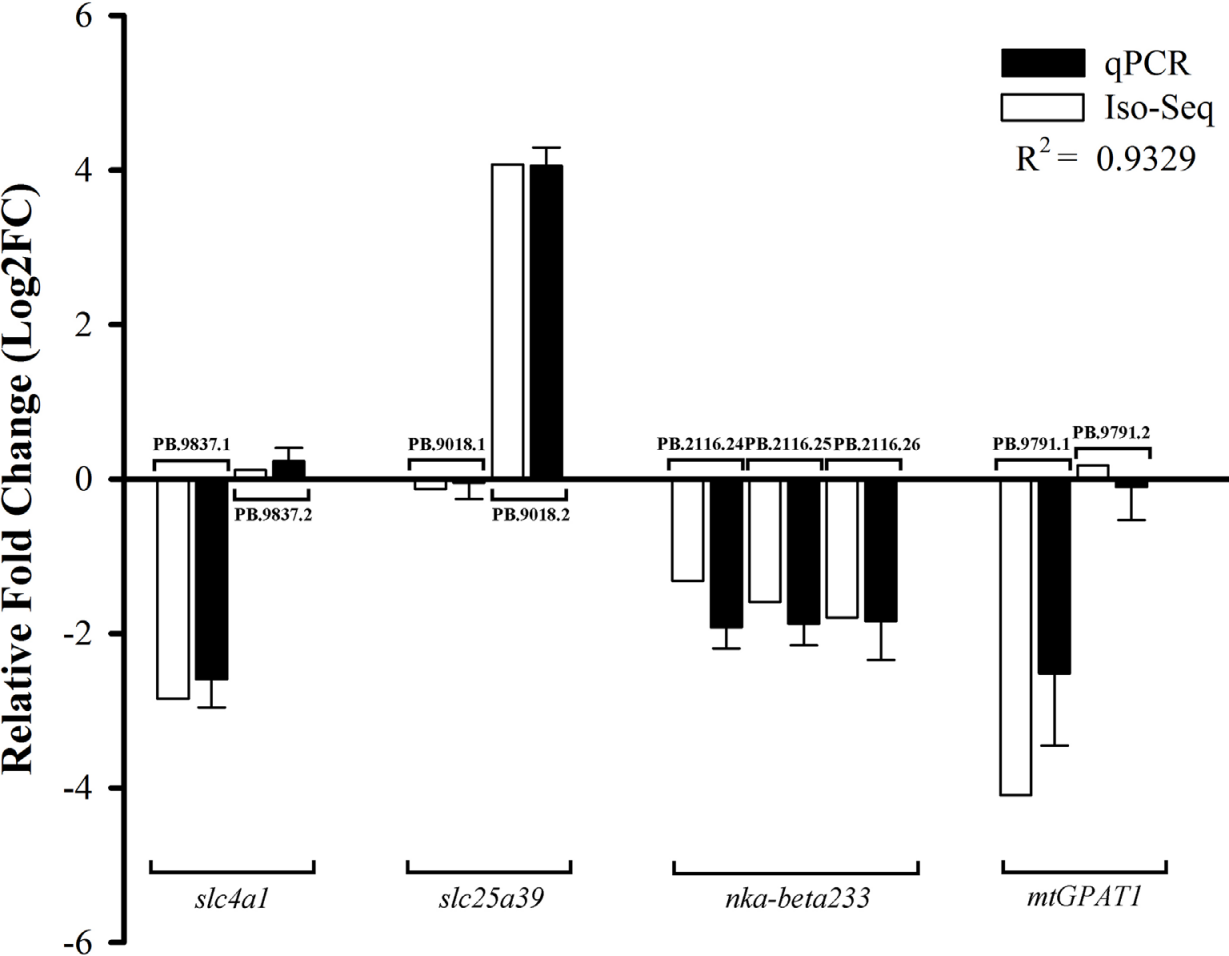
qPCR validation of DETs. The expression levels were showed as the log_2_ fold change in SW relative to FW group. Gene abbreviations were as followed: *slc4a1*, *solute carrier family 4a1*; *slc25a39*, *solute carrier family 25 member 39*; *nka-beta233*, *sodium/potassium-transporting ATPase subunit beta-233*; *mtGPAT1*, *glycerol-3-phosphate acyltransferase 1, mitochondrial*.

To verify the accuracy of the expression patterns of DETs by Iso-Seq, we randomly selected nine transcripts derived from four genes for qPCR validation (**Figure 7**). The experimental results demonstrated that the expression patterns were consistent with our analysis results, confirming the credibility and accuracy of DETs results.

### Genes With Distinct Differentially Expressed Transcripts (DETs)

In our study, a total of 518 DETs were generated from 497 genes, suggesting that some genes could produce at least two DETs. A total of 17 genes were found to generate several spliced variants, of which two or more variants were differentially expressed. Based on their expression patterns, two situations were observed for the genes (**Table 2**). Firstly, DETs from the same gene loci exhibited the similar expression trends. For example, all three transcripts of *Cysteine dioxygenase type 1* (*cdo1*) gene were significantly down-regulated in the SW relative to the FW group, and a similar expression pattern was also found in different transcripts of *sodium/potassium-transporting ATPase subunit beta-233* (*nka-beta233*) gene. The second situation was that DETs of the same gene exhibited the opposite expression trends (including seven genes; the schematic diagram of their structures was illustrated in **Supplementary Figure 9**). For example, *2,4-dienoyl-CoA reductase, mitochondrial* (*decr1*), involved in the decomposition of polyunsaturated fatty acids, was alternatively spliced to generate two transcripts with opposite expression patterns. Similar events were also discovered in *6-phosphofructo-2-kinase fructose-2,6-bisphosphatase 3* (*pfkfb3*), and *prostaglandin D2 receptor 2* (*ptgdr2*). The results suggested that spliced transcripts of the same gene may be involved in diverse physiological functions.

**Table 2:** List of genes with distinct differentially expressed transcripts (DETs) in SW relative to FW environment.

Gene name	Gene ID	Transcripts ID	FDR	Log_2_FC
Genes with distinct DETs in similar expression patterns				
Pre-mRNA-processing factor 39	evm.TU.scaffold_1.98	PB.341.2	0.000106	2.67500760
		PB.341.5	0.039993	1.53015860
Sodium/potassium-transporting ATPase subunit beta-233	evm.TU.scaffold_125.24	PB.2116.24	5.48E-05	−1.31694649
		PB.2116.25	0.04437353	−1.59047126
		PB.2116.26	1.22E-05	−1.79317407
Immunoglobulin heavy chain	evm.TU.scaffold_156.67	PB.3643.213	0.010499	−3.25928268
		PB.3643.274	0.031384	−1.20801476
Endothelial PAS domain-containing protein 1	evm.TU.scaffold_166.2	PB.3978.4	2.99E-06	1.23813931
		PB.3979.2	0.001415	2.01478964
Cuticle protein	evm.TU.scaffold_180.61	PB.4536.1	0.007865	1.03098816
		PB.4536.2	0.014347	6.48597657
		PB.4536.5	0.03675	1.21411681
		PB.4536.13	7.81E-05	1.19817302
Cytidine deaminase	evm.TU.scaffold_226.10	PB.6084.1	0.002701	−2.9710025
		PB.6084.2	4.35E-12	−2.76291983
Cysteine dioxygenase type 1	evm.TU.scaffold_3.79	PB.8015.1	1.42E-15	−6.26435354
		PB.8015.2	2.13E-11	−5.18024918
		PB.8015.3	6.58E-23	−5.03618275
Coiled-coil domain-containing protein 22	evm.TU.scaffold_309.8	PB.8408.5	0.000442	-inf
		PB.8408.6	0.002339	−2.24439212
Nucleolin 2	evm.TU.scaffold_41.58	PB.10153.4	0.000822	−1.82073360
		PB.10153.6	1.61E-05	-inf
Interferon-induced very large GTPase 1	evm.TU.scaffold_56.81	PB.11500.1	1.25E-24	−3.27867754
		PB.11500.2	1.16E-17	−2.07765032
Genes with distinct DETs in opposite expression patterns				
KAT8 regulatory NSL complex subunit 3	evm.TU.scaffold_0.458	PB.253.1	0.009568	−3.28744420
		PB.253.8	0.001073	3.68324251
Ran GTPase-activating protein 1	evm.TU.scaffold_116.12	PB.1622.1	0.046889	1.24749357
		PB.1622.5		−1.56839702
6-Phosphofructo-2-kinase/fructose-2,6-bisphosphatase 3	evm.TU.scaffold_118.17	PB.1707.1	6.17E-29	inf
		PB.1707.4	2.61E-23	−5.16585478
Prostaglandin D2 receptor 2	evm.TU.scaffold_29.136	PB.7861.1	0.026324	−3.1457994
		PB.7861.2	0.045375	2.30238325
2,4-Dienoyl-CoA reductase, mitochondrial	evm.TU.scaffold_313.25	PB.8538.1	2.65E-06	−2.72486991
		PB.8538.2	0.025641	1.04664231
Protein kinase C-binding protein 1	evm.TU.scaffold_44.98	PB.10442.1	0.010769	−2.17623890
		PB.10442.2	1.13E-07	inf
MAPK/MAK/MRK overlapping kinase	evm.TU.scaffold_80.12	PB.13693.1	0.039827	−3.55865208
		PB.13693.2	7.81E-05	inf

### Differentially Expressed Transcription Factors

Of 518 DETs, 17 DETs were identified as TFs belonging to 8 families, including C2H2-ZF, bHLH, ETS, Fork head, HMG, Homeobox, MBD, and RHD (**Supplementary Table 7**). Both C2H2-ZF and bHLH TF families have been well characterized with roles in response to stresses (Fujita et al., 2006; Terova et al., 2008; Steinberg, 2012). Of 17 differentially expressed TFs, 14 TFs were found to be up-regulated in SW relative to FW group, suggesting that TFs may play important roles in response to hypertonic stress and enhance salt tolerance of *L. maculatus*.

## Discussion

In our study, we employed PacBio Iso-Seq to uncover the complexity of the *L. maculatus* transcriptome, providing the first comprehensive view of splice variants in aquaculture teleosts. Using Iso-Seq, 2,580 novel genes were discovered, accounting for more than 10% of the total number of genes in the *L. maculatus* genome. The most impressive gene was *immunoglobulin heavy chain* with 279 unique transcripts, which was even more than *neurexin-1-alpha* with 247 splicing variants in mouse (Barbara et al., 2014). This suggests that Iso-Seq is advantageous for the identification of novel gene loci and the detection of alternative transcripts, which is consistent with a previous study. In addition, post-transcriptional events (AS and APA), lncRNAs, and fusion transcripts were predicted to improve the understanding of the complexity of *L. maculatus*. These results would be great resource for further analysis of post-transcriptional events and refinement of the annotation of the *L. maculatus* reference genome.

Over the past decade, it has been shown that AS is a major mechanism for the enhancement of transcriptome and proteome diversity (Keren et al., 2010). A certain AS event is the outcome of the cooperative or antagonistic interactions between RNA *cis*-elements and splicing factors (Black, 2003; Matlin et al., 2005), including members of the serine-arginine-rich protein family (Fu, 1995), members of the heterogenous nuclear ribonucleoproteins family (Krecic and Swanson, 1999), and other specific proteins (Underwood et al., 2005). Accumulating evidence indicates that numerous stimuli, such as growth factors, cytokines, and stress, would alter the choice of splice sites and produce multiple transcripts (Barbazuk et al., 2008). Multiple transcripts in teleosts could promote the tolerance to stresses (Xia et al., 2017; Tan et al., 2018). In our study, several typical splicing factors, including *serine/arginine-rich splicing factor 1*, *serine/arginine-rich splicing factor 7*, *RNA-binding protein 5*, *RNA-binding protein 33*, *RNA-binding protein 39,* and *RNA-binding protein 47*, were differentially expressed under salinity challenge, indicating the splice factors and AS events in *L. maculatus* can be activated by salinity stimuli. The stress-induced AS events could increase the tolerance to the stresses by two different mechanisms. 1) stress-induced AS events could generate aberrant transcripts with splicing errors, which would be removed by nonsense mediated mRNA decay (Wollerton et al., 2004; Chang et al., 2007). This mechanism could weaken the function of the corresponding genes by decreasing the abundance of functional transcripts (Maquat, 2004; Chang et al., 2014; Cui et al., 2014). 2) AS transcripts could encode unique protein, often with alternations in localization, activity, and function (Wang et al., 2008; Kalam et al., 2017). Moreover, their biological function change and expression abundance regulation are largely independent process to increase organismal tolerance against stresses. For example, in human, Na^+^/K^+^/2Cl^-^ cotransporter (*nkcc2*) is proved to be key regulator associated with salt and water homoeostasis in kidney. At least 3 *nkcc2* transcripts are generated *via* different splicing of exon 4 (Schiessl and Castrop, 2015). The exon 4 encodes the second transmembrane domain, which is crucially involved in the Cl^-^ binding (Haas and Mcmanus, 1983; Schiessl and Castrop, 2015). As a result, these *nkcc2* transcripts differ markedly in their ion affinities and transport characteristics (Haas and Mcmanus, 1983; Schiessl and Castrop, 2015). The *nkcc2* splicing is need for enhanced ion reabsorption during a salt-restricted diet, even without changes in total *nkcc2* abundance (Schiessl and Castrop, 2015). In our study, three *nkcc2* transcripts were also identified in *L. maculatus* and their splicing mode has been shown in **Supplementary Figure 5A**. These transcripts in *L. maculatus* were generated by different splicing mode similar with those in human, which 5 exons were lost between exon 2 and 8; intron is retained between exon 23 and 24. The various splicing modes may also change transmembrane domain of *nkcc2* and influence their ion affinities. However, their specific physiological function is required to be further studied in the future. Besides, AS transcripts could also regulate their expression abundance to cope with stress, which has been widely reported in previous transcriptomic studies (Xia et al., 2017). In our study, a total 518 DETs were identified in response to different salinity environment. However, the functional significance of most spliced transcripts in teleosts is yet to be fully elucidated. Hence, their gene function would be discussed as follows.

In response to cell shrinkage and swelling caused by salinity stress, fishes need to cope with salt depletion or gain, and water loss or gain. In the molecule and ion transport and metabolism groups, the expression of *sodium/potassium-transporting ATPase subunit alpha-2* and *beta-233* transcripts were significantly up-regulated in the FW environment. Both the *sodium/potassium-transporting ATPase subunit alpha-2* and *beta-233* genes are members of the sodium/potassium-transporting ATPase family, which play an important role in providing a driving force for ion transport to maintain cell osmotic balance and volume in euryhaline teleosts, such as senegal sole (*Solea senegalensis*)(Skou and Esmann, 1992; Feng et al., 2002; Armesto et al., 2014). *Solute carrier family 4 a1* (*slc4a1*) is generally accepted as a bifunctional protein with both Cl^-^/HCO3^-^ exchange and Cl^-^/taurine channel functions (Romero et al., 2013). It has been proposed that hypotonic stress induces taurine movements *via* an anion channel that is depending on or controlled by *slc4a1* (Fiévet et al., 1995). Consistent with previous studies, our data indicated that the expression of *slc4a1* in the FW environment was up-regulated in response to swelling stress. In coping with hypertonic stress, *adenosylhomocysteinase 2* gene could reduce the apparent affinity for intracellular Mg^2+^ in the inhibition of *slc4a1* currents, which explains the high expression level of *adenosylhomocysteinase 2* in the SW environment (Soichiro and Toru, 2014).

An adequate and timely energy supply is a prerequisite for enzymes and transporters used in iono- and osmoregulatory processes (Tseng and Hwang, 2008). The oxidation of glucose and fatty acids is the major source of energy for organisms (Lavrentyev et al., 2004). In the energy metabolism group, transcripts of *6-phosphofructo-2-kinase/fructose-2,6-bisphosphatase 3* and *2,4-dienoyl-CoA reductase*were differentially expressed in response to salinity stress. *6-phosphofructo-2-kinase/fructose-2,6-bisphosphatase 3* plays a role in maintaining elevating *fructose-2,6-bisphosphate* levels, which is considered as the major regulator controlling carbon flux through glycolysis (Sakakibara et al., 1997; Chesney et al., 1999; Alexander et al., 2002; Kawaguchi et al., 2015). *2,4-dienoyl-CoA reductase* encodes an essential enzyme that participates in the beta-oxidation and metabolism of polyunsaturated fatty enoyl-CoA esters (Gurvitz et al., 1999).

Adaptive and acclamatory responses of fish to salinity stress depend on efficient mechanisms of osmosensing and osmotic stress signaling (Kultz, 2015). Instead of directly coupling osmosensors to osmotic effector proteins, large-scale osmoregulatory mechanisms are operated by linking molecular osmosensors to cell signaling pathways to initiate adaptive reactions (Evans, 2010). In our study, several DETs were involved in typical signal transduction, such as *mitogen-activated protein kinase kinase kinase 14*, *tyrosine-protein kinase Fyn-like*, *rho GTPase-activating protein 35*, *tyrosine kinase 2*, and *serine/threonine-protein kinase Sgk2*. They may integrate and amplify signals from osmosensors to activate appropriate downstream targets mediating physiological acclimation (Kültz, 2010; Zhang et al., 2017).

In addition, one of the *heat shock 70 kDa protein* transcripts was differentially expressed in the SW group. *Heat shock 70 kDa protein*, known as chaperone proteins, is pivotal in maintaining protein homeostasis by interacting with stress-denatured proteins to prevent their aggregation and malfolding (Parsell and Lindquist, 1993). In the protein degradation classification, many DETs were involved in ubiquitin. Ubiquitin in cells acts as a covalent modifier of proteins in functionalization and degradation, which is dependent on ubiquitin ligase (Lyu et al., 2018). E3 ubiquitin proteins are the final enzymes in the ubiquitin-proteasome pathway, regulating protein degradation, cell growth, and apoptosis in response to environmental changes (Mani and Gelmann, 2005; Sardella and Kultz, 2009; Li et al., 2014).

Cytoskeletal organization is notably affected by perturbations in cell volume. Thus, cytoskeletal protein has been considered as a putative osmosensor. Correspondingly, several DETs are found to be involved in structural components of the cytoskeleton, such as *cuticle protein*, *filamin-B*, and *beta tubulin.* In addition, previous reports demonstrate that salinity could enhance the abundance of innate immune defenses proteins, and chronic salinity stressors could stimulate the proliferation and antimicrobial functions of innate immune cells, as well as the release of pro-inflammatory cytokines, in several euryhaline and stenohaline fish species (Cuesta et al., 2005; Delamare-Deboutteville et al., 2006; Jiang et al., 2008; Schmitz et al., 2016). In *L. maculatus*, several transcripts, encoding immune-related proteins, also exhibited differentially expressed profiles, such as *classical MHC class I molecule alpha-chain*, *tumor necrosis factor receptor superfamily member 6B*, *IgGFc-binding protein-like*, and *leucine-rich-repeat-containing protein C3*.

Recently, accumulating evidence indicates that TFs are also crucial in mediating organism adaptation to salinity stresses by activating or suppressing downstream genes in the pathway (Fujita et al., 2006; Nie et al., 2019). Indeed, TFs are also greatly affected by AS events and TF transcripts with the alternative function often are low in abundance. One interesting example is the sex determination mechanism of fruit fly. *Sex-lethal*, acting as a master regulatory switch in female flies, plays a key role in orchestrating the changes in gene expression responsible for all aspects of sexual determination in fruit fly (Förch and Valcárcel, 2003). Despite the presence of *Sex-lethal* transcripts in both sexes, however, *Sex-lethal* protein is only produced in female flies. It is resulted from a critical difference between the transcripts in the two sexes: exon 3 with stop codons in frame is included in male flies and skipped in females (Salz et al., 1989; Bell et al., 1991). However, little is known about similar mechanism in response to salinity. In our study, a total of 1,194 TF transcripts from 723 genes are identified in *L. maculatus*, suggesting AS events are common in the TF genes of *L. maculatus*. The question remains as to whether there exists alternative biological function among TF transcripts. A satisfactory answer to this question will require further researches in the future. Additionally, a total of 17 TF transcripts were differentially expressed after salinity change, including members of C2H2-ZF, bHLH, ETS, and others. Previous studies have demonstrated that C2H2-ZF (Steinberg, 2012), bHLH (Terova et al., 2008; Liu et al., 2009), Homeobox (Nie et al., 2019), RHD (Carlsen et al., 2004), and ETS (Wasylyk et al., 1998) TFs could be crucial in increasing stresses tolerance by signal transduction or modulation pathway. MBD TFs are mainly involved in the cytosine methylation of the nuclear DNA (Nan et al., 1998) and HMG proteins are ubiquitous nuclear proteins that bind to DNA, nucleosomes and induce structural changes in the chromatin fiber (Hock et al., 2007). In our study, a total of 17 differentially expressed TFs suggested their important roles in response in salinity change in *L. maculatus*.

Post-transcriptional regulatory mechanisms, including AS, APA, and fusion transcripts, make essential contribution to physiological function regulatory of aquaculture species. For example, AS events have been studies in Pacific oyster (*Crassostrea gigas*) (Huang et al., 2016), Nile tilapia (Xia et al., 2017) and channel catfish (*Ietalurus punetaus*) (Tan et al., 2018) using Illumina RNA-Seq datasets. However, these RNA-Seq projects of aquaculture species obtain transcripts only based on short read-based assembly, which would limit the accuracy of identification of post-transcriptional events. In our study, it is the first time that Iso-Seq is applied in the aquaculture teleost, which has detected numerous full-length transcripts and characterized many post-transcriptional regulatory events in *L. maculatus*. It creates a paradigm for future post-transcriptional regulatory studies of aquaculture species in transcriptome wide. Besides, this study investigates the DETs of euryhaline *L. maculatus* in response to different salinity environment. It has pushed the limit of previous gene-level transcriptome studies (Zhang et al., 2017), which would be helpful to unveil molecular mechanism of coping with salinity stress in fishes.

## Conclusion

In our study, we applied PacBio Iso-Seq to yield a new set of transcriptomic data of *L. maculatus* as follows: 28,809 non-redundant transcripts, 10,249 AS events, 1,359 APA events, 3,112 lncRNA, 29,609 SSRs, 365 fusion transcripts, and 1,194 TFs. It is the first time in aquaculture teleosts that Iso-Seq was applied to unveil the transcriptome complexity. To investigate transcripts involved in salinity regulation in *L. maculatus*, RNA-Seq data was combined with Iso-Seq results and identified 518 DETs in different environment. Notably, transcripts from the same genes may exhibit similar or opposite expression patterns. In addition, the expression level of 14 TFs is significantly up-regulated in SW environment, implying their roles in hypertonic stress. Our study not only improves current gene models of *L. maculatus*, but also enhances the understanding of salinity regulatory mechanisms in euryhaline teleosts.

## Data Availability Statement

The raw sequences of our study have been submitted to the Sequence Read Archive (SRA) of National Center for Biotechnology Information (NCBI) with the accession number of PRJNA515783 (BioProject ID of Iso-Seq) and PRJNA515986 (BioProject ID of RNA-Seq). Reference genome of *L. maculatus* was downloaded from NCBI with the accession number of PRJNA407434 (BioProject ID).

## Ethics Statement

This study was carried out in accordance with the recommendations of “Animal Research and Ethics Committees of Ocean University of China (Permit Number: 20141201).” The protocol was approved by the “Animal Research and Ethics Committees of Ocean University of China.”

## Author Contributions

YL and YT conceived the study. YT, XQ, XZ, and SL performed bioinformatics analysis. YL provided funding support. WY collected samples and extracted RNA samples. HW, JL, and FH administrated the project. BL and YS verified the sequencing results. All authors read and approved the final manuscript.

## Funding

This study was supported by National Natural Science Foundation of China (No.31602147), National Key R&D Program of China (No.2018YFD0900101), and China Agriculture Research System (No. CARS-47).

## Conflict of Interest

The authors declare that the research was conducted in the absence of any commercial or financial relationships that could be construed as a potential conflict of interest.

## Supplementary Material

Supplementary Figure 1 ROIs length distribution of three size bins.

Supplementary Figure 2 Examples of structures showing the gene with different types of isoforms. The blue bars indicated the annotated gene model in the L. maculatus genome. In **(A)**, the orange bars indicated the isoform structure detected by Iso-Seq. Lines represented introns, and arrows indicated the orientation of transcription. In **(B)**, novel gene and their transcripts were absent in the annotation of the genome.

Supplementary Figure 3 Examples of validation experiments for **(A)** novel isoforms, **(B)** APA event and **(C)** fusion transcripts.

Supplementary Figure 4 KEGG analysis of the genes with more than four isoforms. **(A)** KEGG pathway annotation; **(B)** Statistics of pathway enrichment.

Supplementary Figure 5 Examples of five types of alternative splicing events detected by Iso-Seq. The blue bars indicated the annotated gene model in the *L. maculatus* genome, and dark green bars indicated the transcripts structure detected by Iso-Seq. **(A)** Example of intron retention events in evm.TU.scaffold_294.3; **(B)** Example of exon skipping events in evm.TU.scaffold_229.19; **(C)** Example of alternative 3’ splice site events in evm.TU.scaffold_13.295; **(E)** Example of alternative 5’ splice site events in evm.TU.scaffold_81.120; **(E)** Example of mutually exclusive exon events in evm.TU.scaffold_98.14;

Supplementary Figure 6 Histogram of gene ontology classifications of *L. maculatus* fusion transcripts.

Supplementary Figure 7 Volcano plot showing the DETs between the FW and SW treatment groups. The horizontal axis was the log2 fold change in SW relative to FW groups. The vertical axis was the -log10 false discovery rate. Green dots represented significantly down-regulated transcripts in SW relative to FW group, while red dots represented significantly up-regulated transcripts. Black dots represented transcripts without significant expression difference between two groups.

Supplementary Figure 8 Pie chart showing frequencies of five types of alternative spliced events in DETs.

Supplementary Figure 9 The transcripts structures of DETs with opposite expression patterns generated from 7 genes. Blue bars indicated the annotated gene model in the *L. maculatus* genome, and orange bars indicated the transcripts detected by Iso-Seq. Transcripts with DETs were marked with red dashed rectangles.
