# Supplementary figures and images for: Characterization of Full-Length Transcriptome Sequences and Splice Variants of Lateolabrax maculatus by Single-Molecule Long-Read Sequencing and Their Involvement in Salinity Regulation
Source: Front Genet. 2019 Nov 15;10:1126. doi: 10.3389/fgene.2019.01126 (PMC6873903; doi:10.3389/fgene.2019.01126)

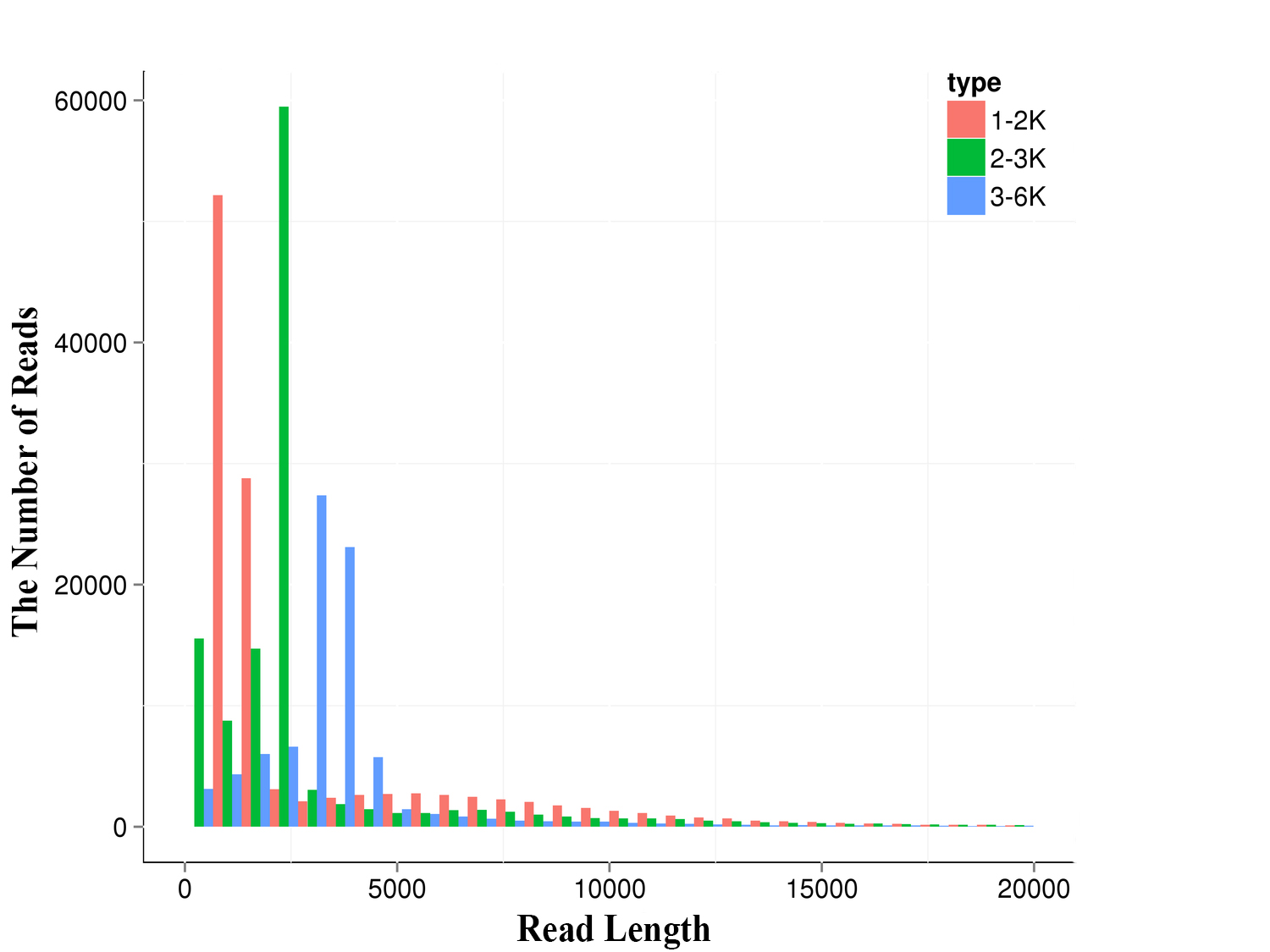

Supplement: Supplementary Figure 1 — ROIs length distribution of three size bins. [file Image_1.jpeg]

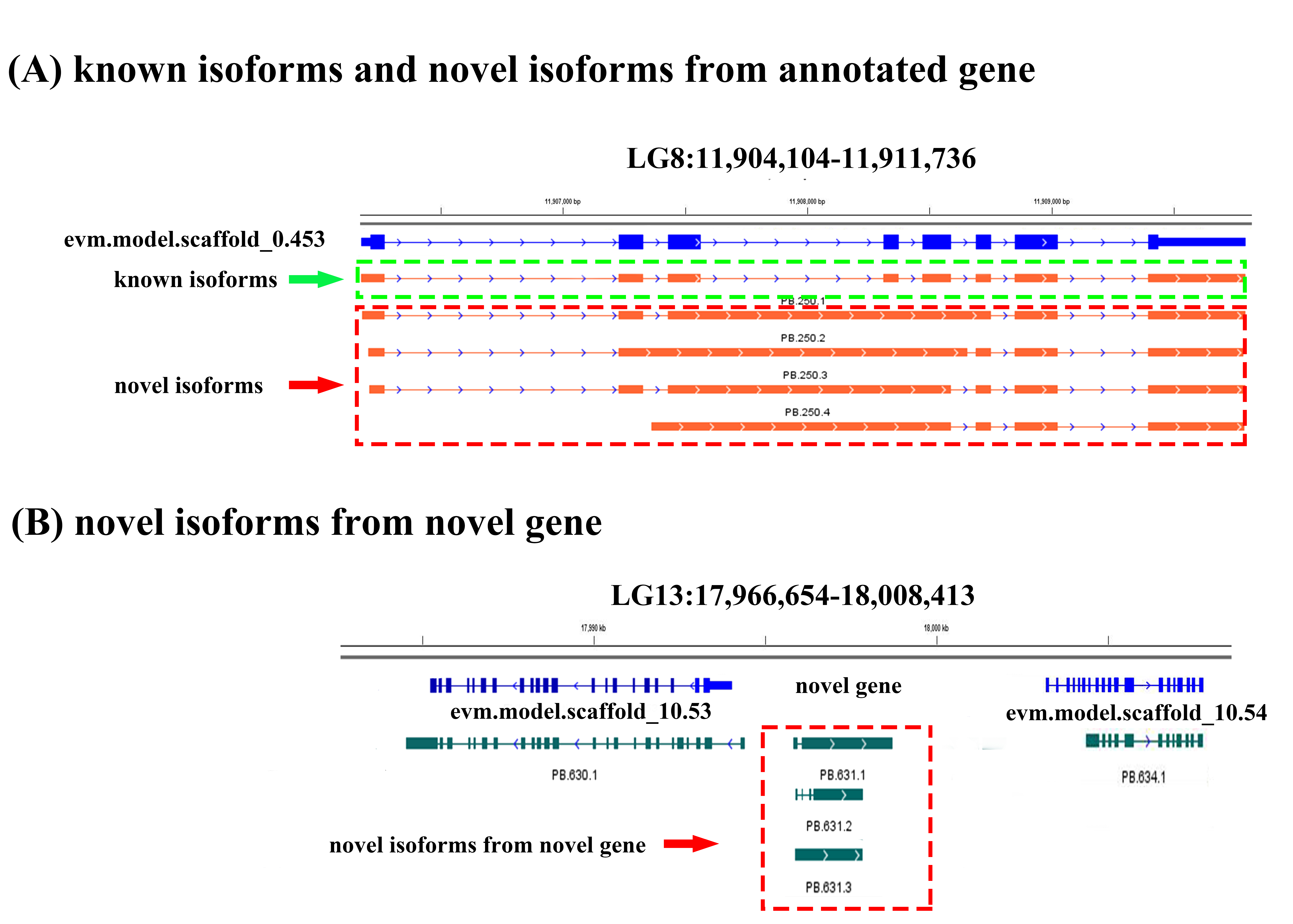

Supplement: Supplementary Figure 2 — Examples of structures showing the gene with different types of isoforms. The blue bars indicated the annotated gene model in the L. maculatus genome. In (A), the orange bars indicated the isoform structure detected by Iso-Seq. Lines represented introns, and arrows indicated the orientation of transcription. In (B), novel gene and their transcripts were absent in the annotation of the genome. [file Image_2.jpeg]

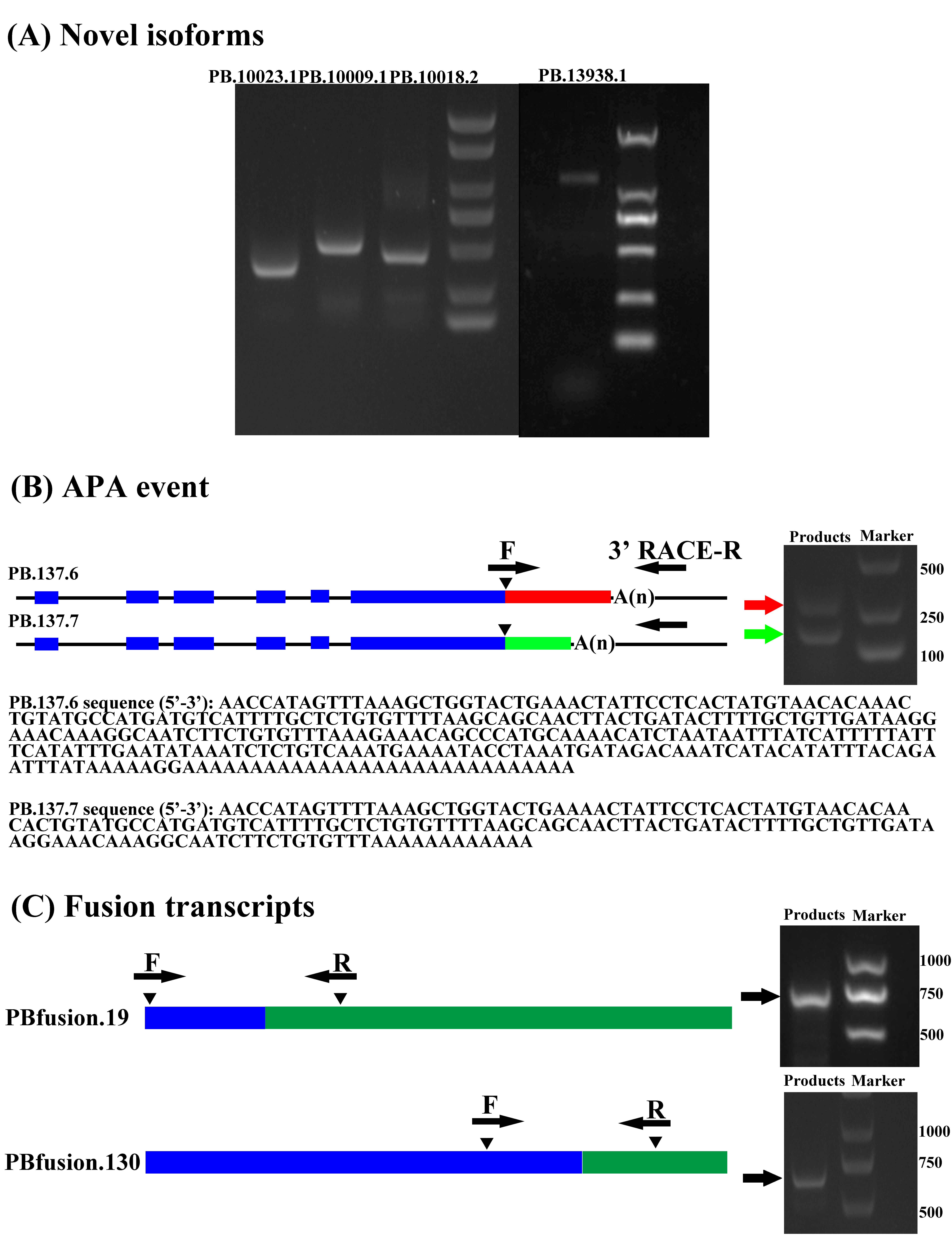

Supplement: Supplementary Figure 3 — Examples of validation experiments for (A) novel isoforms, (B) APA event and (C) fusion transcripts. [file Image_3.jpeg]

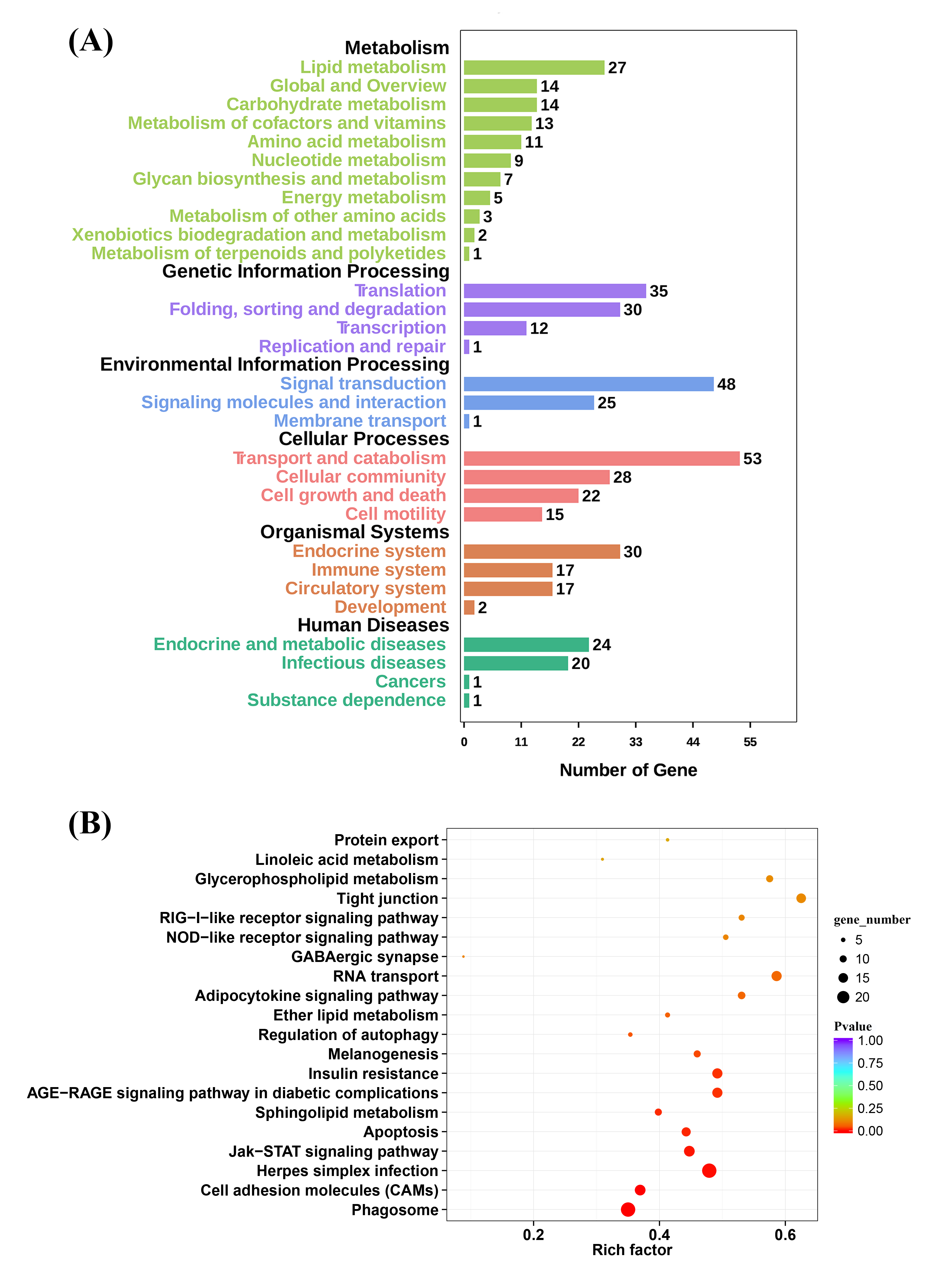

Supplement: Supplementary Figure 4 — KEGG analysis of the genes with more than four isoforms. (A) KEGG pathway annotation; (B) Statistics of pathway enrichment. [file Image_4.jpeg]

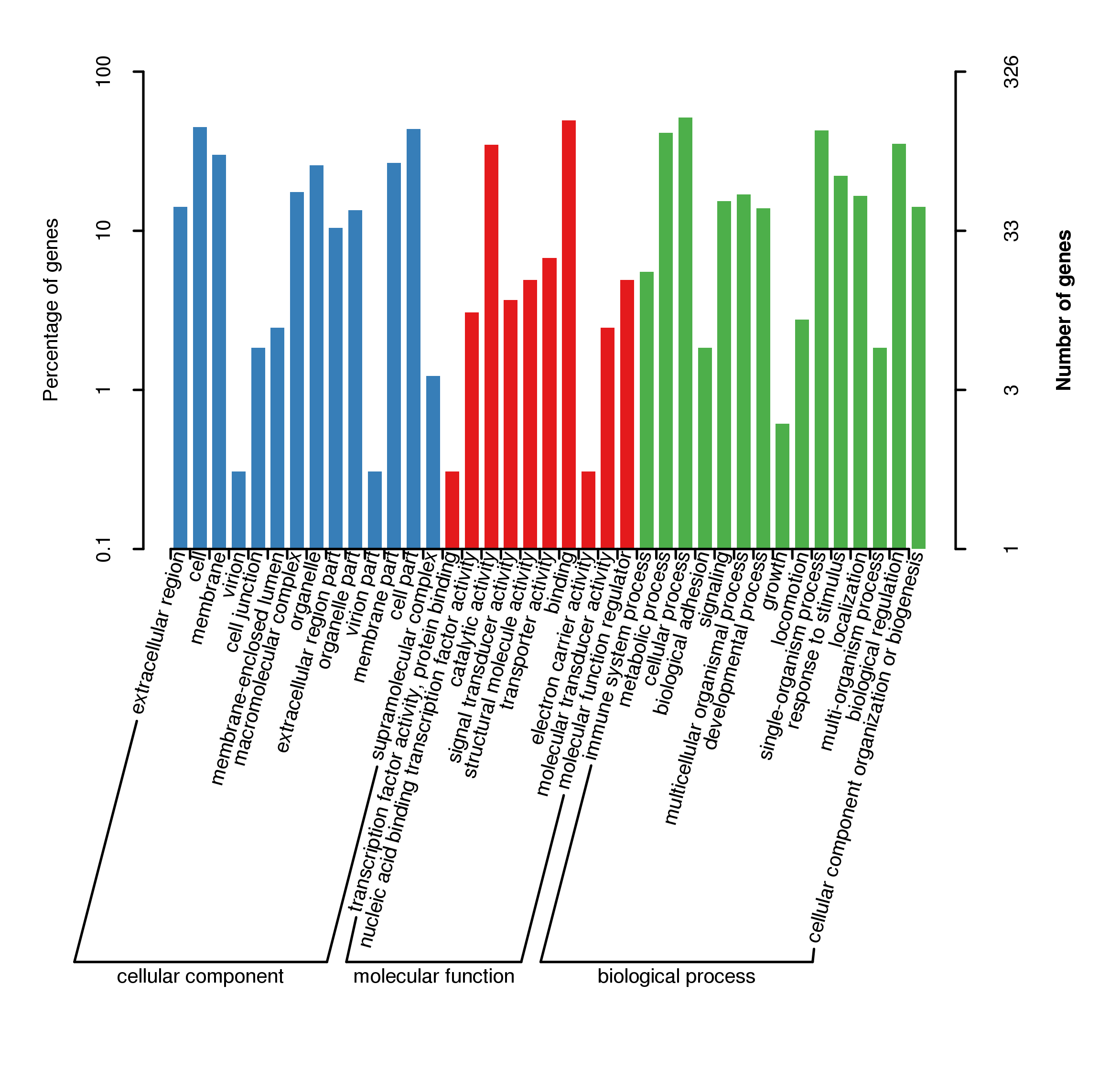

Supplement: Supplementary Figure 6 — Histogram of gene ontology classifications of L. maculatus fusion transcripts. [file Image_6.png]

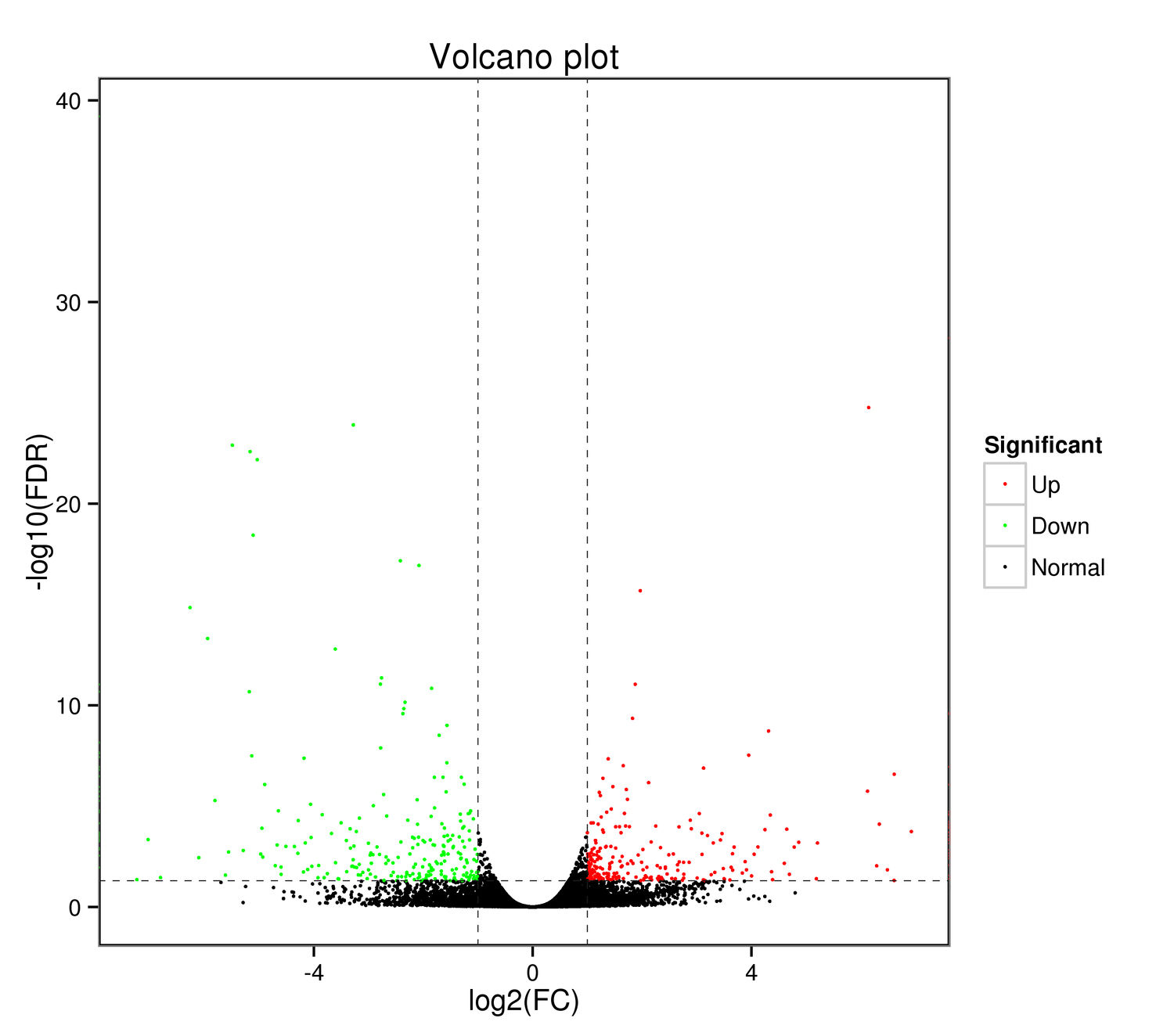

Supplement: Supplementary Figure 7 — Volcano plot showing the DETs between the FW and SW treatment groups. The horizontal axis was the log2 fold change in SW relative to FW groups. The vertical axis was the -log10 false discovery rate. Green dots represented significantly down-regulated transcripts in SW relative to FW group, while red dots represented significantly up-regulated transcripts. Black dots represented transcripts without significant expression difference between two groups. [file Image_7.jpeg]

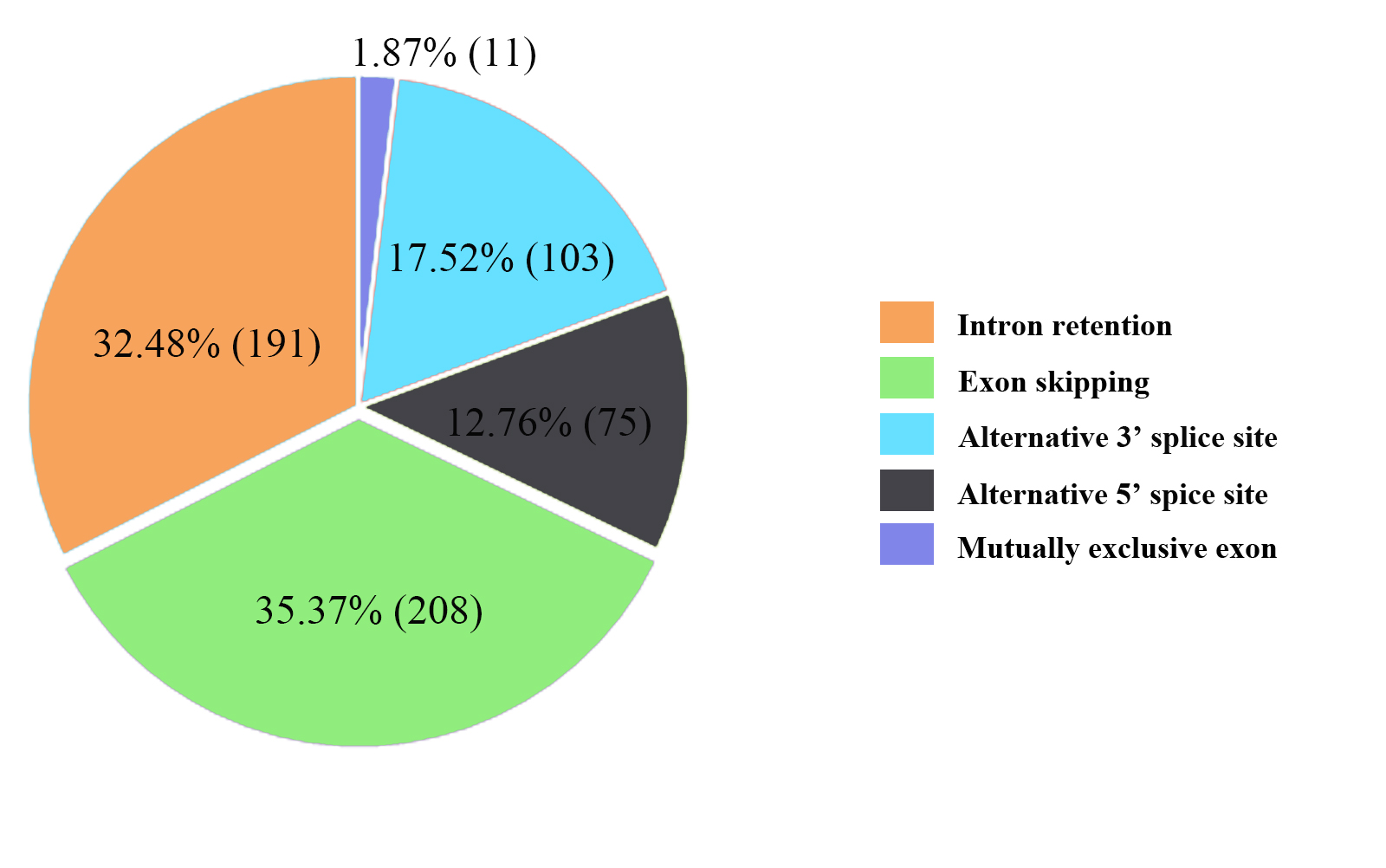

Supplement: Supplementary Figure 8 — Pie chart showing frequencies of five types of alternative spliced events in DETs. [file Image_8.jpeg]

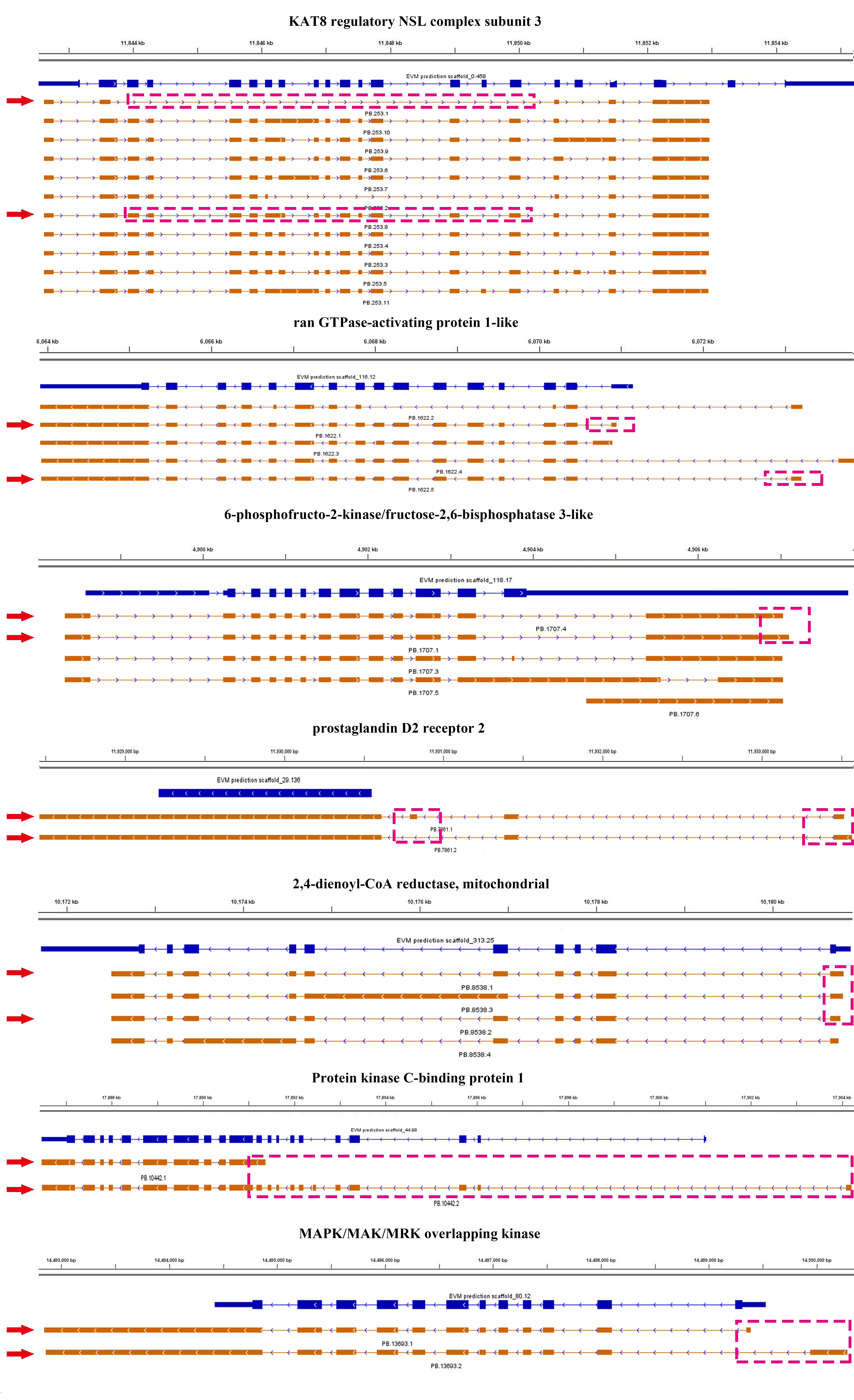

Supplement: Supplementary Figure 9 — The transcripts structures of DETs with opposite expression patterns generated from 7 genes. Blue bars indicated the annotated gene model in the L. maculatus genome, and orange bars indicated the transcripts detected by Iso-Seq. Transcripts with DETs were marked with red dashed rectangles. [file Image_9.jpeg]
