# Supplementary material for: Characterization of Full-Length Transcriptome Sequences and Splice Variants of Lateolabrax maculatus by Single-Molecule Long-Read Sequencing and Their Involvement in Salinity Regulation
Source: Front Genet. 2019 Nov 15;10:1126. doi: 10.3389/fgene.2019.01126 (PMC6873903; doi:10.3389/fgene.2019.01126)
Supplement: Supplementary file 10 [file DataSheet_1.docx]

**Supplementary Tables:**

**Supplementary Table 1 Primers of RT-PCR designed for alternative splicing event, novel isoforms and fusion transcripts and APA event validation.**

| Gene Name | Gene loci | Primer (5’-3’) |  |
| --- | --- | --- | --- |
| Epsin-1 | evm.TU.scaffold_24.26 | F: TCCAGCAGAGTAAACAACG  R: TCGGGAGTCTTACGAACA | |
| Serine/threonine-protein kinase 19 | evm.TU.scaffold_40.228 | F: ATGAACAGGAAACGGGCTCT  R: CCTCGCTCCCGCAGTTTA | |
| SLC52A2 | evm.TU.scaffold_41.76 | F: ATGTTTCGTTACCCTCCTC  R: CTCGTGAAGCAAAGTCCC | |
| Integrin-alpha | evm.TU.scaffold_4.261 | F: GAGGAGTTGTGATGGTGGCT  R: CCTCCTTCGCCGTGGTAT | |
| Ras-related protein Rab-18 | evm.TU.scaffold_110.37 | F: CTGACGACGCTGAAGATA  R: ACACTGAACGCCGTCCCT | |
| Glucocorticoid-induced transcript 1 protein | evm.TU.scaffold_14.111 | F: TCAGCCGACAGAGCAAAG  R: TGGAGGAGTAGGACGAGGG | |
| Dihydroxyacetone phosphate acyltransferase | evm.TU.scaffold_1.189 | F: GGGCTACACGCTCACCAA  R: ATTCCTCCCAGAAACTCG | |
| Carbonic anhydrase related protein | evm.TU.scaffold_315.16 | F: CGTCCGCATCATCCTCAA  R: ACCCAGATGCTCCTTACC | |
| Serine/threonine-protein kinase PLK2 | evm.TU.scaffold_3.159 | F: GGAGGCTTCGCCAAATGC  R: GAGTTTCGTAGTATTTGACCT | |
| Tricarboxylate transport protein | evm.TU.scaffold_3.22 | F: CAACCGTGCAAAGGACGAG  R: TTCAGGACCTTCACCACT | |
| Novel gene PB.13938.1 | PB.13938 | F: CAGCAGAAGGCTACCACT  R: TTCATCCAGGAGGGCAAG | |
| Novel gene PB.10009.1 | PB.10009 | F: GGAGCGAACTTCCTAACC  R: CCCTGTTTCTCGGCACTA | |
| Novel gene PB.10018.2 | PB.10018 | F: AGGGACGCTTACACCACA  R: GCTGATACAACACCTCCAC | |
| Novel gene PB.10023.1 | PB.10023 | F: AGGTGGCAAATAGAGTGA  R: ATGACGATAATGCTGGTG | |
| Fusion transcripts PB.19 | PBfusion.19 | F: CTGCTGAACTCGGCTCTA  R: GAAGAATAGGGCATAGGT | |
| Fusion transcripts PB.130 | PBfusion.130 | F: GTATGCGTCCTTCTAATCTG  R: CACCCTTTGTTCTTTGTTGC | |
| APA event | evm.TU.scaffold_0.266 | F:AACACCACCACCCAAAGACGCTATT | |

**Supplementary Table 2 Primers for qPCR validation**

| Gene name | Transcript ID | Primers (5’-3’) |
| --- | --- | --- |
| Band 3 anion exchange protein (*slc4a1*) | PB.9837.1 | F: GAGATGCGTTCATTCAGC  R: CTCGTGGTAGTTCATGTCAGTC |
|  | PB.9837.2 | F: CGCACATCTCCTACCTCA  R: AGACAAAGCGGCGAAGTA |
| Solute carrier family 25 member 39 (*cgi-69*) | PB.9018.1 | F: ACTGGAGCCCTCCTCACA  R: AACCCACTCCACAAAGAC |
|  | PB.9018.2 | F: GATTGCTGGGAAGGCTAG  R: AACCCACTCCACAAAGAC |
| Sodium/potassium-transporting ATPase subunit beta-233 (*beta-233*) | PB.2116.24 | F: AGACGATGGCGGCTGGAA  R: CATCTGGTCCCTCTGGTT |
|  | PB.2116.25 | F: CCACTGGTGCTCCTTTCT  R: CATCTGGTCCCTCTGGTT |
|  | PB.2116.26 | F: ACCTCCTTCACCATTTCC  R: ACATGCCATCTGTATCTG |
| Glycerol-3-phosphate acyltransferase 1 (*gpam*) | PB.9791.1 | F: CAAATCCCACATTGACTA  R: TGTCTTTCTTCCCATTCC |
|  | PB.9791.2 | F: CTCTGGTCGCCTTTAGTC  R: TTTGGGACAGATGAGATA |
| 18S | - | F: GGGTCCGAAGCGTTTACT  R: TCACCTCTAGCGGCACAA |

**Supplementary Table 4 Information of transcription factors identified by Iso-Seq.**

| Transcription factors family | Gene number | Transcript number |
| --- | --- | --- |
| C2HC-ZF | 178 | 243 |
| Misc | 95 | 143 |
| bZIP | 60 | 117 |
| ZBTB | 66 | 97 |
| bHLH | 41 | 89 |
| Homeobox | 47 | 66 |
| HMG | 28 | 54 |
| ETS | 23 | 48 |
| STAT | 8 | 37 |
| IRF | 10 | 32 |
| MYB | 13 | 20 |
| RHD | 7 | 18 |
| THAP | 15 | 18 |
| ZF-GATA | 10 | 18 |
| Fork head | 16 | 17 |
| CSD | 6 | 16 |
| MH1 | 9 | 16 |
| E2F | 9 | 12 |
| TSC22 | 6 | 12 |
| CP2 | 5 | 10 |
| ARID | 6 | 9 |
| MDB | 5 | 8 |
| TEA | 5 | 8 |
| LITAF | 5 | 8 |
| NF-YC | 3 | 7 |
| T-box | 3 | 6 |
| HSF | 2 | 5 |
| NF-YB | 4 | 5 |
| SRF | 3 | 5 |
| RFX | 2 | 4 |
| Runt | 3 | 4 |
| SAND | 3 | 4 |
| CSL | 1 | 3 |
| HMGI_HMGY | 2 | 3 |
| NCU-G1 | 1 | 3 |
| NF-YA | 1 | 3 |
| p53 | 2 | 3 |
| zf-BED | 2 | 3 |
| COE | 1 | 2 |
| HPD | 2 | 2 |
| PAX | 2 | 2 |
| TF_Otx | 2 | 2 |
| ZF-MIZ | 2 | 2 |
| ZF-NF-X1 | 1 | 2 |
| AP | 1 | 1 |
| CBF | 1 | 1 |
| CG-1 | 1 | 1 |
| CTF_NFI | 1 | 1 |
| GCM | 1 | 1 |
| HTH | 1 | 1 |
| PC4 | 1 | 1 |
| Tub | 1 | 1 |
| Others | 14 | 28 |
| Total | 723 | 1194 |

Abbreviations: C2H2-ZF, C2H2-type zinc finger; bZIP, basic leucine zipper domain; Misc, Miscellaneous; ZBTB, zinc finger and BTB domain; bHLH, basic helix-loop-helix; HMG, high mobility group; STAT, signal transducer and activator of transcription; IRF, interferon regulatory factor; RHD, Rel homology domain; THAP, thanatos-associated protein domain; ZF-GATA, GATA-type zinc finger; MH1, MH1 domain; TSC22, TGF-beta1 Stimulated Clone-22; ARID, AT-Rich Interaction Domain; MDB, Methyl-CpG-binding domain; TEA, TEA domain; LITAF, LPS-induced TNF alpha transcription factor; NF-YC, nuclear factor-Y. subunit C; SRF, serum response factor; HSF, heat stress factor; NF-YB, nuclear factor-Y. subunit B; RFX, tegulatory Factor X; Runt, runt-related transcription factor; SAND, sand domain transcription factor; CSL, Cell-fate specification; NF-YA, nuclear factor YA; zf-BED, zinc finger and BED domain; COE, Collier/Olf/EBF; PAX, paired-homeobox; TF-OTX, orthodenticle homeobox; ZF-MIZ, Myc-interacting zinc finger protein; ZF-HF-X1, NFX1-type zinc finger domains; AP-2, activator protein; CBF, core binding factor; CTF_NFI, nuclear factor CCAAT/I transcription factor; GCM, glial cells missing; HTH, homothorax; PC4, positive cofactor 4; Tub, tubby protein;

**Supplementary Table 5 Statistics of differentially expressed transcripts (DETs) between SW and FW salinity treatment groups**

| DETs set | up-regulation | down-regulation | Total transcripts |
| --- | --- | --- | --- |
| SW vs. FW | 264 | 254 | 518 |

**Supplementary Table 7** **Classification of differentially expressed transcription factors in SW vs. FW.**

| **Name** | **Family** | **Up-regulation** | **Down-regulation** |
| --- | --- | --- | --- |
| zinc finger protein 316  zinc finger protein 316 | zf-C2H2 | √ |  |
| B-cell lymphoma 6 protein | zf-C2H2 | √ |  |
| zinc finger protein 143  zinc finger protein 143 | zf-C2H2 |  | √ |
| zinc finger protein 276 | zf-C2H2 | √ |  |
| zinc finger protein 40 | zf-C2H2 | √ |  |
| zinc finger and BTB domain-containing protein 38  aacontain | zf-C2H2 | √ |  |
| max dimerization protein 4 | bHLH | √ |  |
| circadian locomoter output cycles protein kaput | bHLH | √ |  |
| circadian locomoter output cycles protein kaput-like | bHLH | √ |  |
| transcription factor ETV5 | ETS |  | √ |
| transcription factor ETV6  transcription factor ETV6 | ETS | √ |  |
| transcription factor ERF | ETS | √ |  |
| forkhead box protein l1 | Fork head |  | √ |
| transcription factor sox 13 | HMG | √ |  |
| zinc finger homebox protein 3 | Homeobox | √ |  |
| bromodomain adjacent to zinc finger domain protein 2B | MBD | √ |  |
| transcription factor RelB | RHD | √ |  |

Abbreviations: C2H2-ZF, C2H2-type zinc finger; bHLH, basic helix-loop-helix; RHD, Rel homology domain; HMG, high mobility group; MDB, Methyl-CpG-binding domain.
