# Supplementary material for: Characterization of Full-Length Transcriptome Sequences and Splice Variants of Lateolabrax maculatus by Single-Molecule Long-Read Sequencing and Their Involvement in Salinity Regulation
Source: Front Genet. 2019 Nov 15;10:1126. doi: 10.3389/fgene.2019.01126 (PMC6873903; doi:10.3389/fgene.2019.01126)
Supplement: Supplementary file 12 [file Table_6.docx]

**Supplementary Table 6 Functional classification of DETs in SW vs. FW.**

| Energy metabolism | Gene ID | Isoforms ID | FDR | Log_2_FC | Classify |
| --- | --- | --- | --- | --- | --- |
| V-type proton ATPase 16 kDa proteolipid subunit | evm.TU.scaffold_4.320 | PB.9895.1 | 0.874168 | 0.164818 | 1 |
|  |  | **PB.9895.2** | **0.000453** | **-7.03095** | **2** |
|  |  | PB.9895.3 | 1 | 0.007948 | 2 |
|  |  | PB.9895.4 | 0.730144 | -0.3165 | 2 |
|  |  |  |  |  |  |
| cytoplasmic aconitate hydratase | evm.TU.scaffold_99.27 | PB.15064.1 | 0.033068 | 0.750996 | 1 |
|  |  | **PB.15064.2** | **5.27E-06** | **-5.80882** | **2** |
|  |  | PB.15064.3 |  |  | 2 |
|  |  | PB.15064.4 |  |  | 2 |
|  |  |  |  |  |  |
| GDH/6PGL endoplasmic bifunctional protein | evm.TU.scaffold_76.110 | PB.13279.5 | 0.64779 | 1.461606 | 1 |
|  |  | PB.13279.1 |  |  | 2 |
|  |  | PB.13279.10 |  |  | 2 |
|  |  | PB.13279.11 |  |  | 2 |
|  |  | PB.13279.12 |  |  | 2 |
|  |  | PB.13279.13 |  |  | 2 |
|  |  | PB.13279.14 |  |  | 2 |
|  |  | PB.13279.15 |  |  | 2 |
|  |  | PB.13279.2 |  |  | 2 |
|  |  | PB.13279.3 |  |  | 2 |
|  |  | **PB.13279.4** | **0.026324** | **-5.61658** | **2** |
|  |  | PB.13279.6 |  |  | 2 |
|  |  | PB.13279.7 |  |  | 2 |
|  |  | PB.13279.8 |  |  | 2 |
|  |  | PB.13279.9 | 0.481427 | 1.143552 | 2 |
|  |  |  |  |  |  |
| alpha-1,6-mannosylglycoprotein 6-beta-N-acetylglucosaminyltransferase A | evm.TU.scaffold_55.3 | evm.scaffold  _55.3 | 0.000671 | 5.207337 | 1 |
|  |  |  |  |  |  |
| 6-phosphofructo-2-kinase/fructose-2,6-bisphosphatase 3 | evm.TU.scaffold_118.17 | **PB.1707.1** | **6.17E-29** | **lnf** | **2** |
|  |  | PB.1707.3 |  |  | 2 |
|  |  | **PB.1707.4** | **2.61E-23** | **-5.16585** | **2** |
|  |  | PB.1707.5 | 0.678407 | 1.237325 | 2 |
|  |  | PB.1707.6 | 0.896209 | 0.646193 | 1 |
|  |  |  |  |  |  |
| peroxisome proliferator-activated receptor gamma coactivator-related protein 1-like | evm.TU.scaffold_188.34 | PB.4741.2 | 0.14989 | 1.462544 | 2 |
|  |  | **PB.4741.3** | **0.000844** | **-4.67244** | **2** |
|  |  |  |  |  |  |
| alpha-1,6-mannosylglycoprotein 6-beta-N-acetylglucosaminyltransferase A-like | evm.TU.scaffold_55.3 | **PB.11396.1** | **1.89E-09** | **4.31231** | **2** |
|  |  |  |  |  |  |
| Glycerol-3-phosphate acyltransferase 1, mitochondrial | evm.TU.scaffold_4.132 | PB.9791.2 | 0.931604 | 0.17849 | 1 |
|  |  | **PB.9791.1** | **0.018334** | **-4.19066** | **2** |
|  |  |  |  |  |  |
| enteropeptidase | evm.TU.scaffold_247.19 | PB.6809.1 | 0.134903 | -3.42064 | 2 |
|  |  | **PB.6809.2** | **3.05E-05** | **-2.66928** | **2** |
|  |  |  |  |  |  |
| V-type proton ATPase 21 kDa proteolipid subunit | evm.TU.scaffold_17.193 | PB.4197.1 | 0.904774 | -0.13182 | 1 |
|  |  | PB.4197.2 |  |  | 2 |
|  |  | **PB.4197.3** | **0.000219** | **3.093752** | **2** |
|  |  |  |  |  |  |
| tissue alpha-L-fucosidase | evm.TU.scaffold_173.26 | **evm.model.**  **scaffold_**  **17.193** | **0.00005** | **2.88280** | **1** |
|  |  |  |  |  |  |
| 2,4-dienoyl-CoA reductase, mitochondrial | evm.TU.scaffold_313.25 | PB.8538.2 | 0.025641 | 1.046642 | 1 |
|  |  | **PB.8538.1** | **2.65E-06** | **-2.72487** | **2** |
|  |  | PB.8538.3 | 0.240201 | 0.50909 | 2 |
|  |  | PB.8538.4 | 0.923984 | 0.117931 | 2 |
|  |  |  |  |  |  |
| beta-1,4-galactosyltransferase 5-like | evm.TU.scaffold_211.21_evm.TU.scaffold_211.22 | **PB.5732.2** | **0.015271** | **2.651818** | **1** |
|  |  | PB.5732.1 | 0.886325 | 0.150037 | 2 |
|  |  | PB.5732.10 |  |  | 2 |
|  |  | PB.5732.3 |  |  | 2 |
|  |  | PB.5732.4 |  |  | 2 |
|  |  | PB.5732.5 | 0.450022 | 1.498792 | 2 |
|  |  | PB.5732.6 |  |  | 2 |
|  |  | PB.5732.7 |  |  | 2 |
|  |  | PB.5732.8 |  |  | 2 |
|  |  | PB.5732.9 |  |  | 2 |
|  |  |  |  |  |  |
| glycogen synthase kinase-3 bet | evm.TU.scaffold_32.96 | **PB.8661.2** | **0.03093** | **2.305265** | **1** |
|  |  | PB.8661.1 | 0.695572 | -0.57095 | 2 |
|  |  | PB.8661.3 | 0.257969 | 1.66952 | 2 |
|  |  |  |  |  |  |
| peroxisome proliferator-activated receptor alpha-like | evm.TU.scaffold_28.51 | **PB.7619.1** | **0.028172** | **-2.24357** | **2** |
|  |  |  |  |  |  |
| diacylglycerol kinase eta | evm.TU.scaffold_202.36_evm.TU.scaffold_202.37 | **Evm.model.scaffold_202.36_evm.**  **model.scaffold_202.37** | **0.006083** | **1.991574** | **1** |
|  |  |  |  |  |  |
| group XV phospholipase A2 | evm.TU.scaffold_195.5 | **PB.4951.1** | **9.68E-05** | **1.698925** | **1** |
|  |  | PB.4951.2 | 0.946515 | 0.09407 | 2 |
|  |  | PB.4951.3 | 0.462547 | -0.71901 | 2 |
|  |  | PB.4951.4 | 0.808874 | 0.420559 | 2 |
|  |  |  |  |  |  |
| oxysterol-binding protein-related protein 1-like | evm.TU.scaffold_231.8 | **PB.6317.5** | **0.03886** | **1.659137** | **1** |
|  |  | PB.6317.1 | 0.876746 | -0.61084 | 2 |
|  |  | PB.6317.2 | 0.850715 | 1.360547 | 2 |
|  |  | PB.6317.3 | 0.946483 | 0.126759 | 2 |
|  |  | PB.6317.4 | 0.016341 | 0.795805 | 2 |
|  |  | PB.6317.6 | 0.404154 | -1.52495 | 2 |
|  |  |  |  |  |  |
| mitochondrial carnitine/acylcarnitine carrier protein | evm.TU.scaffold_141.5 | evm.model.scaffold_141.5 | 0.035032 | 1.575605 | 1 |
|  |  |  |  |  |  |
| insulin receptor substrate 1 | evm.TU.scaffold_331.2 | **PB.8867.1** | **0.020868** | **1.538544** | **2** |
|  |  | PB.8867.2 | 0.994086 | 0.157378 | 2 |
|  |  |  |  |  |  |
| Hemoglobin subunit beta-2 | evm.TU.scaffold_23.242 | PB.6294.1 | 0.2896 | -0.77141 |  |
|  |  | **PB.6294.2** | **0.031768** | **-1.58705** |  |
|  |  |  |  |  |  |
| pyruvate kinase PKM-like | evm.TU.scaffold_66.40 | PB.12359.1 | 0.22248 | 0.672566 | 2 |
|  |  | PB.12359.2 | 0.957135 | -0.07782 | 2 |
|  |  | PB.12359.3 | 0.359507 | 1.057283 | 2 |
|  |  | PB.12359.4 | 0.312567 | -1.18359 | 2 |
|  |  | **PB.12359.5** | **0.014652** | **-1.47** | **2** |
|  |  | PB.12359.6 | 0.736324 | 0.410197 | 2 |
|  |  |  |  |  |  |
| polypeptide N-acetylgalactosaminyltransferase 10 | evm.TU.scaffold_97.8_evm.TU.scaffold_97.7 | **evm.model.scaffold_97.8_evm.model.scaffold_97.7** | **0.0107693** | **1.4109822** | **1** |
|  |  |  |  |  |  |
| globoside alpha-1,3-N-acetylgalactosaminyltransferase 1-like | evm.TU.scaffold_205.14 | PB.5445.3 | 0.861705 | -0.1687 | 1 |
|  |  | PB.5445.1 | 0.191108 | -4.37091 | 2 |
|  |  | PB.5445.2 | 0.793369 | -2.08542 | 2 |
|  |  | **PB.5445.4** | **0.038105** | **-1.4342** | **2** |
|  |  | PB.5445.5 | 0.984592 | -0.09335 | 2 |
|  |  |  |  |  |  |
| ATP-binding cassette sub-family A member 1-like | evm.TU.scaffold_69.8 | **PB.12497.5** | **0.000197** | **1.293892** | **1** |
|  |  | PB.12496.1 |  |  | 2 |
|  |  | PB.12497.1 | 0.981574 | -0.05131 | 2 |
|  |  | PB.12497.2 | 0.655898 | -0.29298 | 2 |
|  |  |  |  |  |  |
| fatty acid synthase | evm.TU.scaffold_22.6 | **evm.model**  **Scaffold_22.6** | **0.012214371** | **1.16207028** | **1** |
|  |  |  |  |  |  |
| Retinol dehydrogenase 11 (All-trans/9-cis/11-cis) | evm.TU.scaffold_349.13 | **PB.9067.3** | **4.29E-05** | **-1.08506** | **1** |
|  |  | PB.9067.1 | 0.489608 | -0.89206 | 2 |
|  |  | PB.9067.2 | 0.110464 | -1.16308 | 2 |
|  |  |  |  |  |  |
| monoglyceride lipase | evm.TU.scaffold_175.30 | **PB.4329.2** | **0.001809** | **1.186127** | **1** |
|  |  | PB.4329.1 | 0.576221 | -0.32986 | 2 |
|  |  |  |  |  |  |
| lipoprotein lipase | evm.TU.scaffold_9.117 | **PB.14378.1** | **0.044961** | **-1.16574** | **1** |
|  |  |  |  |  |  |
| UDP-glucuronosyltransferase-like | evm.TU.scaffold_65.29 | PB.12319.3 | 0.209263 | -0.71336 | 1 |
|  |  | **PB.12319.1** | **0.038001** | **-1.1614** | **2** |
|  |  | PB.12319.4 |  |  | 2 |
|  |  | PB.12319.5 | 0.918735 | -0.24079 | 2 |
|  |  |  |  |  |  |
| iron-sulfur cluster assembly enzyme ISCU, mitochondrial | evm.TU.scaffold_63.100 | PB.12236.1 | 0.767381 | -0.28285 | 1 |
|  |  | **PB.12236.2** | **0.019376** | **-1.02463** | **2** |
|  |  |  |  |  |  |
| diacylglycerol kinase alpha-like | evm.TU.scaffold_86.39 | PB.14037.1 | 0.033816 | 1.078 | 2 |
|  |  |  |  |  |  |
| hemoglobin alpha | evm.TU.scaffold_23.243 | PB.6295.2 | 0.055891 | -0.87024 | 1 |
|  |  | PB.6295.1 | 0.892261 | 0.902517 | 2 |
|  |  | **PB.6295.3** | **0.044374** | **-1.00348** | **2** |
|  |  | PB.6295.4 | 0.61318 | -0.89108 | 2 |
|  |  |  |  |  |  |
| medium-chain specific acyl-CoA dehydrogenase, mitochondrial-like | evm.TU.scaffold_59.35 | PB.11743.1 | 0.924453 | 0.109583 | 1 |
|  |  | **PB.11743.2** | **1.13E-06** | **#NAME?** | **2** |
|  |  | PB.11743.3 |  |  | 2 |
|  |  |  |  |  |  |
| hormone-sensitive lipase | evm.TU.scaffold_24.152 | **evm.model.scaffold_24.125** | **0.000501781** | **inf** | **1** |

| Immune response | Gene ID | Isoforms ID | FDR | Log_2_FC | Classify |
| --- | --- | --- | --- | --- | --- |
| glioma tumor suppressor candidate region gene 1 protein | evm.TU.scaffold_16.127 | **PB.3791.1** | **0.009169** | **6.289124** | **2** |
|  |  | PB.3792.1 | 0.689144 | -0.78568 | 2 |
|  |  |  |  |  |  |
| complement C4 | evm.TU.scaffold_98.92 | PB.15020.1 |  |  | 2 |
|  |  | PB.15020.10 |  |  | 2 |
|  |  | PB.15020.11 |  |  | 2 |
|  |  | PB.15020.12 |  |  | 2 |
|  |  | PB.15020.13 |  |  | 2 |
|  |  | PB.15020.14 |  |  | 2 |
|  |  | PB.15020.15 |  |  | 2 |
|  |  | PB.15020.17 |  |  | 2 |
|  |  | PB.15020.18 |  |  | 2 |
|  |  | PB.15020.19 | 0.977714 | -0.08434 | 2 |
|  |  | PB.15020.2 | 1 | 0.065274 | 2 |
|  |  | **PB.15020.20** | **0.000989** | **-4.51151** | **2** |
|  |  | PB.15020.21 | 0.746807 | -0.93763 | 2 |
|  |  | PB.15020.22 | 0.902712 | 0.880013 | 2 |
|  |  | PB.15020.23 | 0.624546 | -0.67121 | 2 |
|  |  | PB.15020.24 | 0.161779 | -1.41438 | 2 |
|  |  | PB.15020.3 |  |  | 2 |
|  |  | PB.15020.4 | 0.986647 | -0.10973 | 2 |
|  |  | PB.15020.5 |  |  | 2 |
|  |  | PB.15020.6 |  |  | 2 |
|  |  | PB.15020.7 | 0.970184 | -0.46962 | 2 |
|  |  | PB.15020.8 | 0.856883 | 0.651135 | 2 |
|  |  | PB.15020.9 | 0.148579 | 1.697843 | 2 |
|  |  |  |  |  |  |
| H-2 class I histocompatibility antigen, Q10 alpha chain | evm.TU.scaffold_190.20 | PB.4879.4 | 0.848975 | 1.00512 | 1 |
|  |  | PB.4879.1 | 0.736681 | 0.256155 | 2 |
|  |  | PB.4879.10 |  |  | 2 |
|  |  | PB.4879.11 |  |  | 2 |
|  |  | PB.4879.12 |  |  | 2 |
|  |  | PB.4879.13 | 0.890442 | 0.161663 | 2 |
|  |  | PB.4879.14 | 0.114631 | -1.27012 | 2 |
|  |  | PB.4879.15 | 0.618725 | 0.697585 | 2 |
|  |  | PB.4879.16 | 0.970572 | 0.296176 | 2 |
|  |  | PB.4879.17 | 1 | 0.028811 | 2 |
|  |  | PB.4879.18 |  |  | 2 |
|  |  | PB.4879.19 | 0.74395 | 0.860847 | 2 |
|  |  | PB.4879.3 |  |  | 2 |
|  |  | PB.4879.5 | 0.348939 | -0.49983 | 2 |
|  |  | PB.4879.7 | 0.6176 | -3.07717 | 2 |
|  |  | **PB.4879.8** | **0.020792** | **3.832912** | **2** |
|  |  | PB.4879.9 | 0.961393 | -0.67326 | 2 |
|  |  |  |  |  |  |
| interleukin-31 receptor subunit alpha | evm.TU.scaffold_1.489 | PB.586.1 | 0.286494 | -3.72787 | 2 |
|  |  | PB.586.2 | 0.78066 | 0.226032 | 2 |
|  |  | PB.586.3 |  |  | 2 |
|  |  | **PB.586.4** | **3.32E-07** | **#NAME?** | **2** |
|  |  |  |  |  |  |
| death-associated protein kinase 1 | evm.TU.scaffold_85.4 | **PB.13960.1** | **0.000668** | **3.301677** | **2** |
|  |  | PB.13960.2 | 0.920693 | -0.69748 | 2 |
|  |  | PB.13960.3 |  |  | 2 |
|  |  |  |  |  |  |
| interferon-induced very large GTPase 1 | evm.TU.scaffold_56.81 | **PB.11500.2** | **1.16E-17** | **-2.07765** | **1** |
|  |  | **PB.11500.1** | **1.25E-24** | **-3.27868** | **2** |
|  |  |  |  |  |  |
| tumor necrosis factor receptor superfamily member 6B | evm.TU.scaffold_50.72 | PB.11144.2 | 0.583911 | 0.355679 | 1 |
|  |  | PB.11144.1 | 0.645455 | 1.650684 | 2 |
|  |  | PB.11144.10 |  |  | 2 |
|  |  | PB.11144.11 |  |  | 2 |
|  |  | PB.11144.12 |  |  | 2 |
|  |  | PB.11144.13 |  |  | 2 |
|  |  | PB.11144.14 |  |  | 2 |
|  |  | PB.11144.15 | 0.952087 | 0.45962 | 2 |
|  |  | PB.11144.16 |  |  | 2 |
|  |  | PB.11144.3 |  |  | 2 |
|  |  | PB.11144.4 | 0.882373 | -0.88532 | 2 |
|  |  | PB.11144.5 |  |  | 2 |
|  |  | PB.11144.6 |  |  | 2 |
|  |  | PB.11144.7 |  |  | 2 |
|  |  | PB.11144.8 |  |  | 2 |
|  |  | **PB.11144.9** | **2.33E-05** | **3.047581** | **2** |
|  |  |  |  |  |  |
| IgGFc-binding protein-like | evm.TU.scaffold_4.204 | **PB.9838.1** | **0.002537** | **-2.91942** | **2** |
|  |  |  |  |  |  |
| thromboxane-A synthase | evm.TU.scaffold_35.57 | **PB.9103.1** | **0.006127** | **2.758802** | **1** |
|  |  | PB.9103.2 |  |  | 1 |
|  |  |  |  |  |  |
| interleukin-7 receptor subunit alpha | evm.TU.scaffold_104.118 | PB.998.1 | 0.545859 | 0.476187 | 2 |
|  |  | **PB.998.2** | **0.012162** | **2.425454** | **2** |
|  |  | PB.998.3 |  |  | 2 |
|  |  |  |  |  |  |
| pro-interleukin-16 | evm.TU.scaffold_184.33 | **PB.4642.1** | **0.031384** | **-2.3958** | **2** |
|  |  |  |  |  |  |
| hemicentin-1 | evm.TU.scaffold_24.135 | PB.6586.1 | 0.01038 | 0.842269 | 2 |
|  |  | **PB.6586.2** | **2.59E-10** | **-2.37316** | **2** |
|  |  |  |  |  |  |
| tax1-binding protein 1 homolog B | evm.TU.scaffold_98.71 | **PB.15008.1** | **0.011454** | **2.332973** | **2** |
|  |  | PB.15008.2 | 0.913589 | 0.127102 | 2 |
|  |  | PB.15008.3 |  |  | 2 |
|  |  | PB.15008.4 | 0.856883 | 0.68496 | 2 |
|  |  | PB.15008.5 |  |  | 2 |
|  |  | PB.15008.6 | 0.941421 | -1.09188 | 2 |
|  |  | PB.15008.7 |  |  | 2 |
|  |  | PB.15008.8 |  |  | 2 |
|  |  |  |  |  |  |
| mesothelin | evm.TU.scaffold_94.26 | **PB.14793.1** | **5E-05** | **-2.28383** | **2** |
|  |  | PB.14793.2 | 0.164885 | -1.18752 | 2 |
|  |  |  |  |  |  |
| sterile alpha motif domain-containing protein 9 | evm.TU.scaffold_168.28 | PB.4019.1 |  |  | 2 |
|  |  | **PB.4019.2** | **0.039728** | **2.033916** | **2** |
|  |  | PB.4019.3 | 0.700656 | 1.231975 | 2 |
|  |  | PB.4019.4 | 0.981074 | -0.17805 | 2 |
|  |  |  |  |  |  |
| alpha-elapitoxin-As2a | evm.TU.scaffold_47.156 | **PB.10721.1** | **0.01786** | **-1.90024** | **1** |
|  |  |  |  |  |  |
| butyrophilin-like protein 1 | PB.9466 | PB.9466.1 |  |  | 2 |
|  |  | **PB.9466.2** | **0.000123** | **-1.61297** | **2** |
|  |  | PB.9466.3 |  |  | 2 |
|  |  |  |  |  |  |
| collectin-12 | evm.TU.scaffold_17.231 | PB.4216.1 | 1 | -0.00937 | 2 |
|  |  | PB.4216.2 | 0.892976 | 0.431273 | 2 |
|  |  | **PB.4216.3** | **0.01101** | **-1.85531** | **2** |
|  |  | PB.4216.5 |  |  | 2 |
|  |  |  |  |  |  |
| protamine-like protein | evm.TU.scaffold_3.166 | **PB.8079.1** | **1.96E-06** | **-1.58163** | **1** |
|  |  |  |  |  |  |
| immunoglobulin light chain | evm.TU.scaffold_424.2 | PB.10307.1 |  |  | 2 |
|  |  | PB.10307.10 | 0.912708 | -1.13789 | 2 |
|  |  | PB.10307.11 |  |  | 2 |
|  |  | PB.10307.12 |  |  | 2 |
|  |  | PB.10307.13 | 0.913589 | 0.946511 | 2 |
|  |  | PB.10307.14 |  |  | 2 |
|  |  | **PB.10307.15** | **0.000283** | **-1.46194** | **2** |
|  |  | PB.10307.16 | 0.891761 | -0.14849 | 2 |
|  |  | PB.10307.18 | 0.618862 | 1.999777 | 2 |
|  |  | PB.10307.2 | 0.523414 | 3.045356 | 2 |
|  |  | PB.10307.3 | 1 | -0.00982 | 2 |
|  |  | PB.10307.4 |  |  | 2 |
|  |  | PB.10307.5 |  |  | 2 |
|  |  | PB.10307.6 |  |  | 2 |
|  |  | PB.10307.7 |  |  | 2 |
|  |  | PB.10307.8 |  |  | 2 |
|  |  | PB.10307.9 |  |  | 2 |
|  |  |  |  |  |  |
| classical MHC class I molecule, alpha-chain | PB.3171 | PB.3171.1 | 0.947327 | -0.20302 | 2 |
|  |  | PB.3171.2 | 0.055862 | -2.54951 | 2 |
|  |  | PB.3171.3 |  |  | 2 |
|  |  | PB.3171.4 | 0.960506 | 0.126987 | 2 |
|  |  | PB.3171.5 | 0.894264 | -0.61477 | 2 |
|  |  | PB.3171.6 | 0.001614 | 0.969603 | 2 |
|  |  | PB.3171.7 | 0.965985 | -0.12078 | 2 |
|  |  | PB.3171.8 | 0.906112 | -0.29242 | 2 |
|  |  | **PB.3171.9** | **0.027858** | **1.387451** | **2** |
|  |  |  |  |  |  |
| leucine-rich-repeat-containing protein C3 | PB.8905 | PB.8905.1 | 0.941639 | -0.09465 | 2 |
|  |  | **PB.8905.2** | **0.003756** | **-1.30787** | **2** |
|  |  |  |  |  |  |
| sterile alpha motif domain-containing protein 9 | evm.TU.scaffold_114.18 | **PB.1555.1** | **2.07E-06** | **1.218328** | **1** |
|  |  |  |  |  |  |
| nuclear factor of activated T-cells, cytoplasmic 2 | evm.TU.scaffold_57.15 | PB.11562.1 | 0.8507 | -0.19147 | 1 |
|  |  | **PB.11562.2** | **0.009357** | **1.133838** | **2** |
|  |  |  |  |  |  |
| leukocyte receptor cluster member 9 | evm.TU.scaffold_240.5 | PB.6671.1 | 0.850968 | -0.19266 | 1 |
|  |  | **PB.6671.3** | **0.005096** | **1.089979** | **2** |
|  |  | PB.6671.4 | 0.998973 | -0.04105 | 2 |
|  |  |  |  |  |  |
| lipolysis-stimulated lipoprotein receptor | evm.TU.scaffold_387.3 | PB.9616.2 | 0.887696 | 0.148712 | 1 |
|  |  | **PB.9616.1** | **1.55E-07** | **inf** | **2** |
|  |  | PB.9616.3 |  |  | 2 |
|  |  |  |  |  |  |
| caspase-1-A | evm.TU.scaffold_56.153 | PB.11547.1 | 0.528734 | -0.35189 | 2 |
|  |  | **PB.11547.2** | **0.00029** | **inf** | **2** |

| Ion and amino transport and metabolism | Gene ID | Isoforms ID | FDR | Log_2_FC | Classify |
| --- | --- | --- | --- | --- | --- |
| cysteine dioxygenase type 1-like | evm.TU.scaffold_3.79 | **PB.8015.1** | **1.42E-15** | **-6.26435354** | **1** |
|  |  | **PB.8015.2** | **2.13E-11** | **-5.18024918** | **2** |
|  |  | **PB.8015.3** | **6.58E-23** | **-5.03618275** | **2** |
|  |  |  |  |  |  |
| putative adenosylhomocysteinase 3 | evm.TU.scaffold_120.43 | PB.1942.1 | 0.943696 | 0.103465756 | 2 |
|  |  | **PB.1942.2** | **0.039827** | **5.187200535** | **2** |
|  |  | PB.1942.3 |  |  | 2 |
|  |  |  |  |  |  |
| zinc transporter 4 | evm.TU.scaffold_254.19 | PB.7007.2 | 0.893757 | -0.14124554 | 1 |
|  |  | **PB.7007.3** | **0.005667** | **3.885455518** | **2** |
|  |  |  |  |  |  |
| glutamine synthetase 1 | evm.TU.scaffold_31.56 | PB.8444.2 | 0.123611 | 0.561723058 | 1 |
|  |  | **PB.8444.1** | **0.002384** | **-4.97129057** | **2** |
|  |  |  |  |  |  |
| solute carrier family 25 member 39 | evm.TU.scaffold_340.13 | PB.9018.1 | 0.941951 | -0.12486667 | 2 |
|  |  | **PB.9018.2** | **0.001055** | **4.119187713** | **2** |
|  |  |  |  |  |  |
| anoctamin-9 | evm.TU.scaffold_233.50 | PB.6375.6 | 0.946515 | -0.32976727 | 1 |
|  |  | PB.6375.1 | 0.997091 | -0.15588942 | 2 |
|  |  | **PB.6375.2** | **0.039827** | **3.5009615** | **2** |
|  |  | PB.6375.3 | 0.983846 | 0.042653303 | 2 |
|  |  | PB.6375.4 | 0.106328 | 2.498232883 | 2 |
|  |  | PB.6375.5 | 0.71193 | 1.154274621 | 2 |
|  |  |  |  |  |  |
| sodium/potassium-transporting ATPase subunit alpha-2 | evm.TU.scaffold_365.3 | PB.9325.1 | 0.914522 | 0.122199277 | 1 |
|  |  | **PB.9325.2** | **0.025652** | **-3.18805644** | **2** |
|  |  | PB.9325.3 | 0.488076 | 1.501912216 | 2 |
|  |  |  |  |  |  |
| Band 3 anion exchange protein | evm.TU.scaffold_4.202 | **PB.9837.1** | **0.001055** | **-2.84613256** | **2** |
|  |  | PB.9837.2 | 0.913684 | 0.121243109 | 2 |
|  |  |  |  |  |  |
| methionine adenosyltransferase 2 subunit beta | evm.TU.scaffold_411.1 | **PB.10221.1** | **0.008135** | **2.648328596** | **1** |
|  |  | PB.10221.2 | 0.314446 | -0.53618044 | 2 |
|  |  | PB.10221.3 | 0.225686 | 2.926855602 | 2 |
|  |  | PB.10221.4 | 0.015232 | -0.98821995 | 2 |
|  |  | PB.10221.5 |  |  | 2 |
|  |  | PB.10221.6 |  |  | 2 |
|  |  |  |  |  |  |
| N-acylneuraminate cytidylyltransferase | evm.TU.scaffold_290.9 | PB.7881.3 | 0.882373 | 0.16741625 | 1 |
|  |  | **PB.7881.1** | **6.78E-18** | **-2.41866373** | **2** |
|  |  | PB.7881.2 | 0.554414 | 0.617711536 | 2 |
|  |  | PB.7881.4 | 0.193762 | 0.611428455 | 2 |
|  |  |  |  |  |  |
| ornithine decarboxylase | evm.TU.scaffold_68.82 | PB.12487.1 | 0.626686 | -0.39131358 | 1 |
|  |  | **PB.12487.3** | **0.000393** | **-2.16436984** | **2** |
|  |  | PB.12487.4 | 0.172329 | -0.66285723 | 2 |
|  |  |  |  |  |  |
| potassium channel subfamily K member 6 | evm.TU.scaffold_16.69 | PB.3760.2 | 0.887447 | 0.217068275 | 1 |
|  |  | PB.3760.1 | 0.387477 | 0.786310022 | 2 |
|  |  | **PB.3760.3** | **0.037279** | **2.143549885** | **2** |
|  |  |  |  |  |  |
| carbonic anhydrase | evm.TU.scaffold_4.268 | **PB.9876.1** | **9.08E-12** | **1.875221957** | **2** |
|  |  | PB.9876.2 | 0.886161 | 0.183430059 | 2 |
|  |  | PB.9876.3 | 1 | 0.01486121 | 2 |
|  |  | PB.9876.4 | 0.804409 | 0.296319166 | 2 |
|  |  |  |  |  |  |
| ATP-binding cassette sub-family A member 12 | evm.TU.scaffold_45.123 | **PB.10550.1** | **0.000348** | **-2.08587672** | **2** |
|  |  | PB.10550.2 |  |  | 2 |
|  |  |  |  |  |  |
| protein S100-A13-like | evm.TU.scaffold_47.91 | **PB.10680.1** | **1.44E-11** | **-1.84698855** | **2** |
|  |  |  |  |  |  |
| sodium/potassium-transporting ATPase subunit beta-233 | evm.TU.scaffold_125.24 | **PB.2116.24** | **5.48E-05** | **-1.31694649** | **1** |
|  |  | **PB.2116.25** | **0.044374** | **-1.59047126** | **2** |
|  |  | **PB.2116.26** | **1.22E-05** | **-1.79317407** | **2** |
|  |  |  |  |  |  |
| erythrocyte band 7 integral membrane protein | evm.TU.scaffold_31.77 | PB.8451.1 | 0.977714 | -0.10831575 | 2 |
|  |  | PB.8451.2 | 0.083313 | -0.77112438 | 2 |
|  |  | **PB.8451.3** | **0.007222** | **1.735113985** | **2** |
|  |  | PB.8451.4 |  |  | 2 |
|  |  |  |  |  |  |
| sodium/hydrogen exchanger 2 | evm.TU.scaffold_32.21 | **PB.8611.1** | **0.001194** | **1.518978261** | **1** |
|  |  |  |  |  |  |
| enolase-phosphatase E1 | evm.TU.scaffold_59.26 | PB.11737.2 | 0.000343 | 0.982447667 | 1 |
|  |  | **PB.11737.1** | **1.49E-06** | **1.712183055** | **2** |
|  |  |  |  |  |  |
| NKCC1 | evm.TU.scaffold_71.42 | **PB.12830.1** | **1.08E-06** | **1.466364288** | **2** |
|  |  | PB.12830.2 |  |  | 2 |
|  |  |  |  |  |  |
| sarcoplasmic/endoplasmic reticulum calcium ATPase 3 | evm.TU.scaffold_58.90_evm.TU.scaffold_58.91 | PB.11685.1 | 0.118128 | 1.453998358 | 2 |
|  |  | **PB.11685.2** | **1.39E-05** | **1.437575929** | **2** |
|  |  | PB.11685.3 |  |  | 2 |
|  |  | PB.11685.4 |  |  | 2 |
|  |  |  |  |  |  |
| sodium/bile acid cotransporter 7 | evm.TU.scaffold_341.11_evm.TU.scaffold_341.12 | **PB.9028.2** | **0.000995** | **1.429816799** | **1** |
|  |  | PB.9028.1 | 0.394535 | -0.72752758 | 2 |
|  |  | PB.9028.3 |  |  | 2 |
|  |  | PB.9028.4 |  |  | 2 |
|  |  | PB.9028.5 |  |  | 2 |
|  |  |  |  |  |  |
| cystic fibrosis transmembrane conductance regulator | evm.TU.scaffold_36.139_evm.TU.scaffold_36.138 | **PB.9295.1** | **3.47E-05** | **1.254410559** | **1** |
|  |  | PB.9294.1 |  |  | 2 |
|  |  |  |  |  |  |
| RhCG1 | evm.TU.scaffold_6.63 | **PB.11850.1** | **3.71E-07** | **-1.3024696** | **2** |
|  |  | PB.11850.2 | 0.979299 | -0.14498514 | 2 |
|  |  |  |  |  |  |
| arginine/serine-rich protein 1 | evm.TU.scaffold_223.28 | PB.6036.2 | 0.038807 | -0.91237715 | 1 |
|  |  | PB.6036.1 | 0.831604 | 0.214493885 | 2 |
|  |  | PB.6036.3 | 0.876746 | -0.80907447 | 2 |
|  |  | **PB.6036.4** | **8.14E-07** | **-1.25272223** | **2** |
|  |  | PB.6036.5 | 1 | 0.003981476 | 2 |
|  |  | PB.6036.6 | 0.788685 | 0.325317768 | 2 |
|  |  |  |  |  |  |
| eosinophil peroxidase-like | evm.TU.scaffold_3.275 | **PB.8142.1** | **0.034203** | **1.247419334** | **2** |
|  |  |  |  |  |  |
| inward rectifier potassium channel 16-like | evm.TU.scaffold_72.34 | **PB.12892.1** | **0.026295** | **-1.13458859** | **2** |
|  |  |  |  |  |  |
| leucyl-cystinyl aminopeptidase | evm.TU.scaffold_3.372 | PB.8198.1 | 0.12019 | 0.566445434 | 1 |
|  |  | PB.8197.1 | 0.766713 | 0.956113315 | 2 |
|  |  | PB.8198.2 |  |  | 2 |
|  |  | **PB.8198.3** | **0.002468** | **-1.01154838** | **2** |
|  |  |  |  |  |  |
| neutral amino acid transporter A | evm.TU.scaffold_107.2 | PB.1075.1 |  |  | 2 |
|  |  | **PB.1075.2** | **0.000257** | **#NAME?** | **2** |
|  |  | PB.1075.3 |  |  | 2 |

| Protein biosynthesis | Gene ID | Isoforms ID | FDR | Log_2_FC | Classify |
| --- | --- | --- | --- | --- | --- |
|  |  |  |  |  |  |
| zymogen granule membrane protein 16-like | evm.TU.scaffold_50.7 | **PB.11100.1** | **1.72E-25** | **6.142983** | **2** |
|  |  |  |  |  |  |
| exportin-2 | evm.TU.scaffold_144.32 | PB.3134.1 | 0.987186 | 0.049913 | 1 |
|  |  | PB.3134.2 | 0.823476 | 1.404937 | 2 |
|  |  | PB.3134.3 | 0.369011 | -2.61101 | 2 |
|  |  | PB.3134.4 |  |  | 2 |
|  |  | **PB.3134.5** | **1.81E-06** | **6.122775** | **2** |
|  |  |  |  |  |  |
| coatomer subunit delta | evm.TU.scaffold_62.6 | PB.12120.1 | 0.056123 | 0.684283 | 1 |
|  |  | PB.12120.2 | 0.233116 | 1.061996 | 2 |
|  |  | PB.12120.3 | 0.905991 | -0.93327 | 2 |
|  |  | **PB.12120.4** | **4.85E-14** | **-5.94208** | **2** |
|  |  |  |  |  |  |
| EH domain-containing protein 1 | evm.TU.scaffold_174.6 | PB.4293.2 | 0.898771 | 0.138343 | 1 |
|  |  | PB.4293.1 | 0.890442 | -1.21506 | 2 |
|  |  | **PB.4293.3** | **3.63E-19** | **-5.10991** | **2** |
|  |  |  |  |  |  |
| AP-4 complex subunit beta-1 | evm.TU.scaffold_115.63 | PB.1602.1 | 0.852474 | 0.363127 | 2 |
|  |  | PB.1602.2 | 0.924259 | 0.162672 | 2 |
|  |  | **PB.1602.3** | **8.44E-07** | **-4.89949** | **2** |
|  |  |  |  |  |  |
| conserved oligomeric Golgi complex subunit 7 | evm.TU.scaffold_4.304 | PB.9892.1 | 0.857671 | 0.189305 | 2 |
|  |  | PB.9892.2 |  |  | 2 |
|  |  | PB.9892.3 |  |  | 2 |
|  |  | PB.9892.4 |  |  | 2 |
|  |  | **PB.9892.5** | **0.010499** | **-4.59976** | **2** |
|  |  |  |  |  |  |
| synaptophysin | evm.TU.scaffold_11.205 | **PB.1334.1** | **0.009623** | **-4.03709** | **1** |
|  |  |  |  |  |  |
| secretory carrier-associated membrane protein 2 | evm.TU.scaffold_89.23 | **PB.14261.1** | **2.98E-08** | **3.950843** | **2** |
|  |  | PB.14261.2 | 0.312743 | -0.62351 | 2 |
|  |  |  |  |  |  |
| Golgi apparatus membrane protein TVP23 homolog B | evm.TU.scaffold_72.38 | PB.12894.3 | 0.941951 | -0.10629 | 1 |
|  |  | **PB.12894.1** | **0.035321** | **-3.92552** | **2** |
|  |  | PB.12894.4 | 0.909726 | -0.47466 | 2 |
|  |  |  |  |  |  |
| secretogranin-3 | evm.TU.scaffold_294.15 | **PB.7931.2** | **0.014944** | **3.656472** | **1** |
|  |  | PB.7931.1 | 0.076879 | -2.45729 | 2 |
|  |  | PB.7931.3 |  |  | 2 |
|  |  |  |  |  |  |
| Ras-related protein Rab-5A | evm.TU.scaffold_127.8 | PB.2167.1 | 0.799736 | -0.22058 | 1 |
|  |  | PB.2167.2 | 0.834017 | 0.468093 | 2 |
|  |  | **PB.2167.3** | **0.000228** | **3.462938** | **2** |
|  |  |  |  |  |  |
| protein transport protein Sec61 subunit alpha-like 1 | evm.TU.scaffold_44.72 | PB.10428.1 | 0.627807 | 1.691012 | 1 |
|  |  | **PB.10428.2** | **0.000181** | **-3.22291** | **2** |
|  |  | PB.10428.3 | 0.891708 | 0.144956 | 2 |
|  |  | PB.10428.4 | 0.84794 | 0.815733 | 2 |
|  |  | PB.10428.5 | 0.279367 | 1.820161 | 2 |
|  |  | PB.10428.6 | 0.910865 | -0.24769 | 2 |
|  |  | PB.10428.7 | 0.811013 | -0.70222 | 2 |
|  |  |  |  |  |  |
| Methionyl-tRNA synthetase, cytoplasmic | evm.TU.scaffold_78.35 | PB.13376.1 | 0.681187 | -0.31168 | 1 |
|  |  | **PB.13376.2** | **0.014387** | **-3.00031** | **2** |
|  |  |  |  |  |  |
| kinectin | evm.TU.scaffold_8.178 | PB.13583.1 | 0.970685 | -0.10928 | 2 |
|  |  | PB.13583.2 | 0.447569 | 0.404469 | 2 |
|  |  | PB.13583.3 |  |  | 2 |
|  |  | PB.13583.4 | 0.409158 | 0.614898 | 2 |
|  |  | **PB.13583.5** | **9.52E-06** | **-2.91178** | **2** |
|  |  | PB.13583.6 |  |  | 2 |
|  |  |  |  |  |  |
| secretory carrier-associated membrane protein 2 | evm.TU.scaffold_36.41 | PB.9233.1 | 0.816185 | 0.200789 | 1 |
|  |  | PB.9233.2 |  |  | 2 |
|  |  | PB.9233.3 |  |  | 2 |
|  |  | **PB.9233.4** | **0.04821** | **-2.71789** | **2** |
|  |  |  |  |  |  |
| vacuolar protein sorting-associated protein 29 | evm.TU.scaffold_38.76 | PB.9541.3 | 0.78716 | -0.24116 | 1 |
|  |  | PB.9541.1 | 0.947547 | -0.09123 | 2 |
|  |  | **PB.9541.2** | **0.006958** | **-2.68354** | **2** |
|  |  | PB.9541.4 | 0.591855 | 0.625103 | 2 |
|  |  | PB.9541.5 | 0.587937 | 0.954818 | 2 |
|  |  |  |  |  |  |
| leucine--tRNA ligase, cytoplasmic | evm.TU.scaffold_13.42 | PB.2319.2 | 0.90037 | 0.149226 | 1 |
|  |  | **PB.2319.1** | **0.003467** | **-2.45834** | **2** |
|  |  |  |  |  |  |
| RING finger protein 223 | evm.TU.scaffold_74.69 | **PB.13125.1** | **1.46E-10** | **-2.35546** | **1** |
|  |  |  |  |  |  |
| RAD50-interacting protein 1 | evm.TU.scaffold_151.18 | PB.3465.2 | 0.906112 | -0.55318 | 2 |
|  |  | PB.3465.21 | 0.867365 | -0.23052 | 2 |
|  |  | PB.3465.22 |  |  | 2 |
|  |  | **PB.3465.23** | **0.008913** | **2.277283** | **2** |
|  |  |  |  |  |  |
| furin-1 | evm.TU.scaffold_30.95 | PB.8286.1 | 0.976289 | 0.049281 | 2 |
|  |  | PB.8286.10 |  |  | 2 |
|  |  | PB.8286.11 |  |  | 2 |
|  |  | PB.8286.12 | 0.649904 | 1.354175 | 2 |
|  |  | PB.8286.2 |  |  | 2 |
|  |  | PB.8286.3 |  |  | 2 |
|  |  | **PB.8286.4** | **0.038001** | **2.28474** | **2** |
|  |  | PB.8286.5 |  |  | 2 |
|  |  | PB.8286.6 |  |  | 2 |
|  |  | PB.8286.7 |  |  | 2 |
|  |  | PB.8286.8 |  |  | 2 |
|  |  | PB.8286.9 |  |  | 2 |
|  |  |  |  |  |  |
| 40S ribosomal protein S6 | evm.TU.scaffold_15.150 | PB.3399.1 | 0.685833 | -0.40562 | 1 |
|  |  | PB.3399.2 | 0.975647 | -0.15936 | 2 |
|  |  | **PB.3399.3** | **0.026324** | **-1.94044** | **2** |
|  |  | PB.3399.4 | 0.800997 | -0.85919 | 2 |
|  |  |  |  |  |  |
| protein disulfide-isomerase A3 | evm.TU.scaffold_36.50 | PB.9245.1 | 0.933117 | 0.103789 | 1 |
|  |  | **PB.9245.2** | **0.000477** | **-1.87125** | **2** |
|  |  |  |  |  |  |
| AP-3 complex subunit delta-1 | evm.TU.scaffold_9.123 | PB.14385.4 | 0.704242 | 0.468794 | 1 |
|  |  | PB.14384.1 | 0.966921 | 0.189886 | 2 |
|  |  | PB.14385.1 | 0.853436 | 0.444092 | 2 |
|  |  | PB.14385.2 | 0.473697 | 2.349141 | 2 |
|  |  | **PB.14385.3** | **0.010172** | **1.620127** | **2** |
|  |  | PB.14385.5 | 0.965503 | 0.066582 | 2 |
|  |  |  |  |  |  |
| protein bicaudal D homolog 2 | evm.TU.scaffold_11.53 | PB.1234.2 | 0.102557 | 0.597482 | 1 |
|  |  | **PB.1234.1** | **0.002019** | **-1.35819** | **2** |
|  |  |  |  |  |  |
| protein wntless homolog | evm.TU.scaffold_73.67 | **PB.13001.1** | **0.034662** | **-1.16244** | **2** |
|  |  |  |  |  |  |
| ras-related protein Rab-27B | evm.TU.scaffold_221.3 | **PB.5989.1** | **0.024659** | **1.041558** | **2** |
|  |  |  |  |  |  |
| ARF GTPase-activating protein GIT1 | evm.TU.scaffold_283.10_evm.TU.scaffold_283.9 | **PB.7719.1** | **0.018173** | **1.031481** | **1** |
|  |  |  |  |  |  |
| protein unc-13 homolog B | evm.TU.scaffold_31.106 | **PB.8472.1** | **0.04522** | **1.031418** | **1** |
|  |  | PB.8472.2 | 0.880454 | -0.41921 | 2 |
|  |  |  |  |  |  |
| epsin-2 | evm.TU.scaffold_156.4 | **PB.3595.1** | **0.01989** | **1.001186** | **1** |
|  |  |  |  |  |  |
| 5&apos;-3&apos; exoribonuclease 2 | evm.TU.scaffold_222.19 | PB.6012.1 | 0.490589 | 0.66334 | 1 |
|  |  | **PB.6012.2** | **0.000326** | **inf** | **2** |
|  |  | PB.6012.3 |  |  | 2 |
|  |  | PB.6012.4 | 0.991046 | 0.037788 | 2 |
|  |  |  |  |  |  |
| ATP-dependent Clp protease ATP-binding subunit clpX-like, mitochondrial | evm.TU.scaffold_233.35 | PB.6369.4 | 0.999559 | -0.01548 | 1 |
|  |  | **PB.6369.1** | **0.000546** | **inf** | **2** |
|  |  | PB.6369.10 |  |  | 2 |
|  |  | PB.6369.11 |  |  | 2 |
|  |  | PB.6369.2 |  |  | 2 |
|  |  | PB.6369.3 |  |  | 2 |
|  |  | PB.6369.5 | 0.892261 | -0.29739 | 2 |
|  |  | PB.6369.6 |  |  | 2 |
|  |  | PB.6369.7 | 0.805421 | 0.251705 | 2 |
|  |  | PB.6369.8 | 0.769502 | -0.81329 | 2 |
|  |  | PB.6369.9 |  |  | 2 |
|  |  |  |  |  |  |
| formin-binding protein 4 | evm.TU.scaffold_35.18 | PB.9080.1 | 0.124326 | -0.95137 | 1 |
|  |  | PB.9080.3 | 0.452905 | 0.394819 | 2 |
|  |  | **PB.9080.4** | **6.32E-40** | **inf** | **2** |
|  |  | PB.9080.5 | 0.885405 | 0.418726 | 2 |
|  |  |  |  |  |  |
| vacuolar protein sorting-associated protein 37A | evm.TU.scaffold_69.23 | PB.12507.1 | 0.602568 | -0.41485 | 1 |
|  |  | **PB.12507.2** | **8.53E-05** | **inf** | **2** |
|  |  | PB.12507.3 | 0.257103 | -1.19319 | 2 |
|  |  |  |  |  |  |
| dnaJ homolog subfamily B member 6-like | evm.TU.scaffold_70.57 | **PB.12783.1** | **0.000425** | **inf** | **2** |
|  |  | PB.12783.2 | 0.861844 | -0.36901 | 2 |
|  |  | PB.12783.3 | 0.469246 | 0.895236 | 2 |

| Protein degradation | Gene ID | Isoforms ID | FDR | Log_2_FC | Classify |
| --- | --- | --- | --- | --- | --- |
| E3 ubiquitin-protein ligase BRE1B | evm.TU.scaffold_4.363 | PB.9928.1 | 0.183095 | 0.700391 | 2 |
|  |  | **PB.9928.2** | **0.001003** | **-4.35622** | **2** |
|  |  |  |  |  |  |
| ubiquitin-like-conjugating enzyme ATG3 | evm.TU.scaffold_125.21 | PB.2117.4 |  |  |  |
|  |  | PB.2117.5 | 0.574845 | 0.328573 | 2 |
|  |  | PB.2117.6 | 0.899096 | 0.368412 | 2 |
|  |  | **PB.2117.7** | **0.002468** | **-2.80038** | **2** |
|  |  | PB.2117.8 | 0.197405 | 0.995258 | 2 |
|  |  | PB.2117.9 |  |  | 2 |
|  |  |  |  |  |  |
| ubiquitin-like modifier-activating enzyme ATG7 | evm.TU.scaffold_111.71 | PB.1487.1 | 0.933835 | 0.11049 | 1 |
|  |  | PB.1487.3 | 0.933835 | 0.11049 | 1 |
|  |  | **PB.1487.2** | **0.031404** | **-2.32177** | **2** |
|  |  |  |  |  |  |
| 26S protease regulatory subunit 10B | evm.TU.scaffold_133.35 | PB.2566.1 | 1 | 0.008811 | 1 |
|  |  | PB.2566.2 | 0.551478 | -1.06086 | 2 |
|  |  | **PB.2566.3** | **0.001158** | **-2.19329** | **2** |
|  |  |  |  |  |  |
| proteasomal ubiquitin receptor ADRM1 | evm.TU.scaffold_141.26 | PB.3039.4 | 0.761897 | 0.892048 | 1 |
|  |  | PB.3039.1 | 0.950776 | -0.08324 | 2 |
|  |  | PB.3039.2 | 0.948983 | 0.308228 | 2 |
|  |  | **PB.3039.3** | **0.008178** | **-1.88669** | **2** |
|  |  |  |  |  |  |
| WD repeat and SOCS box-containing protein 1 | evm.TU.scaffold_62.77 | **PB.12162.3** | **0.005181** | **1.798987** | **1** |
|  |  | PB.12162.1 | 0.089286 | 0.948123 | 2 |
|  |  | PB.12162.2 | 0.85492 | -0.17642 | 2 |
|  |  | PB.12162.4 | 0.298268 | 1.900349 | 2 |
|  |  | PB.12162.5 |  |  | 2 |
|  |  |  |  |  |  |
| proteasome subunit beta type-3 | evm.TU.scaffold_29.54 | PB.7810.1 | 0.714133 | -0.25846 | 1 |
|  |  | PB.7810.2 | 0.930432 | 0.425735 | 2 |
|  |  | **PB.7810.3** | **0.006976** | **-1.74322** | **2** |
|  |  |  |  |  |  |
| E3 ubiquitin-protein ligase HERC2 | evm.TU.scaffold_21.128 | **PB.5647.1** | **0.035054** | **1.195857** | **1** |
|  |  | PB.5646.1 | 0.984143 | 0.052514 | 2 |
|  |  |  |  |  |  |
| EF-hand calcium-binding domain-containing protein 4B | evm.TU.scaffold_35.73 | PB.9109.2 | 0.018173 | 1.132593 | 1 |
|  |  | PB.9109.4 | 0.018173 | 1.132593 | 1 |
|  |  | PB.9109.5 |  |  | 2 |
|  |  |  |  |  |  |
| ubiquitin conjugation factor E4 B | evm.TU.scaffold_5.126 | PB.10955.1 | 0.904774 | -0.14579 | 2 |
|  |  | PB.10955.2 |  |  | 2 |
|  |  | **PB.10955.3** | **0.004504** | **1.087656** | **2** |
|  |  |  |  |  |  |
| ubiquitin carboxyl-terminal hydrolase CYLD | evm.TU.scaffold_44.117 | **PB.10449.1** | **0.004448** | **-1.07334** | **2** |
|  |  |  |  |  |  |
| probable ubiquitin carboxyl-terminal hydrolase FAF-X | evm.TU.scaffold_39.42 | **PB.9646.3** | **0.009847** | **1.044581** | **1** |
|  |  | PB.9646.1 | 0.901477 | -0.48732 | 2 |
|  |  | PB.9646.2 | 0.890396 | 0.49585 | 2 |
|  |  | PB.9646.4 | 0.593585 | -0.36846 | 2 |
|  |  |  |  |  |  |
| E3 ubiquitin-protein ligase RNF213 | evm.TU.scaffold_40.151 | **PB.10041.2** | **0.006232** | **1.023997** | **1** |
|  |  | PB.10041.1 |  |  | 2 |
|  |  | PB.10042.1 | 0.979496 | 0.05919 | 2 |
|  |  | PB.10042.2 | 0.367433 | 1.35785 | 2 |
|  |  |  |  |  |  |
| F-box/LRR-repeat protein 5 | evm.TU.scaffold_21.176 | PB.5674.4 |  |  | 1 |
|  |  | PB.5674.1 | 0.841325 | -0.21291 | 2 |
|  |  | **PB.5674.2** | **0.000404** | **inf** | **2** |
|  |  | PB.5674.3 | 0.947327 | 0.260308 | 2 |

| RNA processing and modification | Gene ID | Isoforms ID | FDR | Log_2_FC | Classify |
| --- | --- | --- | --- | --- | --- |
| polypyrimidine tract-binding protein 2 | evm.TU.scaffold_315.12 | PB.8556.1 |  |  | 2 |
|  |  | PB.8556.2 | 0.792055 | -0.31115 | 2 |
|  |  | **PB.8556.3** | **7.81E-05** | **6.338209** | **2** |
|  |  | PB.8556.4 | 0.844668 | 0.570971 | 2 |
|  |  |  |  |  |  |
| THO complex subunit 4 | evm.TU.scaffold_156.19 | PB.3607.1 | 0.976995 | -0.05139 | 1 |
|  |  | PB.3605.1 | 0.956818 | -0.09043 | 2 |
|  |  | PB.3606.1 | 0.481427 | -0.36842 | 2 |
|  |  | PB.3606.2 | 0.969936 | -0.05834 | 2 |
|  |  | **PB.3606.3** | **1.72E-05** | **-4.64825** | **2** |
|  |  | PB.3607.2 | 0.240508 | 3.205038 | 2 |
|  |  | PB.3607.3 | 0.966231 | 0.069984 | 2 |
|  |  |  |  |  |  |
| ELL-associated factor 1 | evm.TU.scaffold_14.185 | PB.2959.1 | 0.083619 | 2.110141 | 2 |
|  |  | PB.2959.2 |  |  | 2 |
|  |  | **PB.2959.3** | **0.008792** | **-3.9115** | **2** |
|  |  |  |  |  |  |
| putative ATP-dependent RNA helicase DHX57 | evm.TU.scaffold_120.45 | PB.1943.1 | 0.382899 | 0.893142 | 2 |
|  |  | PB.1943.2 | 0.43041 | 0.752949 | 2 |
|  |  | PB.1943.3 | 0.983147 | -0.13634 | 2 |
|  |  | **PB.1943.4** | **0.022114** | **-3.75352** | **2** |
|  |  |  |  |  |  |
| SAFB-like transcription modulator | evm.TU.scaffold_30.50 | PB.8255.1 |  |  | 2 |
|  |  | PB.8255.2 |  |  | 2 |
|  |  | PB.8255.3 | 0.93987 | 0.338705 | 2 |
|  |  | **PB.8255.4** | **6.7E-05** | **-3.50317** | **2** |
|  |  | PB.8255.5 | 0.968611 | -0.08764 | 2 |
|  |  |  |  |  |  |
| nuclear pore membrane glycoprotein 210 | evm.TU.scaffold_11.62 | PB.1236.1 | 0.227495 | 0.636052 | 1 |
|  |  | **PB.1236.2** | **0.017746** | **-3.39842** | **2** |
|  |  |  |  |  |  |
| nucleoporin p58/p45 | evm.TU.scaffold_106.42 | PB.1068.1 | 0.631931 | 0.80005 | 2 |
|  |  | **PB.1068.2** | **0.000954** | **-3.2672** | **2** |
|  |  | PB.1068.3 | 0.976722 | -0.15945 | 2 |
|  |  |  |  |  |  |
| polyadenylate-binding protein 1 | evm.TU.scaffold_34.116 | PB.9001.18 | 0.970184 | -0.06353 | 1 |
|  |  | PB.9001.1 |  |  | 2 |
|  |  | PB.9001.10 | 0.491771 | 0.368736 | 2 |
|  |  | PB.9001.11 |  |  | 2 |
|  |  | PB.9001.12 |  |  | 2 |
|  |  | PB.9001.13 |  |  | 2 |
|  |  | PB.9001.14 |  |  | 2 |
|  |  | PB.9001.15 |  |  | 2 |
|  |  | PB.9001.16 | 0.805155 | -0.97923 | 2 |
|  |  | PB.9001.17 | 0.950834 | -0.3516 | 2 |
|  |  | **PB.9001.19** | **0.019376** | **-3.10533** | **2** |
|  |  | PB.9001.2 | 0.88401 | 0.705129 | 2 |
|  |  | PB.9001.3 | 0.976289 | -0.0532 | 2 |
|  |  | PB.9001.5 | 0.575088 | 0.822606 | 2 |
|  |  | PB.9001.6 |  |  | 2 |
|  |  | PB.9001.7 |  |  | 2 |
|  |  | PB.9001.8 | 0.848833 | 0.196113 | 2 |
|  |  | PB.9001.9 |  |  | 2 |
|  |  |  |  |  |  |
| inosine-5&apos;-monophosphate dehydrogenase 1b | evm.TU.scaffold_2.218_evm.TU.scaffold_2.219 | PB.5223.1 | 0.981572 | 0.064822 | 2 |
|  |  | PB.5223.2 | 0.975915 | 0.407772 | 2 |
|  |  | **PB.5223.3** | **0.00069** | **-3.00316** | **2** |
|  |  |  |  |  |  |
| exosome complex exonuclease RRP44 | evm.TU.scaffold_42.120 | PB.10294.2 | 0.266302 | 0.858968 | 1 |
|  |  | **PB.10294.1** | **0.032693** | **-2.87314** | **2** |
|  |  |  |  |  |  |
| cytidine deaminase | evm.TU.scaffold_226.10 | PB.6084.1 | 0.002701 | -2.971 | 1 |
|  |  | **PB.6084.2** | **4.35E-12** | **-2.76292** | **2** |
|  |  |  |  |  |  |
| RNA-binding protein 47 | evm.TU.scaffold_15.199 | PB.3425.1 | 1 | -0.0042 | 2 |
|  |  | **PB.3425.2** | **0.037279** | **2.761017** | **2** |
|  |  | PB.3425.3 | 0.061571 | 0.92165 | 2 |
|  |  | PB.3425.5 |  |  | 2 |
|  |  | PB.3425.6 | 0.899517 | 0.416466 | 2 |
|  |  |  |  |  |  |
| pre-mRNA-processing factor 39 | evm.TU.scaffold_1.98 | **PB.341.12** | **0.000106** | **2.675008** | **1** |
|  |  | **PB.341.2** | **0.000106** | **2.675008** | **1** |
|  |  | PB.341.1 |  |  | 2 |
|  |  | PB.341.10 |  |  | 2 |
|  |  | PB.341.11 | 0.963716 | 0.091801 | 2 |
|  |  | PB.341.13 |  |  | 2 |
|  |  |  |  |  |  |
| elongation factor 1-alpha | PB.10145 | PB.10145.1 | 0.971965 | 0.065789 | 3 |
|  |  | PB.10145.2 | 0.919787 | 0.206286 | 3 |
|  |  | PB.10145.3 | 0.631009 | 0.697115 | 3 |
|  |  | **PB.10145.4** | **0.004913** | **-2.63876** | **3** |
|  |  |  |  |  |  |
| amyloid beta A4 protein | evm.TU.scaffold_46.45 | **PB.10587.1** | **0.014347** | **2.018862** | **2** |
|  |  | PB.10587.10 | 0.977332 | 0.074458 | 2 |
|  |  | PB.10587.2 |  |  | 2 |
|  |  | PB.10587.3 | 0.981572 | -0.08186 | 2 |
|  |  | PB.10587.5 | 0.264716 | 1.668143 | 2 |
|  |  | PB.10587.6 | 0.998603 | 0.070847 | 2 |
|  |  | PB.10587.7 |  |  | 2 |
|  |  | PB.10587.8 |  |  | 2 |
|  |  | PB.10587.9 | 0.967493 | 0.078202 | 2 |
|  |  |  |  |  |  |
| nucleolin 2-like | evm.TU.scaffold_41.58 | PB.10153.3 | 0.437739 | 0.579079 | 1 |
|  |  | PB.10153.1 | 0.596045 | 0.79261 | 2 |
|  |  | PB.10153.2 | 0.979223 | 0.154709 | 2 |
|  |  | **PB.10153.4** | **0.000822** | **-1.82073** | **2** |
|  |  | PB.10153.5 | 0.779487 | -0.76245 | 2 |
|  |  | PB.10153.6 | 1.61E-05 |  | 2 |
|  |  | PB.10153.7 | 0.892261 | 0.409751 | 2 |
|  |  |  |  |  |  |
| heterogeneous nuclear ribonucleoprotein M | evm.TU.scaffold_9.41 | PB.14321.1 | 0.442235 | 0.393709 | 2 |
|  |  | PB.14321.2 | 0.850303 | 0.179985 | 2 |
|  |  | PB.14321.3 | 0.892261 | 0.677632 | 2 |
|  |  | PB.14321.4 | 0.882756 | -0.32916 | 2 |
|  |  | **PB.14321.5** | **3.69E-07** | **-1.63893** | **2** |
|  |  |  |  |  |  |
| ran GTPase-activating protein 1 | evm.TU.scaffold_116.12 | **PB.1622.1** | **0.046889** | **1.247494** | **1** |
|  |  | PB.1622.2 |  |  | 2 |
|  |  | PB.1622.3 | 0.705198 | 1.609204 | 2 |
|  |  | PB.1622.4 |  |  | 2 |
|  |  | **PB.1622.5** | **7.02E-08** | **-1.5684** | **2** |
|  |  |  |  |  |  |
| pre-mRNA-processing factor 39 | evm.TU.scaffold_1.98 | PB.341.14 | 0.15671 | -0.57711 | 2 |
|  |  | PB.341.3 | 0.721091 | -0.43874 | 2 |
|  |  | PB.341.4 | 1 | 0.005579 | 2 |
|  |  | **PB.341.5** | **0.039993** | **1.530159** | **2** |
|  |  | PB.341.6 |  |  | 2 |
|  |  | PB.341.7 |  |  | 2 |
|  |  | PB.341.8 | 0.497387 | -0.38301 | 2 |
|  |  | PB.341.9 | 0.781697 | 0.97503 | 2 |
|  |  |  |  |  |  |
| protein argonaute-2 | evm.TU.scaffold_60.102 | **PB.12044.1** | **0.02266** | **1.501499** | **1** |
|  |  |  |  |  |  |
| heterogeneous nuclear ribonucleoprotein U-like protein 1 | evm.TU.scaffold_16.118 | PB.3784.3 | 0.492829 | 0.35937 | 1 |
|  |  | PB.3784.1 |  |  | 2 |
|  |  | PB.3784.4 | 1 | -0.07144 | 2 |
|  |  | **PB.3784.5** | **0.035665** | **-1.43094** | **2** |
|  |  |  |  |  |  |
| poly(ADP-ribose) polymerase 14-like | evm.TU.scaffold_199.56 | PB.5082.1 | 0.001071 | -1.24614 | 1 |
|  |  |  |  |  |  |
| RNA-binding protein 5 | evm.TU.scaffold_76.86 | PB.13265.1 | 0.696357 | -0.2661 | 2 |
|  |  | PB.13265.2 | 0.604612 | -0.46441 | 2 |
|  |  | **PB.13266.1** | **0.000993** | **1.343588** | **2** |
|  |  | PB.13266.2 | 0.721525 | 2.384431 | 2 |
|  |  | PB.13266.3 | 0.071881 | -4.0242 | 2 |
|  |  | PB.13266.4 | 0.981039 | 0.049501 | 2 |
|  |  | PB.13266.5 | 0.964449 | -0.07253 | 2 |
|  |  | PB.13266.6 | 0.557328 | -0.79885 | 2 |
|  |  | PB.13266.7 |  |  | 2 |
|  |  | PB.13266.8 |  |  | 2 |
|  |  | PB.13266.9 | 0.913568 | 0.123946 | 2 |
|  |  |  |  |  |  |
| probable ATP-dependent RNA helicase DDX5 | evm.TU.scaffold_136.57 | PB.2712.8 | 0.965985 | -0.12076 | 1 |
|  |  | PB.2712.1 |  |  | 2 |
|  |  | **PB.2712.10** | **2.35E-05** | **-1.17639** | **2** |
|  |  | PB.2712.2 | 0.823476 | -0.19543 | 2 |
|  |  | PB.2712.3 | 0.935663 | 0.157702 | 2 |
|  |  | PB.2712.6 | 1 | -0.0081 | 2 |
|  |  | PB.2712.7 |  |  | 2 |
|  |  |  |  |  |  |
| serine/arginine-rich splicing factor 7 | evm.TU.scaffold_16.35 | PB.3736.4 | 0.052062 | -1.65771 | 1 |
|  |  | PB.3736.1 | 0.856931 | 0.178743 | 2 |
|  |  | PB.3736.2 | 0.755907 | -0.26286 | 2 |
|  |  | PB.3736.3 | 0.96793 | -0.30436 | 2 |
|  |  | PB.3736.5 | 0.474702 | 0.607767 | 2 |
|  |  | **PB.3736.6** | **0.043042** | **1.122162** | **2** |
|  |  | PB.3736.7 |  |  | 2 |
|  |  |  |  |  |  |
| trinucleotide repeat-containing gene 18 protein | evm.TU.scaffold_264.9 | PB.7268.1 | 0.039827 | 1.287521 | 1 |
|  |  |  |  |  |  |
| Y&apos; element ATP-dependent helicase YEL077C | evm.TU.scaffold_59.28 | PB.11739.1 | 0.365731 | 0.658253 | 2 |
|  |  | **PB.11739.2** | **0.001985** | **1.07145** | **2** |
|  |  | PB.11739.3 | 0.990694 | -0.05377 | 2 |
|  |  | PB.11739.4 | 0.000873 | 0.988761 | 2 |
|  |  |  |  |  |  |
| bromodomain-containing protein 4 | evm.TU.scaffold_94.9 | **PB.14781.1** | **0.002339** | **1.004393** | **1** |
|  |  |  |  |  |  |
| RNA-binding protein 39 | evm.TU.scaffold_11.7 | PB.1212.1 | 0.944404 | -0.35719 | 2 |
|  |  | PB.1212.10 | 0.134903 | -1.75359 | 2 |
|  |  | PB.1212.11 | 0.697568 | -0.65246 | 2 |
|  |  | PB.1212.12 | 0.836831 | -1.31686 | 2 |
|  |  | PB.1212.13 | 0.318118 | -2.54078 | 2 |
|  |  | PB.1212.14 | 0.70661 | -0.42833 | 2 |
|  |  | PB.1212.15 | 0.597288 | 0.776876 | 2 |
|  |  | PB.1212.16 | 0.722696 | 0.797954 | 2 |
|  |  | PB.1212.2 | 0.928066 | 0.21708 | 2 |
|  |  | PB.1212.3 |  |  | 2 |
|  |  | **PB.1212.4** | **0.027704** | **inf** | **2** |
|  |  | PB.1212.5 | 0.310343 | -0.46197 | 2 |
|  |  | PB.1212.6 |  |  | 2 |
|  |  | PB.1212.7 |  |  | 2 |
|  |  | PB.1212.8 |  |  | 2 |
|  |  | PB.1212.9 |  |  | 2 |
|  |  |  |  |  |  |
| ataxin-1 | evm.TU.scaffold_11.82 | PB.1249.1 | 0.712956 | -0.51806 | 1 |
|  |  | **PB.1249.2** | **0.008424** | **inf** | **2** |
|  |  |  |  |  |  |
| Protein quaking-B | evm.TU.scaffold_119.66 | **PB.1775.1** | **2.45E-05** | **inf** | **2** |
|  |  | PB.1775.2 | 0.842955 | 0.803046 | 2 |
|  |  | PB.1775.3 |  |  | 2 |
|  |  | PB.1775.4 |  |  | 2 |
|  |  |  |  |  |  |
| THAP domain-containing protein 5 | evm.TU.scaffold_225.12 | PB.6071.1 | 0.907552 | -0.36866 | 1 |
|  |  | **PB.6071.2** | **5.86E-05** | **inf** | **2** |
|  |  |  |  |  |  |
| putative ribosomal RNA methyltransferase 2 | evm.TU.scaffold_23.195 | PB.6267.1 |  |  | 2 |
|  |  | **PB.6267.2** | **0.000129** | **inf** | **2** |
|  |  |  |  |  |  |
| RNA polymerase-associated protein CTR9 homolog | evm.TU.scaffold_30.8 | PB.8227.1 | 0.904462 | 0.161602 | 2 |
|  |  | PB.8227.2 |  |  | 2 |
|  |  | **PB.8227.3** | **9.08E-12** | **inf** | **2** |
|  |  | PB.8227.4 |  |  | 2 |
|  |  |  |  |  |  |
| RNA-binding protein 33 | evm.TU.scaffold_7.229 | PB.12704.2 | 0.282691 | 0.458867 | 1 |
|  |  | **PB.12704.1** | **2.13E-11** | **inf** | **2** |

| Signal Transduction | Gene ID | Isoforms ID | FDR | Log_2_FC | Classify |
| --- | --- | --- | --- | --- | --- |
| protein Mdm4 | evm.TU.scaffold_226.38 | PB.6100.1 | 0.98755 | -0.03151 | 2 |
|  |  | PB.6100.2 | 0.423031 | 0.576087 | 2 |
|  |  | **PB.6100.3** | **0.000125** | **-4.94982** | **2** |
|  |  |  |  |  |  |
| tyrosine-protein kinase Fyn-like isoform 1 | evm.TU.scaffold_108.5 | PB.1111.1 |  |  | 2 |
|  |  | PB.1111.2 | 0.100914 | 0.919104 | 2 |
|  |  | **PB.1111.3** | **0.000139** | **4.645873** | **2** |
|  |  | PB.1111.4 | 0.960907 | 0.258703 | 2 |
|  |  | PB.1111.5 | 0.062216 | -2.02572 | 2 |
|  |  | PB.1111.6 |  |  | 2 |
|  |  | PB.1111.7 | 0.914947 | -1.16486 | 2 |
|  |  |  |  |  |  |
| cAMP-dependent protein kinase type II-alpha regulatory subunit | evm.TU.scaffold_175.32 | PB.4330.1 | 0.67329 | 0.279132 | 2 |
|  |  | PB.4330.3 | 0.773287 | 0.657357 | 2 |
|  |  | PB.4330.4 |  |  | 2 |
|  |  | **PB.4330.5** | **0.024078** | **-4.59795** | **2** |
|  |  | PB.4330.6 |  |  | 2 |
|  |  |  |  |  |  |
| pleckstrin homology domain-containing family H member 1 | evm.TU.scaffold_79.52 | PB.13444.1 | 0.994097 | 0.151463 | 2 |
|  |  | **PB.13444.2** | **0.002196** | **-4.29343** | **2** |
|  |  |  |  |  |  |
| zinc finger CCCH domain-containing protein 7A | evm.TU.scaffold_23.209 | **PB.6273.3** | **0.000147** | **4.247072** | **1** |
|  |  | PB.6273.1 | 0.882373 | -0.15863 | 2 |
|  |  | PB.6273.2 |  |  | 2 |
|  |  |  |  |  |  |
| postacrosomal sheath WW domain-binding protein | evm.TU.scaffold_136.31 | PB.2700.6 | 0.687691 | 0.272869 | 1 |
|  |  | PB.2700.1 | 0.687691 | 0.272869 | 1 |
|  |  | PB.2700.2 | 0.05411 | -2.75249 | 2 |
|  |  | PB.2700.3 | 0.348939 | -0.69706 | 2 |
|  |  | **PB.2700.4** | **0.000669** | **-4.15565** | **2** |
|  |  | PB.2700.5 |  |  | 2 |
|  |  |  |  |  |  |
| Guanine nucleotide exchange factor VAV2 | evm.TU.scaffold_31.89 | PB.8460.1 |  |  | 2 |
|  |  | PB.8460.2 |  |  | 2 |
|  |  | PB.8460.3 | 0.914522 | 0.40199 | 2 |
|  |  | PB.8460.5 |  |  | 2 |
|  |  | **PB.8460.6** | **0.028869** | **4.00264** | **2** |
|  |  | PB.8460.7 |  |  | 2 |
|  |  | PB.8460.8 |  |  | 2 |
|  |  |  |  |  |  |
| ceramide synthase 2 | evm.TU.scaffold_240.42 | PB.6694.1 | 0.890442 | 0.270167 | 1 |
|  |  | **PB.6694.2** | **0.014349** | **3.90944** | **2** |
|  |  | PB.6694.3 |  |  | 2 |
|  |  | PB.6694.4 |  |  | 2 |
|  |  |  |  |  |  |
| signal peptide peptidase-like 2B | evm.TU.scaffold_59.132 | **PB.11802.4** | **0.000227** | **-3.67819** | **1** |
|  |  | PB.11802.2 | 0.825955 | 0.428362 | 2 |
|  |  | PB.11802.3 |  |  | 2 |
|  |  | PB.11802.5 | 0.989967 | -0.13616 | 2 |
|  |  | PB.11802.6 | 0.315869 | 2.095941 | 2 |
|  |  |  |  |  |  |
| MAPK/MAK/MRK overlapping kinase | evm.TU.scaffold_80.12 | **PB.13693.1** | **0.039827** | **-3.55865** | **2** |
|  |  | **PB.13693.2** | **7.81E-05** | **lnf** | **2** |
|  |  |  |  |  |  |
|  |  |  |  |  |  |
| copine-3 | evm.TU.scaffold_110.8 | PB.1428.1 | 0.842576 | -0.33945 | 1 |
|  |  | **PB.1428.2** | **0.019284** | **3.330208** | **2** |
|  |  |  |  |  |  |
| adenosine receptor A2b | evm.TU.scaffold_140.37 | **PB.3017.1** | **0.047217** | **3.141828** | **2** |
|  |  | PB.3017.2 | 0.878176 | -0.55954 | 2 |
|  |  |  |  |  |  |
| signal recognition particle subunit SRP68 | evm.TU.scaffold_301.7 | PB.8330.1 |  |  | 2 |
|  |  | **PB.8330.2** | **1.3E-07** | **3.124314** | **2** |
|  |  | PB.8330.3 | 0.134903 | -0.73957 | 2 |
|  |  | PB.8330.4 | 0.668525 | 0.594761 | 2 |
|  |  |  |  |  |  |
| pleckstrin homology domain-containing family G member 5 | evm.TU.scaffold_11.319 | PB.1410.3 | 0.113261 | 0.837114 | 1 |
|  |  | PB.1410.1 | 0.958606 | 0.137788 | 2 |
|  |  | **PB.1410.2** | **0.036635** | **3.007004** | **2** |
|  |  |  |  |  |  |
| serine/threonine-protein kinase B-raf | evm.TU.scaffold_206.10 | **PB.5479.1** | **0.047066** | **2.737672** | **2** |
|  |  |  |  |  |  |
| mitogen-activated protein kinase kinase kinase 14 | evm.TU.scaffold_23.187 | PB.6260.10 | 0.890442 | 0.266841 | 1 |
|  |  | PB.6260.6 | 0.890442 | 0.266841 | 1 |
|  |  | PB.6260.1 | 0.181042 | -0.99264 | 2 |
|  |  | PB.6260.11 | 0.9271 | -0.36857 | 2 |
|  |  | PB.6260.2 | 0.076301 | 2.636955 | 2 |
|  |  | PB.6260.3 |  |  | 2 |
|  |  | PB.6260.4 | 0.71997 | -1.33051 | 2 |
|  |  | PB.6260.5 | 0.857522 | -0.59735 | 2 |
|  |  | PB.6260.7 |  |  | 2 |
|  |  | **PB.6260.8** | **0.048873** | **-2.48582** | **2** |
|  |  | PB.6260.9 |  |  | 2 |
|  |  |  |  |  |  |
| arrestin domain-containing protein 3 | evm.TU.scaffold_0.494 | PB.271.1 | 0.986828 | 0.033885 | 2 |
|  |  | PB.271.2 | 0.861437 | -0.85278 | 2 |
|  |  | PB.271.4 |  |  | 2 |
|  |  | **PB.271.5** | **0.023208** | **2.45827** | **2** |
|  |  | PB.271.6 |  |  | 2 |
|  |  | PB.271.7 |  |  | 2 |
|  |  | PB.271.8 |  |  | 2 |
|  |  | PB.271.9 |  |  | 2 |
|  |  |  |  |  |  |
| serine/threonine-protein kinase tousled-like 1-B | evm.TU.scaffold_217.10 | PB.5854.1 | 0.983147 | 0.118113 | 1 |
|  |  | PB.5854.2 | 0.392203 | -1.20914 | 2 |
|  |  | PB.5854.3 | 0.947791 | -0.10901 | 2 |
|  |  | **PB.5854.4** | **0.001141** | **2.337487** | **2** |
|  |  |  |  |  |  |
| ras GTPase-activating-like protein IQGAP2 | evm.TU.scaffold_3.401 | PB.8213.2 | 0.962594 | 0.072824 | 1 |
|  |  | PB.8212.1 |  |  | 2 |
|  |  | PB.8212.3 |  |  | 2 |
|  |  | PB.8212.4 |  |  | 2 |
|  |  | PB.8212.5 |  |  | 2 |
|  |  | PB.8213.1 |  |  | 2 |
|  |  | **PB.8213.3** | **0.043719** | **-2.16169** | **2** |
|  |  |  |  |  |  |
| tyrosine-protein phosphatase non-receptor type 22 | evm.TU.scaffold_11.288 | PB.1389.1 | 0.889862 | -0.5948 | 2 |
|  |  | PB.1389.2 | 0.387897 | 0.961328 | 2 |
|  |  | **PB.1389.3** | **0.002646** | **1.930224** | **2** |
|  |  | PB.1389.4 |  |  | 2 |
|  |  | PB.1389.5 | 0.970726 | -0.24336 | 2 |
|  |  | PB.1389.6 | 0.114341 | -1.92853 | 2 |
|  |  |  |  |  |  |
| regulator of G-protein signaling 4 | evm.TU.scaffold_38.7 | **PB.9499.1** | **3.71E-07** | **-1.79692** | **1** |
|  |  |  |  |  |  |
| dual specificity tyrosine-phosphorylation-regulated kinase 1A | evm.TU.scaffold_92.85 | **PB.14710.1** | **0.005757** | **-1.76553** | **2** |
|  |  |  |  |  |  |
| pleckstrin homology domain-containing family A member 1 | evm.TU.scaffold_42.84 | PB.10273.1 | 0.922361 | -0.16409 | 2 |
|  |  | PB.10273.2 | 0.302753 | 1.374421 | 2 |
|  |  | **PB.10273.3** | **3.07E-09** | **-1.71027** | **2** |
|  |  |  |  |  |  |
| casein kinase I isoform epsilon | evm.TU.scaffold_136.6 | PB.2686.2 | 0.190846 | 1.358405 | 1 |
|  |  | PB.2686.1 | 0.758721 | -0.56063 | 2 |
|  |  | PB.2686.3 | 0.965985 | 0.086764 | 2 |
|  |  | **PB.2686.4** | **0.024386** | **-1.6684** | **2** |
|  |  |  |  |  |  |
| signal peptidase complex subunit 2 | evm.TU.scaffold_100.83 | PB.800.1 |  |  | 2 |
|  |  | **PB.800.2** | **0.002384** | **-1.54764** | **2** |
|  |  | PB.800.3 | 0.072474 | 0.6176 | 2 |
|  |  | PB.800.4 | 0.990455 | 0.080065 | 2 |
|  |  | PB.800.5 | 0.185082 | -2.00105 | 2 |
|  |  |  |  |  |  |
| prolactin receptor | evm.TU.scaffold_37.107 | **PB.9419.1** | **0.001141** | **-1.45761** | **1** |
|  |  |  |  |  |  |
| lysophosphatidic acid receptor 2 | evm.TU.scaffold_94.10 | PB.14782.3 | 0.98886 | 0.028699 | 1 |
|  |  | PB.14782.1 | 0.987186 | 0.03218 | 2 |
|  |  | **PB.14782.2** | **0.000999** | **-1.44762** | **2** |
|  |  | PB.14782.4 | 0.785848 | 1.904519 | 2 |
|  |  | PB.14782.5 | 0.375343 | 1.820029 | 2 |
|  |  |  |  |  |  |
| pleckstrin homology domain-containing family S member 1 | evm.TU.scaffold_4.162 | PB.9812.3 |  |  | 1 |
|  |  | PB.9812.1 | 0.523976 | 0.949632 | 2 |
|  |  | **PB.9812.2** | **4.5E-08** | **1.381315** | **2** |
|  |  |  |  |  |  |
| cryptochrome-1 | evm.TU.scaffold_141.32 | **PB.3041.1** | **0.032776** | **1.360211** | **2** |
|  |  |  |  |  |  |
| integrin-linked protein kinase | evm.TU.scaffold_203.5 | PB.5418.1 | 0.196154 | 0.562234 | 1 |
|  |  | **PB.5418.2** | **0.002384** | **-1.31878** | **2** |
|  |  |  |  |  |  |
| tetraspanin-8 | evm.TU.scaffold_391.3 | PB.9706.1 | 0.634149 | 0.29742 | 2 |
|  |  | PB.9706.10 | 0.984592 | 0.130535 | 2 |
|  |  | PB.9706.12 | 0.642179 | 1.965978 | 2 |
|  |  | PB.9706.13 | 0.487921 | 1.574909 | 2 |
|  |  | PB.9706.14 | 0.957 | -0.23971 | 2 |
|  |  | PB.9706.16 | 0.987467 | 0.065235 | 2 |
|  |  | **PB.9706.2** | **0.000123** | **-1.28043** | **2** |
|  |  | PB.9706.3 | 0.705016 | -0.28326 | 2 |
|  |  | PB.9706.4 | 0.688228 | 0.419086 | 2 |
|  |  | PB.9706.5 | 0.829246 | -3.05198 | 2 |
|  |  | PB.9706.6 | 0.905986 | 0.18881 | 2 |
|  |  | PB.9706.7 | 0.893415 | 0.276544 | 2 |
|  |  | PB.9706.9 |  |  | 2 |
|  |  |  |  |  |  |
| rho GTPase-activating protein 35 | evm.TU.scaffold_56.94 | **PB.11509.2** | **0.004448** | **1.183096** | **1** |
|  |  | **PB.11509.4** | **0.004448** | **1.183096** | **1** |
|  |  | PB.11509.1 | 0.816185 | -0.24104 | 2 |
|  |  | PB.11509.3 |  |  | 2 |
|  |  | PB.11509.5 |  |  | 2 |
|  |  |  |  |  |  |
| calcineurin B homologous protein 1 | evm.TU.scaffold_236.26 | **PB.6421.1** | **1.72E-05** | **-1.13427** | **1** |
|  |  |  |  |  |  |
| EF-hand calcium-binding domain-containing protein 4B | evm.TU.scaffold_35.73 | **PB.9109.2** | **0.018173** | **1.132593** | **1** |
|  |  | **PB.9109.4** | **0.018173** | **1.132593** | **1** |
|  |  | PB.9109.5 |  |  | 2 |
|  |  |  |  |  |  |
| protein kinase C delta type | evm.TU.scaffold_44.48 | PB.10417.1 | 0.977258 | -0.04786 | 1 |
|  |  | **PB.10417.2** | **0.017361** | **-1.11659** | **2** |
|  |  |  |  |  |  |
| protein Niban | evm.TU.scaffold_96.34 | **PB.14900.1** | **0.002537** | **1.116269** | **1** |
|  |  | PB.14900.2 | 0.852474 | 1.419719 | 2 |
|  |  |  |  |  |  |
| transforming protein RhoA | evm.TU.scaffold_165.29 | **PB.3969.3** | **0.019248** | **1.100176** | **1** |
|  |  | PB.3969.1 | 0.970685 | 0.058223 | 2 |
|  |  | PB.3969.2 | 0.627807 | -0.31563 | 2 |
|  |  |  |  |  |  |
| rap guanine nucleotide exchange factor 6 | evm.TU.scaffold_192.10 | **PB.4916.1** | **0.013167** | **1.031513** | **1** |
|  |  |  |  |  |  |
| rho GTPase-activating protein 8 | evm.TU.scaffold_152.71 | **PB.3509.1** | **0.018173** | **-1.00327** | **2** |
|  |  | PB.3509.2 | 0.187452 | 1.020046 | 2 |
|  |  |  |  |  |  |
| ellis-van Creveld syndrome protein | evm.TU.scaffold_126.36 | PB.2136.1 | 0.199102 | 1.878864 | 2 |
|  |  | **PB.2136.2** | **0.000213** | **inf** | **2** |
|  |  |  |  |  |  |
| ceramide synthase 3 | evm.TU.scaffold_163.19 | PB.3919.1 | 0.994097 | -0.02336 | 2 |
|  |  | PB.3919.2 | 5.46E-06 | inf | 2 |
|  |  | PB.3919.3 | 0.957171 | -0.38701 | 2 |
|  |  |  |  |  |  |
| insulin-like growth factor 1 | evm.TU.scaffold_2.116 | **PB.5155.2** | **2.78E-05** | **Inf** | **1** |
|  |  | PB.5155.1 | 0.941951 | -0.39705 | 2 |
|  |  | PB.5155.3 |  |  | 2 |
|  |  | PB.5155.4 | 0.86429 | 1.20051 | 2 |
|  |  | PB.5155.5 |  |  | 2 |
|  |  |  |  |  |  |
| calcium/calmodulin-dependent protein kinase type 1 | evm.TU.scaffold_226.30 | PB.6096.1 |  |  | 2 |
|  |  | PB.6096.2 | 0.590605 | 0.413784 | 2 |
|  |  | **PB.6096.3** | **0.039827** | **inf** | **2** |
|  |  | PB.6096.4 | 1 | 0.03444 | 2 |
|  |  |  |  |  |  |
| membrane progestin receptor alpha-B | evm.TU.scaffold_234.5 | PB.6383.1 | 0.427271 | -0.71773 | 2 |
|  |  | PB.6383.2 | 0.75522 | -0.51701 | 2 |
|  |  | PB.6383.3 |  |  | 2 |
|  |  | PB.6383.4 | 0.994541 | 0.066295 | 2 |
|  |  | **PB.6383.5** | **2.67E-05** | **inf** | **2** |
|  |  |  |  |  |  |
| LIM and senescent cell antigen-like-containing domain protein 1 | evm.TU.scaffold_25.10 | **PB.6832.1** | **0.000201** | **inf** | **2** |
|  |  | PB.6832.2 | 0.282691 | -0.70443 | 2 |
|  |  | PB.6832.3 |  |  | 2 |
|  |  | PB.6832.4 | 0.811724 | 1.319418 | 2 |
|  |  | PB.6832.5 |  |  | 2 |
|  |  |  |  |  |  |
| A-kinase anchor protein 10, mitochondrial | evm.TU.scaffold_26.211 | **PB.7211.1** | **1.87E-06** | **inf** | **2** |
|  |  | PB.7211.2 |  |  | 2 |
|  |  |  |  |  |  |
| TNFAIP3-interacting protein 1 | evm.TU.scaffold_27.176 | PB.7450.19 | 0.906437 | 0.186955 | 1 |
|  |  | PB.7450.10 | 0.850303 | 0.808034 | 2 |
|  |  | PB.7450.11 |  |  | 2 |
|  |  | PB.7450.12 |  |  | 2 |
|  |  | PB.7450.13 | 0.968162 | -0.18547 | 2 |
|  |  | PB.7450.14 | 0.986828 | 0.032939 | 2 |
|  |  | PB.7450.16 |  |  | 2 |
|  |  | PB.7450.17 |  |  | 2 |
|  |  | PB.7450.18 |  |  | 2 |
|  |  | PB.7450.2 |  |  | 2 |
|  |  | PB.7450.20 | 0.914416 | 0.696867 | 2 |
|  |  | PB.7450.21 |  |  | 2 |
|  |  | PB.7450.22 | 0.976244 | -0.46819 | 2 |
|  |  | PB.7450.23 |  |  | 2 |
|  |  | PB.7450.24 | 0.783381 | 1.532061 | 2 |
|  |  | PB.7450.26 |  |  | 2 |
|  |  | PB.7450.27 | 1 | 0.104808 | 2 |
|  |  | PB.7450.28 |  |  | 2 |
|  |  | PB.7450.29 |  |  | 2 |
|  |  | PB.7450.3 |  |  | 2 |
|  |  | PB.7450.30 |  |  | 2 |
|  |  | PB.7450.31 |  |  | 2 |
|  |  | PB.7450.32 | 0.758721 | -1.31896 | 2 |
|  |  | PB.7450.33 |  |  | 2 |
|  |  | PB.7450.34 | 0.866743 | 1.266302 | 2 |
|  |  | PB.7450.35 | 0.557742 | 1.556973 | 2 |
|  |  | PB.7450.36 |  |  | 2 |
|  |  | PB.7450.37 |  |  | 2 |
|  |  | **PB.7450.38** | **0.000106** | **inf** | **2** |
|  |  | PB.7450.39 |  |  | 2 |
|  |  | PB.7450.4 |  |  | 2 |
|  |  | PB.7450.40 |  |  | 2 |
|  |  | PB.7450.41 | 0.903973 | -0.8695 | 2 |
|  |  | PB.7450.42 | 0.810547 | -0.60434 | 2 |
|  |  | PB.7450.43 |  |  | 2 |
|  |  | PB.7450.44 |  |  | 2 |
|  |  | PB.7450.5 | 0.994097 | 0.068159 | 2 |
|  |  | PB.7450.6 | 0.495114 | -0.60971 | 2 |
|  |  | PB.7450.7 |  |  | 2 |
|  |  | PB.7450.8 | 0.829246 | -1.04731 | 2 |
|  |  | PB.7450.9 |  |  | 2 |
|  |  |  |  |  |  |
| lysosome membrane protein 2 | evm.TU.scaffold_37.76 | PB.9404.2 | 0.184557 | 1.227008 | 1 |
|  |  | PB.9404.1 |  |  | 2 |
|  |  | PB.9404.3 | 0.930529 | 0.12531 | 2 |
|  |  | PB.9404.4 |  |  | 2 |
|  |  | PB.9404.5 |  |  | 2 |
|  |  | PB.9404.6 | 0.906112 | -0.94938 | 2 |
|  |  | PB.9404.7 |  |  | 2 |
|  |  | PB.9404.8 | 0.544579 | -2.39301 | 2 |
|  |  | **PB.9404.9** | **3.3E-06** | **inf** | **2** |
|  |  |  |  |  |  |
| tyrosine kinase 2 | evm.TU.scaffold_40.12 | PB.9954.1 | 0.600965 | 0.313506 | 1 |
|  |  | PB.9954.2 | 0.681187 | -1.19949 | 2 |
|  |  | PB.9954.3 | 0.44819 | 1.340252 | 2 |
|  |  | PB.9954.5 |  |  | 2 |
|  |  | PB.9954.6 | 0.910865 | -0.604 | 2 |
|  |  | **PB.9954.7** | **0.001228** | **inf** | **2** |
|  |  | PB.9954.8 |  |  | 2 |
|  |  | PB.9954.9 |  |  | 2 |
|  |  |  |  |  |  |
| tsukushin | evm.TU.scaffold_49.79 | PB.10837.1 | 1 | -0.0039 | 2 |
|  |  | PB.10837.2 |  |  | 2 |
|  |  | **PB.10837.3** | **8.71E-07** | **inf** | **2** |
|  |  | PB.10837.4 | 0.856883 | -0.3704 | 2 |
|  |  | PB.10837.5 |  |  | 2 |
|  |  |  |  |  |  |
| serine/threonine-protein kinase Sgk2 | evm.TU.scaffold_5.19 | PB.10899.1 | 0.000454 | -0.95543 | 1 |
|  |  | **PB.10899.2** | **2E-05** | **inf** | **2** |
|  |  |  |  |  |  |
| type III endosome membrane protein TEMP | evm.TU.scaffold_77.25 | PB.13326.1 | 0.04968 | -0.72534 | 1 |
|  |  | PB.13326.2 |  |  | 2 |
|  |  | **PB.13326.3** | **7.05E-05** | **inf** | **2** |

| Structure reorganiation | Gene ID | Isoforms ID | FDR | Log_2_FC | Classify |
| --- | --- | --- | --- | --- | --- |
| cuticle protein 16.5, isoform A | evm.TU.scaffold_180.61 | PB.4536.1 | 0.007865 | 1.030988 | 2 |
|  |  | PB.4536.10 | 0.196032 | 0.786303 | 2 |
|  |  | PB.4536.11 |  |  | 2 |
|  |  | PB.4536.13 | 7.81E-05 | 1.198173 | 2 |
|  |  | **PB.4536.2** | **0.014347** | **6.485977** | 2 |
|  |  | PB.4536.3 |  |  | 2 |
|  |  | PB.4536.4 |  |  | 2 |
|  |  | PB.4536.5 | 0.03675 | 1.214117 | 2 |
|  |  | PB.4536.6 | 0.975562 | 0.204322 | 2 |
|  |  | PB.4536.7 |  |  | 2 |
|  |  | PB.4536.8 |  |  | 2 |
|  |  | PB.4536.9 |  |  | 2 |
|  |  |  |  |  |  |
| FH2 domain-containing protein 1-like | evm.TU.scaffold_370.5 | PB.9464.1 |  |  | 2 |
|  |  | **PB.9465.1** | **0.00332** | **-4.93456** | **2** |
|  |  | PB.9465.2 |  |  | 2 |
|  |  |  |  |  |  |
| filamin-B | evm.TU.scaffold_142.34 | **PB.3066.1** | **0.004543** | **-3.28209** | **2** |
|  |  | PB.3066.2 | 0.520708 | 0.454743 | 2 |
|  |  | PB.3067.1 |  |  | 2 |
|  |  |  |  |  |  |
| 5-azacytidine-induced protein 2 | evm.TU.scaffold_50.33 | PB.11117.2 | 0.892261 | 0.159366 | 1 |
|  |  | **PB.11117.1** | **0.007715** | **-3.16995** | **2** |
|  |  |  |  |  |  |
| nck-associated protein 1-like | evm.TU.scaffold_78.13 | PB.13364.2 | 0.412694 | 0.393585 | 1 |
|  |  | PB.13364.3 | 0.412694 | 0.393585 | 1 |
|  |  | **PB.13364.1** | **0.039325** | **-3.02486** | **2** |
|  |  |  |  |  |  |
| leucine-rich repeat and calponin homology domain-containing protein 1 | evm.TU.scaffold_149.3_evm.TU.scaffold_149.2 | PB.3274.1 | 0.946483 | 0.382861 | 2 |
|  |  | PB.3274.2 | 0.946483 | 0.088213 | 2 |
|  |  | **PB.3274.3** | **0.039827** | **2.608147** | **2** |
|  |  | PB.3274.4 | 0.832256 | 0.734802 | 2 |
|  |  | PB.3274.5 |  |  | 2 |
|  |  |  |  |  |  |
| coactosin-like protein | evm.TU.scaffold_103.13 | PB.890.3 | 0.958606 | 0.081019 | 1 |
|  |  | PB.889.1 |  |  | 2 |
|  |  | PB.890.1 | 0.808018 | -0.48943 | 2 |
|  |  | PB.890.2 | 0.856403 | -1.05787 | 2 |
|  |  | **PB.890.4** | **0.000604** | **-2.40801** | **2** |
|  |  | PB.890.5 |  |  | 2 |
|  |  |  |  |  |  |
| transmembrane protein 43 | evm.TU.scaffold_144.89 | PB.3165.1 | 0.015874 | -1.85567 | 2 |
|  |  | PB.3165.2 | 0.976289 | 0.316939 | 2 |
|  |  | **PB.3165.3** | **0.765221** | **-2.37708** | **2** |
|  |  |  |  |  |  |
| vinexin | evm.TU.scaffold_0.442 | **PB.243.1** | **0.045552** | **-2.00086** | **2** |
|  |  | PB.243.2 | 0.914522 | 0.123658 | 2 |
|  |  | PB.243.3 | 0.981039 | 0.130707 | 2 |
|  |  |  |  |  |  |
| alpha-actinin-1 | evm.TU.scaffold_236.36 | **PB.6425.1** | **0.000602** | **-1.97523** | **2** |
|  |  | PB.6425.10 |  |  | 2 |
|  |  | PB.6425.11 |  |  | 2 |
|  |  | PB.6425.12 |  |  | 2 |
|  |  | PB.6425.2 | 0.101778 | 0.749286 | 2 |
|  |  | PB.6425.3 |  |  | 2 |
|  |  | PB.6425.4 |  |  | 2 |
|  |  | PB.6425.6 | 0.761897 | 0.54235 | 2 |
|  |  | PB.6425.7 | 0.999544 | -0.01349 | 2 |
|  |  | PB.6425.8 | 0.855168 | 0.500007 | 2 |
|  |  | PB.6425.9 |  |  | 2 |
|  |  |  |  |  |  |
| Apolipoprotein D | evm.TU.scaffold_43.99 | **PB.10359.1** | **3.22E-05** | **-1.85612** | **2** |
|  |  |  |  |  |  |
| putative transmembrane protein | evm.TU.scaffold_22.94 | **PB.5956.1** | **0.021459** | **1.741839** | **2** |
|  |  | PB.5956.2 | 0.845781 | 0.213235 | 2 |
|  |  |  |  |  |  |
| WD repeat-containing protein 1-like | evm.TU.scaffold_44.12 | PB.10392.2 | 0.923864 | 0.413345 | 1 |
|  |  | PB.10392.1 |  |  | 2 |
|  |  | PB.10392.3 |  |  | 2 |
|  |  | PB.10392.4 | 0.110451 | 1.41548 | 2 |
|  |  | PB.10392.5 | 0.970685 | -0.58179 | 2 |
|  |  | PB.10392.6 | 0.9838 | 0.036635 | 2 |
|  |  | PB.10392.7 |  |  | 2 |
|  |  | **PB.10392.8** | **0.002708** | **-1.66423** | **2** |
|  |  |  |  |  |  |
| transmembrane protein 150A | evm.TU.scaffold_0.264 | PB.136.1 | 0.113261 | 0.831513 | 2 |
|  |  | **PB.136.2** | **0.00185** | **-1.63102** | **2** |
|  |  |  |  |  |  |
| wiskott-Aldrich syndrome protein | evm.TU.scaffold_11.189 | PB.1323.2 | 0.297189 | 0.729715 | 1 |
|  |  | PB.1323.1 | 0.997094 | -0.02 | 2 |
|  |  | **PB.1323.3** | **0.000988** | **-1.62098** | **2** |
|  |  |  |  |  |  |
| actin, cytoplasmic 2 | evm.TU.scaffold_94.66 | PB.14819.1 | 0.394971 | 0.585595 | 1 |
|  |  | PB.14819.2 | 0.882224 | -0.50939 | 2 |
|  |  | PB.14819.3 | 0.965341 | -0.08 | 2 |
|  |  | **PB.14819.4** | **0.005331** | **-1.61023** | **2** |
|  |  |  |  |  |  |
| WD repeat-containing protein WRAP73 | evm.TU.scaffold_5.69 | **PB.10925.1** | **0.031404** | **-1.61062** | **2** |
|  |  | PB.10925.2 |  |  | **2** |
|  |  |  |  |  |  |
| gamma-adducin | evm.TU.scaffold_69.37 | **PB.12519.1** | **0.044374** | **1.428625** | **2** |
|  |  | PB.12519.2 | 0.994263 | -0.03747 | 2 |
|  |  |  |  |  |  |
| beta tubulin | evm.TU.scaffold_0.71 | PB.43.3 | 0.868458 | 0.203772 | 1 |
|  |  | **PB.43.2** | **0.049742** | **-1.40334** | **2** |
|  |  | PB.43.4 | 0.791025 | -0.27178 | 2 |
|  |  |  |  |  |  |
| abl interactor 2 | evm.TU.scaffold_170.5 | PB.4228.2 | 0.862104 | -0.18388 | 1 |
|  |  | PB.4228.1 | 1 | 0.018813 | 2 |
|  |  | **PB.4228.3** | **0.022121** | **1.386298** | **2** |
|  |  | PB.4228.4 | 0.890442 | 0.678536 | 2 |
|  |  |  |  |  |  |
| cingulin-like protein 1 | evm.TU.scaffold_30.60 | PB.8261.1 | 0.181042 | 0.546222 | 1 |
|  |  | **PB.8261.2** | **0.044425** | **-1.29577** | **2** |
|  |  |  |  |  |  |
| mucin-5AC | evm.TU.scaffold_189.62 | PB.4795.2 | 0.869609 | -0.21125 | 2 |
|  |  | **PB.4795.3** | **0.00173** | **1.213369** | **2** |
|  |  |  |  |  |  |
| nidogen-1 | evm.TU.scaffold_10.96 | PB.663.1 | 0.602364 | 0.333211 | 2 |
|  |  | **PB.663.2** | **0.006389** | **-1.19517** | **2** |
|  |  |  |  |  |  |
| plectin | evm.TU.scaffold_34.57 | PB.8969.2 | 0.815377 | -0.35339 | 2 |
|  |  | **PB.8969.3** | **0.001362** | **-1.0525** | **2** |
|  |  |  |  |  |  |
| unconventional myosin-IXb | evm.TU.scaffold_59.127 | **PB.11798.1** | **0.011078** | **1.029461** | **1** |
|  |  | PB.11798.2 | 0.641076 | 0.303374 | 2 |
|  |  |  |  |  |  |
| multiple epidermal growth factor-like domains protein 9 | evm.TU.scaffold_0.94 | PB.51.1 | 0.994097 | -0.11798 | 2 |
|  |  | **PB.51.2** | **3.62E-08** | **inf** | **2** |
|  |  |  |  |  |  |
| plakophilin-3 | evm.TU.scaffold_233.52 | PB.6377.1 | 0.203193 | 0.967634 | 2 |
|  |  | PB.6377.2 |  |  | 2 |
|  |  | **PB.6377.3** | **0.002926** | **inf** | **2** |
|  |  | PB.6377.4 |  |  | 2 |
|  |  | PB.6377.6 |  |  | 2 |
|  |  | PB.6377.7 | 0.966397 | 0.16698 | 2 |
|  |  | PB.6377.8 |  |  | 2 |
|  |  | PB.6377.9 |  |  | 2 |
|  |  |  |  |  |  |
| actin filament-associated protein 1-like 1 | evm.TU.scaffold_27.139 | PB.7427.1 |  |  | 2 |
|  |  | **PB.7427.2** | **0.000192** | **inf** | **2** |
|  |  | PB.7427.3 | 0.987186 | 0.081245 | 2 |
|  |  | PB.7427.4 | 0.480591 | -1.95918 | 2 |
|  |  | PB.7427.5 | 0.828705 | -0.95603 | 2 |
|  |  | PB.7427.6 | 0.994535 | -0.14214 | 2 |
|  |  | PB.7427.7 | 0.977348 | -0.16421 | 2 |
|  |  | PB.7427.8 |  |  | 2 |

Classify represented the three types of isoforms, classify 1 was the known isoforms from annotated genes, classify 2 was the novel isoforms from annotated genes. ID: PB was the isoforms number from Iso-Seq, ID: evm.TU.scaffold was the Gene ID from reference genome, ID: evm.model.scaffold was the transcripts from reference genome.
